# Comparative Genomics Reveals the Genetic Mechanisms of Musk Secretion and Adaptive Immunity in Chinese Forest Musk Deer

**DOI:** 10.1093/gbe/evz055

**Published:** 2019-03-23

**Authors:** Chuang Zhou, Wenbo Zhang, Qinchao Wen, Ping Bu, Jie Gao, Guannan Wang, Jiazheng Jin, Yinjie Song, Xiaohong Sun, Yifan Zhang, Xue Jiang, Haoran Yu, Changjun Peng, Yongmei Shen, Megan Price, Jing Li, Xiuyue Zhang, Zhenxin Fan, Bisong Yue

**Affiliations:** 1 Key Laboratory of Bioresources and Ecoenvironment (Ministry of Education), College of Life Sciences, Sichuan University, Chengdu, P.R. China; 2 Sichuan Key Laboratory of Conservation Biology on Endangered Wildlife, College of Life Sciences, Sichuan University, Chengdu, P.R. China; 3 Sichuan Engineering Research Center for Medicinal Animals, Xichang, P.R. China; 4 Center of Infectious Diseases, West China Hospital, Sichuan University and Collaborative Innovation Center of Biotherapy, Chengdu, P.R. China

**Keywords:** *Moschus berezovskii*, musk secretion, adaptive immunity, positive selection, ADA gene, missense mutation

## Abstract

The Chinese forest musk deer (*Moschus berezovskii*; FMD) is an artiodactyl mammal and is both economically valuable and highly endangered. To investigate the genetic mechanisms of musk secretion and adaptive immunity in FMD, we compared its genome to nine other artiodactyl genomes. Comparative genomics demonstrated that eight positively selected genes (PSGs) in FMD were annotated in three KEGG pathways that were related to metabolic and synthetic activity of musk, similar to previous transcriptome studies. Functional enrichment analysis indicated that many PSGs were involved in the regulation of immune system processes, implying important reorganization of the immune system in FMD. FMD-specific missense mutations were found in two PSGs (*MHC* class II antigen *DRA* and *ADA*) that were classified as deleterious by PolyPhen-2, possibly contributing to immune adaptation to infectious diseases. Functional assessment showed that the FMD-specific mutation enhanced the *ADA* activity, which was likely to strengthen the immune defense against pathogenic invasion. Single nucleotide polymorphism-based inference showed the recent demographic trajectory for FMD. Our data and findings provide valuable genomic resources not only for studying the genetic mechanisms of musk secretion and adaptive immunity, but also for facilitating more effective management of the captive breeding programs for this endangered species.

## Introduction

The Chinese forest musk deer (*Moschus berezovskii*; FMD) is primarily found in Southern Asia and is well known for the secretion of musk (Xu et al. 2016). The musk is secreted from the musk gland of sexually mature males. Because of the distinctive fragrance and the significant role in antitumor, anti-inflammation, cardio-cerebral-vascular system, and central nervous system (Cao and Zhou 2007; Feng and Liu 2015), the musk has been a vital component of Chinese traditional medicine and perfume manufacturing (Sheng 1996; Su et al. 2001). Previous studies have explored the mechanisms of musk secretion based on anatomy (Bi et al. 1984; Bi and Shen 1986), mtDNA markers (Chen 2007; Zhao 2009), microsatellite (Guan et al. 2009), microbiota analysis (Li et al. 2016), and transcriptome (Xu et al. 2016), which illustrated various aspects of the secretory mechanisms of musk. However, the genetic mechanisms of musk secretion are still poorly understood.

As the global demand for the musk in the medicine and perfume industries has increased, the population of FMD has declined dramatically in the past five decades, and anthropogenic overexploitation and habitat destruction had placed FMD on the edge of extinction (Yang et al. 2003; Sheng and Liu 2007; He et al. 2014). In order to prevent the FMD from extinction, the Chinese government encouraged enterprises since the early 1950s to take part in captive breeding programs (Sun et al. 2018). However, the population growth of FMD had been limited, in part due to the high susceptibility to infectious diseases, particularly gastrointestinal diseases, respiratory illnesses, abscesses, and parasites (Xu et al. 2014). Previous studies investigating this issue found that the genetic diversity of the major histocompatibility complex (*MHC*) class II proteins were associated with the resistance or susceptibility of FMD to infectious diseases (Cai et al. 2015; Yao et al. 2015). One recent study based on the blood transcriptomes of purulent and healthy individuals detected that large numbers of the differentially expressed genes were related to the regulation of immune system processes, particularly in parasitic and bacterial infection pathways (Sun et al. 2018). The researchers suggested that abscesses in FMD were likely due to genetic deficiency or pathogenic invasion (Sun et al. 2018). However, the genetic diversity of the immune-related genes at the complete genome level of FMD has largely remained unexplored, thus it could be very useful to understand the mechanism of infectious disease formation in FMD.

Our laboratory sequenced and annotated the first complete genome sequence of one male FMD (Fan et al. 2018), which provided valuable resources to further investigate its genetic mechanisms of musk secretion and immunity. In this study, we compared the FMD genome with nine other artiodactyl species, aiming to address a number of key questions, namely 1) the phylogenetic relationship of artiodactyl species at the genomic level, 2) the genetic mechanisms of the musk secretion of FMD, and 3) the immune adaptation to common diseases of FMD.

## Materials and Methods

### Genome Data Collection

The full genome sequence of one male FMD was sequenced and annotated by our laboratory (Fan et al. 2018). In order to perform comparative genomic analysis, we collected the genomes of nine other Artiodactyla species from NCBI, including American bison (*Bison bison*, Bison UMD1.0), wild yak (*Bos mutus*, BosGru v2.0), water buffalo (*Bubalus bubalis*, UMD CASPUR WB 2.0), goat (*Capra hircus*, ARS1), white-tailed deer (*Odocoileus virginianus*, Ovir.te 1.0), sheep (*Ovis aries*, Oar v4.0), Tibetan antelope (*Pantholops hodgsonii*, PHO1.0), wild boar (*Sus scrofa*, Sscrofa11.1), and wild Bactrian camel (*Camelus ferus*, CB1).

### Gene Family, Phylogeny, and Divergence

We used orthoMCL (Li et al. 2003) to define orthologous genes from the ten Artiodactyla genomes (supplementary table S1, Supplementary Material online). The phylogenetic tree of these ten Artiodactyla species was constructed using the nucleotide sequences of 1:1 orthologous genes. Coding sequences from each 1:1 orthologous family were aligned with PRANK (Löytynoja and Goldman 2010), and were then concatenated to one sequence for each species to build the tree. Modeltest (ver. 3.7) was used to select the best substitution model for the whole concatenated sequence (Posada and Crandall 2005). RAxML (Stamatakis 2014) was applied to reconstruct maximum likelihood (ML) phylogenetic tree with 1,000 bootstrap replicates. Divergence time estimation was performed by the program Mcmctree within PAML (Yang 2007) with several calibration times (5–7 Ma for *Cap. hircus*/*Ov. aries*; 2.85–3.89 Ma for *Bo. mutus*/*Bi. bison*; 6.85–7.50 Ma for *Bo. mutus*/*Bu. bubalis*; 11.5–19.7 Ma for the emergence of Bovinae tribes) (Bai 2015; Tan et al. 2017). Analyses of gene family expansion and contraction were performed by CAFÉ (De Bie et al. 2006). GO enrichment analyses were performed according to previous studies (Huang et al. 2009; Chen et al. 2010).

### Positive Selection Analysis

The above alignments of 1:1 orthologous genes and phylogenetic tree were used to estimate the ratio of the rates of nonsynonymous to synonymous substitutions per gene (*ω*) by ML with the codeml program within PAML under the branch-site model (Yang 2007). We then performed a likelihood ratio test and identified positively selected genes (PSGs) FMD by means of FDR adjustment with *Q*-values <0.05. We conducted enrichment of the PSGs using KOBAS (Wu et al. 2006; Xie et al. 2011).

### FMD-Specific Missense Mutations

We examined the PSGs detected in FMD to identify FMD-specific missense mutations. The FMD-specific missense mutations were validated using three different approaches. Firstly, we downloaded all available protein sequences for each gene from mammals (*MHC* Class II Antigen *DRA*, supplementary table S2, Supplementary Material online; *MHC* Class II Antigen *DRB*, supplementary table S3, Supplementary Material online; *ADA*, supplementary table S4; Supplementary Material online), and some other vertebrates from Genbank. Together, these downloaded protein sequences and our study’s identified protein sequences genomes were aligned using MEGA7 (Kumar et al. 2016) for each gene to validate the FMD-specific missense mutations. Secondly, we checked the mutations based on the transcriptomes. The musk gland and heart tissue RNA-seq data (SRA accession: SRR2098995, SRR2098996, and SRR2142357) were downloaded, and then the short reads were de novo assembled into contigs using Trinity (Grabherr et al. 2011). On the basis of trinity results, we identified genes (*MHC* class II antigen *DRA*, *MHC* class II antigen *DRB*, and *ADA*) using TBlastN. Lastly, we performed PCR amplification and sequencing with eight FMD muscle samples to verify FMD-specific missense mutation sites in gene *MHC* class II antigen *DRA*. None of the FMD muscle samples was used for the genome sequencing, as they were collected from individuals from Miyaluo Farm (Sichuan Province, China). Primers (supplementary table S5, Supplementary Material online) were designed by *MHC* class II antigen *DRA* nucleotide sequences of FMD with Primer Premier 5 (Lalitha 2000). The amplification of genes was undertaken using TaKaRa RTaq (TaKaRa Biomedical, Japan) and implemented on a PTC-100 thermal cycler (BioRad, Hercules, CA) in the reaction mixture. The PCR products were sequenced on an ABI PRISM 3730 DNA sequencer at Tsingke Biotechnology Company (Chengdu, Sichuan Province, China) after electrophoresing in 2% agarose gel.

### Protein Structure Determination

The crystal structure of the *MHC* class II antigen *DRA* and *ADA* was obtained from SWISS-MODEL (Schwede et al. 2003). We converted the PDB files to PQR format with PDB2PQR server (Unni et al. 2011). The PDB files were further used for visualization of cartoon and surface representations of gene mutants. The visualization of the electrostatic surface potential was conducted using the APBS plugin in PyMOL (DeLano 2002).

### Construction of Recombinant pET28a(+)/*ADA* Expression Vector

The *ADA* gene synthesis and the pET-28a(+)/*ADA* construction was performed by Tsingke Biotechnology Company (Chengdu, China). The *ADA* gene and the vector pET28a (+) plasmid were subjected to digestion with Nco I and EcoR I, and ligated using T4 DNA ligase. The recombinant pET28a(+)/*ADA* was transformed into *Escherichiacoli* DH (5α) competent cells for amplification. Positive colonies resistant to ampicillin on a Luria–Bertani (LB) plate were selected and the plasmid pET28a(+)/*ADA* was confirmed by restriction enzyme mapping and DNA sequencing.

### Site-Directed Mutagenesis of *ADA* Gene

Mutations were generated via PCR using Taq polymerase with mutagenic primers (*ADA*-Ser-F [forward]: CCTGCCATTGC GGGC TGCCGGGAGG and *ADA*-Ser-R [reverse]: CATGTAG TAGTTGAACTTGGTCAGG, product size: 6,383 bp) (in which Pro located at position 70 was substituted by Ser by changing the TCT codon to CCT codon), with the recombinant plasmid containing *ADA* gene template. The amplification was carried out in 25 ml total volume containing 12.5 ml of I-5 2×High-Fidelity Master Mix (TSINGKE, Beijing, China), 1 μl of each primer (10 mM), and *ADA* gene template. The PCR reactions were performed through the following profile: An initial predenaturation for 5 min at 95 °C followed by 35 cycles at 95 °C—30s, 55 °C—30s, 72 °C—5 min, and a final extension at 72 °C for 10 min. Mutated PCR products were digested with dpnI and then we used the BKL Kit (Takara) to generate annular pET28a(+)/*ADA*-Pro70Ser plasmid. The recombinant pET28a(+)/*ADA*-Pro70Ser was transformed into *E. coli* DH (5α) competent cells for amplification. Positive colonies resistant to ampicillin on a Luria–Bertani (LB) plate were selected and the plasmid pET28a(+)/*ADA*-Pro70Ser was confirmed by restriction enzyme mapping and DNA sequencing.

### Expression of Recombinant Protein

The recombinant plasmids were extracted using a plasmid extraction kit (Tsingke Biotechnology Company, Chengdu, China), and then the recombinant plasmids pET28a(+)/*ADA* and pET28a(+)/*ADA*-Pro70Ser were transformed into *E. coli* BL21(DE3) (TransGen) competent cells for expression. The expression of the proteins was induced by the addition of 0.1 mM isopropyl-β-d-thiogalactoside (IPTG) once the optical density of 600 nm (OD600) of the culture had reached 0.6–0.8. After 4 h of induction, 1 ml culture was centrifuged at 8,000 × g for 5 min. The cell pellet was resuspended in 100 µl phosphate-buffered saline (PBS) and analyzed by SDS-PAGE. The expression level of recombinant *ADA* was determined by protein bands.

### Purification of Recombinant Protein

Following induction, 1 l culture was centrifuged at 8,000 × g for 5 min. The pellet was resuspended in 45 ml PBS and placed in an ice bath for ultrasonic lysis (200 W, 5 s, 5 s). The supernatant was further purified by 80% ammonium sulphate fractionation, dialysis and desalt, filtered using a 0.22 µm filtration membrane and loaded onto a Ni-NTA agarose column (Qiagen, Hilden, Germany). The column was washed with buffer (equilibration buffer) and the protein was eluted with elution buffer (50 mM sodium phosphate; pH 8.0; 0.3 M sodium chloride; and 250 mM imidazole). The eluate was concentrated through a 10 kDa cutoff Centriprep filter (Millipore, Bedford, CA, USA) for SDS-PAGE.

### Assay of *ADA* Activity

We assayed the *ADA* activity using a commercial kit (Nanjing Jiancheng Bioengineering Institute, Nanjing, China).

### Single Nucleotide Polymorphism Distribution and Demography from Genome Data

We used SAMtools to detect SNPs between diploid chromosomes for the sequenced FMD (Li et al. 2009). We used the pairwise sequentially markovian coalescent (PSMC) to infer the demographic history of FMD (Li and Durbin 2011). Briefly, the method employed the distribution of heterozygote sites across the FMD genome and a Hidden Markov Model to reconstruct the history of effective population sizes.

## Results

### Phylogeny, Divergence, and Gene Family

**Fig. 1.**
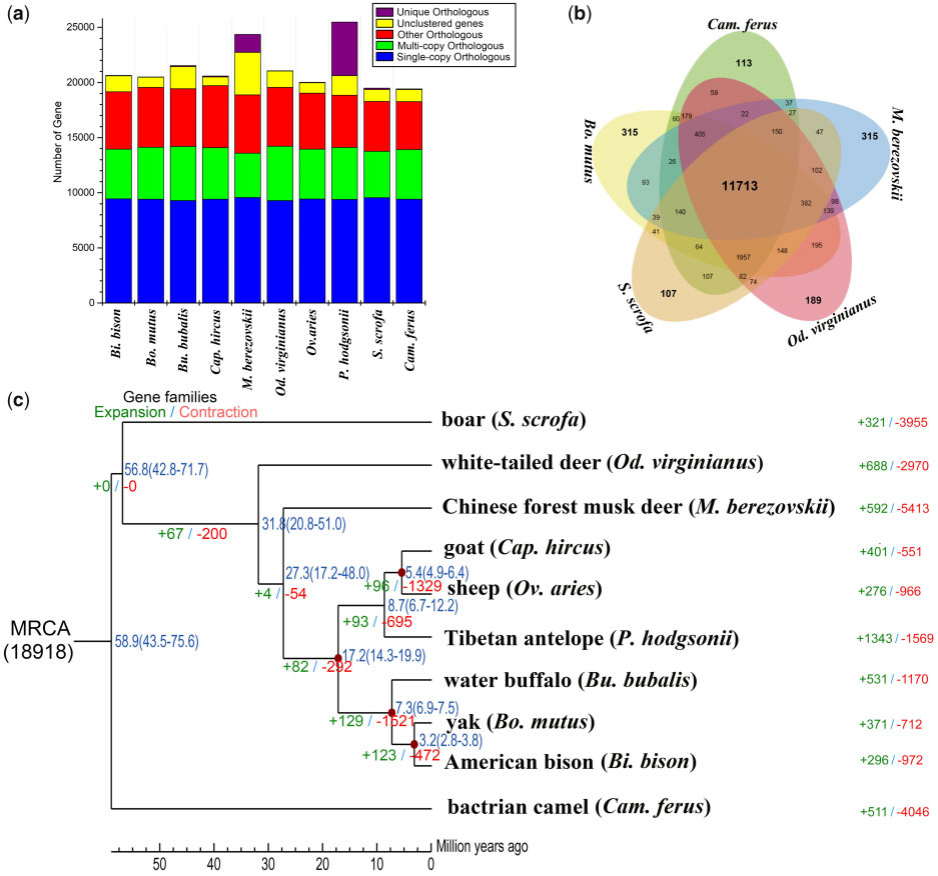
—Comparative genomics in ten Artiodactyla species studied. (*a*) Orthologous gene families of ten Artiodactyla species. (*b*) Comparison of orthologous gene clusters among *Bos mutus* (Bovidae), *Odocoileus virginianus* (Cervidae), *Sus scrofa* (Suidae), *Camelus ferus* (Camelidae), and FMD (Moschidae). (*c*) Phylogenetic tree constructed using 1:1 orthologous genes. Branch numbers indicate the number of gene families that have expanded (left) and contracted (right) after the split from the common ancestor. The time lines indicate divergence times among the species.

We identified 18,921 gene families for ten Artiodactyla species, of which 8,181 represented 1:1 orthologous gene families. In addition, there were 1,616 gene families specific to FMD (fig. 1*a*), whereas 5,156 gene families were found in the other nine Artiodactyla species but not in FMD. Furthermore, we identified 11,713 homologous gene families shared by *Bo. mutus* (Bovidae), *Od. virginianus* (Cervidae), *S.scrofa* (Suidae), *Cam. ferus* (Camelidae), and FMD (Moschidae), and 315 gene families were specific to FMD (fig. 1*b*). The ML phylogeny constructed based on the above 1:1 orthologous genes indicated that FMD and the Bovidae were within a subclade, which was most likely derived from a common ancestor ∼27.3 Ma. The series relationships within Artiodactyla were recovered as (Camelidae +  (Suidae +  (Cervidae +  (Moschidae + Bovidae)))) (fig. 1*c*).

Compared with other analyzed Artiodactyla species, 592 gene families were expanded, whereas 5,413 gene families were contracted in FMD. The functional analyses of gene families showed the expansions in FMD were enriched to several pathways associated with the immune response (supplementary table S6, Supplementary Material online), suggesting that these functions might be involved in FMD adaptive evolution. However, functional enrichment with contracted gene families also detected several immune response pathways that were possibly related to genetic immunodeficiency. In addition, contracted gene families in FMD were identified to be enriched in keratinization (supplementary table S7, Supplementary Material online), in agreement with FMD lacking horns (Kawtikwar et al. 2010). This enrichment in keratinization was different from some of the other Artiodactyla species.

### Positive Selection and Functional Enrichment

We found that 1,561 of the 8,181 1:1 orthologous genes were under positive selection in FMD. The Gene Ontology (GO) annotation classified the PSGs into three categories: Molecular functions, cellular components, and biological processes (supplementary fig. S1, Supplementary Material online). Molecular functions included genes mainly involved in binding (895 genes; GO: 0005488) and catalytic activity (408 genes; GO: 0003824). Genes related to cellular components were primarily cell (1,249 genes; GO: 0005623), cell part (1,247 genes; GO: 0044464), and organelle (1,021 genes; GO: 0043226). Biological process genes were mainly involved in cellular process (1,104 genes; GO: 0009987), biological regulation (787 genes; GO: 0065007), and metabolic process (773 genes; GO: 0008152). The distribution of GO annotations in different functional categories showed a substantial diversity of PSGs.

We identified the biochemical pathways on the basis of the PSGs detected in FMD. The KEGG annotation of the PSGs suggested that they were distributed in 43 pathways related to metabolism (108 genes), genetic information processing (111 genes), environmental information processing (143 genes), cellular processes (126 genes), organismal systems (171 genes), and human diseases (179 genes) (supplementary fig. S2*a*, Supplementary Material online). Among the identified functional categories, infectious diseases (98 genes) were highly represented (supplementary fig. S2*b*, Supplementary Material online).

We further performed the KEGG and GO enrichments with all the PSGs. The KEGG terms related to infectious diseases and immune response were overrepresented by PSGs, including primary immunodeficiency (corrected *P*-value: 0.000423), Cytokine-cytokine receptor interaction (corrected *P*-value: 0.002556), Measles (corrected *P*-value: 0.005083), Influenza A (corrected *P*-value: 0.006335), Malaria (corrected *P*-value: 0.023315), and Toll-like receptor signaling pathway (corrected *P*-value: 0.04422) (table 1).

**Table 1:** KEGG Enrichment of PSGs in FMD

Term	Input Number	Background Number	*P*-value	Corrected *P*-value
Primary immunodeficiency	10	39	1.76E–05	0.000423
Hematopoietic cell lineage	14	80	1.86E–05	0.000441
Cytokine-cytokine receptor interaction	23	225	0.00014	0.002556
Measles	16	135	0.00032	0.005083
Influenza A	18	168	0.000428	0.006335
Rheumatoid arthritis	12	89	0.000625	0.00852
Spliceosome	15	134	0.000831	0.010626
Cell adhesion molecules (CAMs)	16	152	0.001033	0.01233
Cytosolic DNA-sensing pathway	9	61	0.001665	0.01797
Adherens junction	10	74	0.001704	0.018314
Malaria	8	52	0.002354	0.023315
Homologous recombination	6	31	0.00301	0.028138
Cell cycle	13	124	0.003032	0.028313
Transcriptional misregulation in cancer	15	158	0.003631	0.031932
Peroxisome	10	83	0.003632	0.031932
Protein processing in endoplasmic reticulum	15	159	0.003833	0.033387
African trypanosomiasis	6	34	0.004497	0.038039
Fatty acid elongation	5	24	0.005077	0.04097
Graft-versus-host disease	6	35	0.005096	0.04097
Hepatitis B	14	150	0.005562	0.043544
Fatty acid metabolism	7	48	0.005638	0.043544
Toll-like receptor signaling pathway	11	104	0.005767	0.04422
FoxO signaling pathway	13	135	0.00584	0.044662
Amyotrophic lateral sclerosis (ALS)	7	50	0.006865	0.049574

GO enrichment identified significant overrepresentation of FMD PSGs involved in environmental adaptation (supplementary table S8, Supplementary Material online). A number of relevant observations from other studies on the activity of these genes hint at their possible functions. For instance, enzyme activator activity (GO: 0008047) could be affected by infection. The expression profile of pig lung tissues postinoculation with *Actinobacillus pleuropneumoniae* showed a significant representation of genes belonging to this GO term (Zuo et al. 2013). Endoplasmic reticulum (GO: 0005783) plays key roles in important processes like protein transport and energy metabolism. The mRNA expression of GO: 0005783 in mice is altered after heat treatment (Yu et al. 2011).

FMD was a nocturnal mammal and foraged during night or crepuscule (Qi et al. 2011). Currently, it was unclear how FMD evolved to acquire the nocturnal lifestyle. We observed eight PSGs (i.e., *GRK7*, *SLC24A1*, *SAG*, *CYP26A1*, *RDH10*, *RDH12*, *DGAT1*, and *SDR16C5*) distributed in phototransduction pathway and retinol metabolism pathways, possibly enhancing adaptation to low-light and night-time environments. In addition, males and females of FMD were visibly very similar, but male individuals were armed with two freakishly long canine teeth in the upper jaw used for fighting with rivals. We also found evidence of strong positive selection in four candidate genes (i.e., *KLK4*, *ACP4*, *CSF3R*, and *SSUH2*) associated with odontogenesis and amelogenesis.

### Genetic Mechanisms of Musk Secretion

We found six PSGs that were closely involved in related pathways (table 2). Firstly, one gene (*AKR1D1*) involved in steroid metabolism was annotated in steroid hormone biosynthesis (map00140) (supplementary fig. S3, Supplementary Material online). *AKR1D1* mainly inactivates the major classes of steroid hormones in steroid hormone metabolism and catalyze key steps of the biosynthetic pathway of bile acids, regulating lipid emulsification and cholesterol homoeostasis (Jin et al. 2014).

**Table 2:** KEGG Pathways Related to Musk Secretion

KEGG Pathway	Map ID	Number of Genes	Positively Selected Genes
Steroid hormone biosynthesis	map00140	1	*AKR1D1*
Terpenoid backbone biosynthesis	map00900	3	*MVD*
			*FNTA*
			*DHDDS*
Aldosterone-regulated sodium reabsorption	map04960	2	*SCNN1A*
			*FXYD4*

Furthermore, three PSGs (*MVD*, *FNTA*, and *DHDDS*) were annotated in terpenoid backbone biosynthesis (map00900) (supplementary fig. S4, Supplementary Material online), which are associated with terpenoid metabolism. *MVD* catalyses the final step in the mevalonate pathway, which is responsible for the biosynthesis of isoprenoids from acetate (Miziorko 2011). This pathway plays a key role in multiple cellular processes by synthesizing sterol isoprenoids, such as cholesterol (Buhaescu and Izzedine 2007; Miziorko 2011), and some cholesterols are converted to steroid hormones (Hinson et al. 1997). *DHDDS* catalyses the stepwise head-to-tail cis addition of isopentenyl-5-pyrophosphate to farnesyl-pyrophosphate, whereas farnesyl pyrophosphate is a key intermediate in cholesterol and sterol biosynthesis (Welti 2013; Xu et al. 2016). *FNTA* is another lipid metabolism-related gene (Jung et al. 2013). Additionally, we found two PSGs (*SCNN1A*, and *FXYD4*) related to aldosterone metabolism, which were annotated in aldosterone-regulated sodium reabsorption (map04960) (supplementary fig. S5, Supplementary Material online).

### Adaptive Immunity

Gastrointestinal disease is the most common disease in FMD (Wang and Meng 2014; Yan et al. 2016). We found that many PSGs were associated with gastrointestinal diseases, and they were distributed in six related pathways (inflammatory bowel disease [IBD], *Vibrio cholerae* infection, Epithelial cell signaling in *Helicobacter pylori* infection, pathogenic *E. coli* infection, *Salmonella* infection, and Shigellosis) (table 3). Previous studies have demonstrated that infections of *V.cholera*, pathogenic *E. coli*, *Salmonella*, and Shigellosis can cause diarrhoea, dysentery, and some other gastrointestinal disorders (Robins-Browne and Hartland 2002; Alam and Ashraf 2003; Alanis et al. 2005). Infection by *H. pylori* is likely to result in gastritis with stomach pains and nausea (Butcher 2003; Ryan and Ray 2014).

**Table 3:** KEGG Pathways Related to Common Diseases

Susceptible Diseases	KEGG Pathway	Map ID	Number of PSGs
Gastrointestinal diseases	Inflammatory bowel disease (IBD)	map05321	7
	*Vibrio cholerae* infection	map05110	2
	Epithelial cell signaling in *Helicobacter pylori* infection	map05120	6
	Pathogenic *Escherichia coli* infection	map05130	2
	Salmonella infection	map05132	9
	Shigellosis	map05131	4
Respiratory diseases	Asthma	map05310	1
	Pertussis	map05133	6
	Legionellosis	map05134	7
	Tuberculosis	map05152	14
	Measles	map05162	16
	Influenza A	map05164	18
Abscess disease	*Staphylococcus aureus* infection	map05150	3
Parasitic diseases	Amoebiasis	map05146	9
	Malaria	map05144	8
	Toxoplasmosis	map05145	7
	Leishmaniasis	map05140	6
	Chagas disease (American trypanosomiasis)	map05142	8
	African trypanosomiasis	map05143	6

Wang and Meng (2014) analyzed 339 dead FMDs, and they found respiratory diseases were the most common cause of mortality (31.27%). In the present study, several genes related to respiratory diseases were found to be under positive selection in FMD, which were distributed in six pathways (Asthma, Pertussis, Legionellosis, Tuberculosis, Measles, and Influenza A). Captive FMD breeding programs are particularly threatened by the prevalence of infectious diseases (Huang et al. 2016), with abscesses occupying 50% of all diagnoses (Luo et al. 2009; Zhao et al. 2011). We detected three PSGs in the *Staphylococcus aureus* infection pathway. *Staphylococcus aureus* was reported to be the most frequent cause of human skin or soft tissue abscesses (Lowy 1998; Cheng et al. 2009). Parasitic diseases are also important factors limiting the success of FMD captive breeding programs (Wang and Meng 2014; Yan et al. 2016). We found some PSGs associated with parasitic diseases in FMD, which were distributed in six pathways (Amoebiasis, Malaria, Toxoplasmosis, Leishmaniasis, Chagas disease [American trypanosomiasis], and African trypanosomiasis).

### FMD-Specific Missense Mutation

After the examination of the PSGs identified in FMD. We found two PSGs containing FMD-specific missense mutations. The first was at position 123 (Thr) of *MHC* class II antigen *DRA*, and the other was located at position 70 (Pro) of *ADA*.

**Fig. 2.**
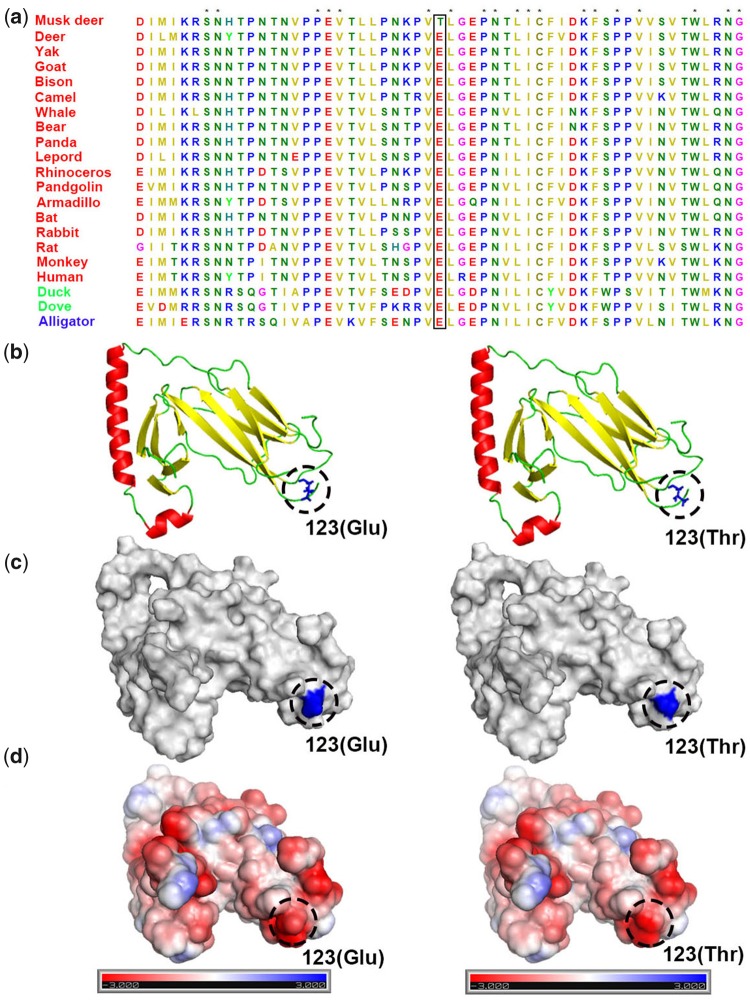
—Amino-acid sequence alignment and three kinds of visualization of nonmutated and mutated *MHC* class II antigen *DRA*. (*a*) The missense mutation in *MHC* class II antigen *DRA* is marked within rectangle. Asterisk means all species have the same amino acid type at this position. Species in red are mammals; species in green are birds; species in blue are reptiles. (*b*) Altered amino acid at p123 is shown in nonmutant and mutant *MHC* class II antigen *DRA* protein models. (*c*) In the surface of nonmutant and mutant *MHC* class II antigen *DRA*, mutation sites of p123 is colored as blue. (*d*) Electrostatic potential maps on the surface of p123 residues. Compared with nonmutant *MHC* class II antigen *DRA*, p123 mutation in mutant *MHC* class II antigen *DRA* shows a trend of negatively charged region (blue: positive charges; red: negative charges).

**Fig. 3.**
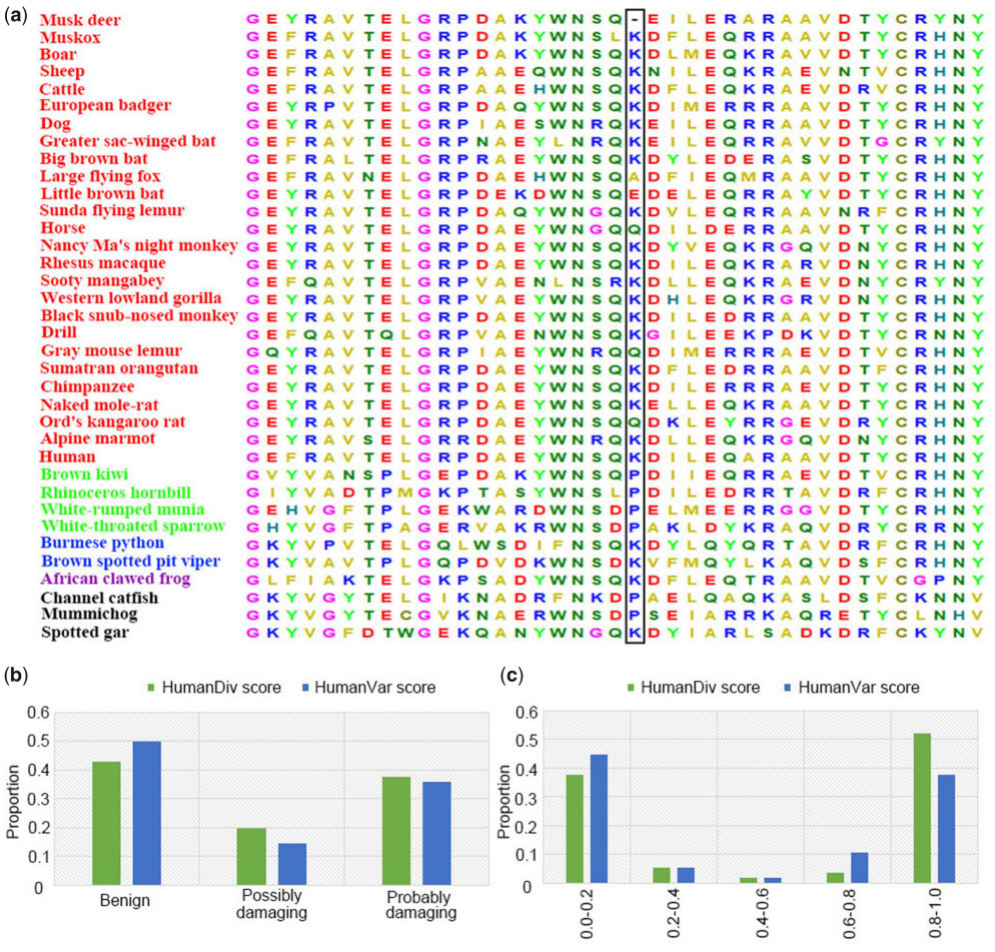
—Amino-acid sequence alignment and functional impact prediction of FMD-specific substitutions on human proteins for *MHC* class II antigen *DRB*. (*a*) The missense mutation in *MHC* class II antigen *DRB* is marked within rectangle. Asterisk means all species have the same amino acid type at this position. Species in red are mammals; species in green are birds; species in blue are reptiles; species in purple is Amphibia; species in black are Actinopterygii. (*b* and *c*) The functional classification and probability of being damaging for FMD-specific missense mutations located in *MHC* class II antigen *DRB*, according to the two metrics HumanDiv and HumanVar computed by PolyPhen-2.

The mutation at *MHC* class II antigen *DRA* (p.Glu123Thr) (fig. 2*a*) was classified as deleterious by PolyPhen-2 (Adzhubei et al. 2010), and produces a deleterious effect on protein structure (fig. 2*b*–*d*). The *MHC* class II antigen plays a fundamental role in controlling immune responses as well as in autoimmunity, vaccination and infection (Neefjes et al. 2011). *MHC* class II antigen *DRA* encodes the alpha subunit of *MHC* class II antigen *DR*, and pairs with the beta chain encoded by *MHC* class II antigen *DRB*. Therefore, we also examined whether *DRB* carried any specific mutations. There was one FMD-specific amino acid deletion in *MHC* class II antigen *DRB* that was situated at position 94 in humans (fig. 3*a*). We assessed the impact of FMD-specific substitutions on human proteins for *MHC* class II antigen *DRB* by computational predictions: A large proportion (∼50%) of mutations were predicted to be functionally damaging (fig. 3*b* and *c*).

**Fig. 4.**
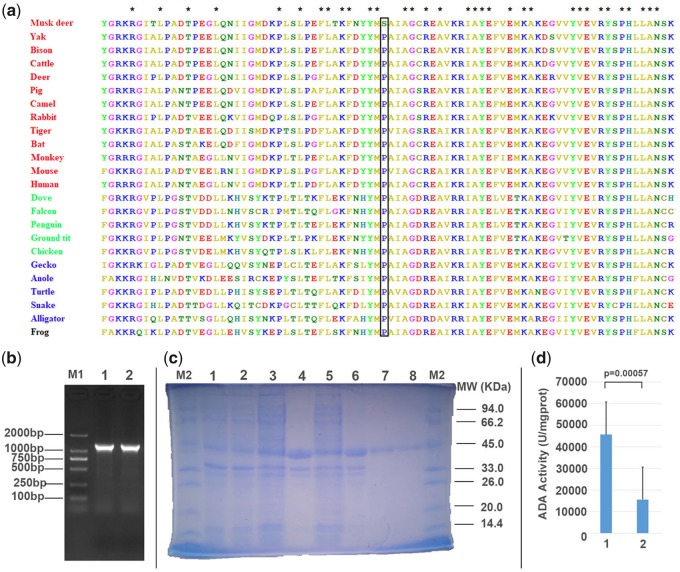
—Amino-acid sequence alignment and functional validation of FMD-specific mutation for *ADA*. (*a*) The missense mutation in *ADA* is marked within rectangle. Asterisk means all species have the same amino acid type at this position. Species in red are mammals; species in green are birds; species in blue are reptiles; species in black is Amphibia. (*b*) Identification of recombinant plasmid by plasmid polymerase chain reaction (PCR). Lane M1, DNA molecular weight standards; lane 1, PCR product of the wild *ADA* gene with the recombinant plasmid pET28a-*ADA* as template; lane 2, PCR product of the mutant *ADA* gene with the recombinant plasmid pET28a-*ADA* as template. (*c*) Expression of pET28a-*ADA* protein analyzed by SDS-PAGE. Lane M2: Protein molecular mass marker; lane 1: uninduced BL21(DE3)-pET28a-wild ADA (15 °C, 0.1 mM IPTG, 4 h); lane 2: uninduced BL21(DE3)-pET28a-mutant ADA (15 °C, 0.1 mM IPTG, 4 h); lane 3: supernatant of bacterial lysate (wild type, 15 °C, 0.1 mM IPTG, 4 h); lane 4: precipitation of bacterial lysate (wild type, 15 °C, 0.1 mM IPTG, 4 h); lane 5: supernatant of bacterial lysate (mutant type; 15 °C, 0.1 mM IPTG, 4 h); lane 6: precipitation of bacterial lysate (mutant type, 15 °C, 0.1 mM IPTG, 4 h); lane 7: purified pET28a-wild *ADA* (15 °C, 0.1 mM IPTG, 4 h); lane 8: purified pET28a-mutant *ADA* (15 °C, 0.1 mM IPTG, 4 h). (*d*) The *ADA* activity of mutant type is significantly less than that of wild type.

We found one FMD-specific missense mutation in the *ADA* gene (p.Pro70Ser) (fig. 4*a*) that was predicted to be damaging by PolyPhen-2. *ADA* is considered as one of the key enzymes of purine metabolism (Glader et al. 1983), and the high degree of amino acid sequence conservation suggests the crucial nature of *ADA* in the purine salvage pathway (Cristalli et al. 2001). Primarily, *ADA* in humans is involved in the development and maintenance of the immune system (Wilson et al. 1991).

### *E* *scherichia coli* Expression by the Missense Mutation Found in *ADA* of FMD

In order to explore whether the FMD-specific missense mutation (p.Pro70Ser) could affect the *ADA* activity, we conducted the *E. coli* expression of wild-type *ADA* (Ser) and mutated *ADA* (Pro). We found that the activity of mutated *ADA* was significantly lower than that of the wild type (supplementary table S9, Supplementary Material online and fig. 4*b*–*d*), which suggested that this missense mutation mediated the *ADA* activity of FMD, and possibly regulated the immune response.

### Demography Reconstruction

**Fig. 5.**
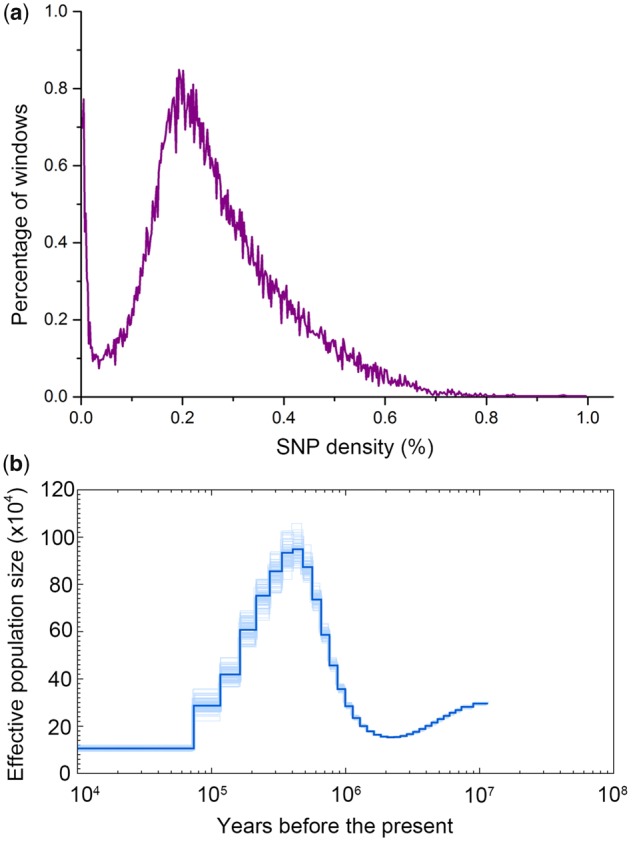
—SNP density distribution and demography reconstruction of FMD. (*a*) Distribution of SNP density across FMD genome. Heterozygous SNPs between the chromosomes were annotated, and heterozygosity density was observed in nonoverlapping 50-kb windows. (*b*) PSMC inference of FMD population history on basis of autosomal data. The central bold lines represent inferred population sizes. The 100 thin curves surrounding each line are the PSMC estimates which were generated using 100 sequences randomly resampled from the original sequence. The mutation rate on autosomes used in time scaling, was estimated using *Sus scrofa* autosome data.

We identified 7,015,181 heterozygous SNPs in the FMD genome. The distribution in genome-wide SNP density for FMD is shown in figure 5*a*. On the basis of local SNP densities, we performed PSMC modeling (Li and Durbin 2011) analysis to model the demographic history of FMD. We inferred demographic history between 10 million and 10,000 years ago. PSMC showed that FMD had experienced two bottlenecks (fig. 5*b*). The effective population size decreased from ∼300,000 individuals 10 Ma to a minimum of 150,000 individuals 2 Ma. Then the FMD populations started to expand, eventually giving rise to 950,000 individuals. However, FMD underwent a second bottleneck ∼400,000 years ago, and the population was reduced to 110,000 individuals.

## Discussion

### Phylogenetic Relationship

Complete genome sequences provided considerable resolution to the phylogenetic relationship and demography of FMD. The genome-wide phylogenetic tree showed that Cervidae (*Od. virginianus*) and Bovidae (*Bi. bison*, *Bo. mutus*, *Bu. bubalis*, *Cap. hircus*, *Ov. aries*, and *P. hodgsonii*) were more closely grouped, and FMD appeared to be their sister group. This genome-wide phylogenetic tree indicated that Moschidae, Cervidae, and Bovidae were three monophyletic groups. This phylogeny was consistent with other studies that placed Moschidae as an independent family (Li et al. 1998; Peng et al. 2009). Cervidae, Bovidae, and Moschidae originated from a common ancestor and Cervidae diverged earlier than Moschidae and Bovidae. However, previous phylogenetic studies of Artiodactyla species based on mitochondrial data found that Moschidae was the sister group of Cervidae, whereas other work reported that Moschidae was the sister group of Cervidae and Bovidae (Yang et al. 2012).

### Genetic Mechanisms of Musk Secretion

To investigate the musk secretion mechanisms in male FMD, it was crucial to understand their metabolic processes and the corresponding pathways and genes. So far, studies from different fields have been performed to explore the mechanisms of musk secretion (Chen 2007; Zhao 2009; Guan et al. 2009; Li et al. 2016; Xu et al. 2016). However, the mechanisms of musk secretion, especially the genetic mechanisms, are still poorly understood. Through comparative genomic analysis, six PSGs were annotated in three KEGG pathways that were related to metabolic and synthetic activity of musk. For example, we found PSGs related to steroid hormone biosynthesis, terpenoid backbone biosynthesis, and aldosterone-regulated sodium reabsorption, which were coincident with the study of Xu et al. (2016). *AKR1D1* was annotated in steroid hormone biosynthesis (map00140). *AKR1D1* was found to inactivate the major classes of steroid hormones in steroid hormone metabolism and catalyze key steps of the biosynthetic pathway of bile acids, regulating lipid emulsification, and cholesterol homoeostasis (Jin et al. 2014). Three PSGs (*MVD*, *FNTA*, and *DHDDS*) were annotated in terpenoid backbone biosynthesis (map00900) and participated in terpenoid metabolism. *MVD* catalyzed the final step in the mevalonate pathway, which was responsible for the biosynthesis of isoprenoids from acetate (Miziorko 2011). This pathway played a key role in multiple cellular processes by synthesizing sterol isoprenoids, such as cholesterol (Buhaescu and Izzedine 2007; Miziorko 2011), and some of the cholesterols were converted to steroid hormones (Hinson et al. 1997). *DHDDS* catalyzed the stepwise head-to-tail cis addition of isopentenyl-5-pyrophosphate to farnesyl-pyrophosphate, whereas farnesyl pyrophosphate was a key intermediate in cholesterol and sterol biosynthesis (Xu et al. 2016). *FNTA* was another lipid metabolism-related gene (Jung et al. 2013).

Previous studies based on transcriptomes (Xu et al. 2016, 2017) also detected many differentially expression genes (DEGs) in the above two pathways. Our results and theirs did not have overlap candidate genes, but clearly showed the pathways of steroid hormone biosynthesis and terpenoid backbone biosynthesis played a key role in regulating the musk secretion. Indeed, genes implicated in reproduction regulation pathways such as steroid hormone biosynthesis were found in hens with different laying rates (Chen et al. 2007) and Huoyan goose ovaries between the laying period and ceased period (Luan et al. 2014). In order to understand the process of steroid-mediated oocyte maturation, it is necessary to inspect the steroid production and steroid signaling. Previous studies have indicated that steroid production could be induced by gonadotropin, and then steroid can facilitate the maturation of oocyte (Evaul 2009). Steroid metabolism plays an indispensable role in manipulating reproduction, and is closely related to the genital gland, and the metabolism of these compounds is critical to the reproduction. As for the male FMD, the musk gland is situated between naval and genital and is a representative sexual character of this endangered species. The musk gland produces chemical compounds, of which muscone and cholesterol are the primary composition of musk, and then manipulates the composition, formation, and secretion of musk.

### Adaptive Immunity and Diseases

Comparative genomics identified many PSGs involved in diseases and the immune response to viral, bacterial and parasitic diseases indicating the susceptibility of FMD to gastrointestinal diseases, respiratory diseases, abscess diseases, and parasitic diseases. In particular, three immune-related genes (*MHC* class II antigen *DRA*, *MHC* class II antigen *DRB*, and *ADA*), which had FMD-specific mutations, have been demonstrated to be associated with the immune response (Wilson et al. 1991; Neefjes et al. 2011). The *E. coli* expression results suggested that the FMD-specific mutation of *ADA* contributed to the increase of *ADA* activity, which was likely to enhance the immune response to infectious diseases and could be considered as the evolutionary immune adaptation of FMD (Arredondo-Vega et al. 2002). Moreover, the identified expansions in gene families associated with the immune response, immune system process and defense response in FMD implicated that the immune adaptation had been to the diseases that FMD exhibited the greatest susceptibility. Meanwhile, we found some contracted gene families that were also functionally related to the immune response. These results suggest that some of these common diseases were likely to be caused by genetic deficiency or pathogenic invasion. Further study should focus on these immune-related genes found in the present study, because these genes probably provide us useful clues for further understanding the mechanisms of immune system development in FMD.

### Other Adaptive Traits

We observed eight PSGs (*GRK7*, *SLC24A1*, *SAG*, *CYP26A1*, *RDH10*, *RDH12*, *DGAT1*, and *SDR16C5*) distributed in phototransduction (map04744) and retinol metabolism pathways (map00830) (Hemond and Vollmer 2015; Wu et al. 2016). Previous studies have reported that mutations in *SLC24A1* were implicated in Autosomal-Recessive Congenital Stationary Night Blindness (CSNB) (Riazuddin et al. 2010). *SAG* was involved in rod vision, which encoded S-arrestin protein, a major soluble photoreceptor protein related to the desensitization of the photoactivated transduction cascade (Shen et al. 2012). Mutations in *SAG* were also associated with night blindness (Isashiki et al. 1999). *RDH12* encoded a photoreceptor cell retinol dehydrogenase, and mutations at this gene were found to be associated with Leber congenital amaurosis and cone-rod dystrophy (Sun et al. 2007). Therefore, these genes may be contributed to the nocturnality of FMD. FMD is largely nocturnal but they can also be active during twilight (Qi et al. 2011). Currently, it is unclear how FMD acquired these pathways/adaptations but being nocturnal may avoid diurnal predators, such as humans and/or environmental conditions.

We found evidence of strong positive selection in four candidate genes (*KLK4*, *ACP4*, *CSF3R*, and *SSUH2*) that were associated with odontogenesis and amelogenesis. *KLK4* (Kallikrein-4) is a glycosylated chymotrypsin-like serine protease and has been identified as essential participants in amelogenesis (Hart et al. 2004). Mutations in *KLK4* can lead to autosomal recessive, nonsyndromic enamel malformations in humans and mice (Bartlett and Simmer 2014). *ACPT* localization has been detected in secretory ameloblasts, odontoblasts, and osteoblasts, and its biallelic mutations could cause nonsyndromic, generalized hypoplastic autosomal-recessive amelogenesis imperfecta in humans (Seymen et al. 2016). After transplantation of pulp stem cells with granulocyte-colony stimulating factor in a dog pulpectomized tooth, the root canal was completely full of regenerated pulp tissue including vasculature and innervation, and the coronal part was filled with regenerated dentin (Iohara et al. 2013). *SSUH2* disrupts dental formation and was involved in tooth development (Xiong et al. 2017). Males and females of FMD are generally morphologically similar, but males are armed with two long canine teeth in the upper jaw to fight rivals. Therefore, the strong positive selection of these genes associated with odontogenesis and amelogenesis may enhance the formation of long canine teeth in male FMD.

In summary, this is the first report conducting the comparative genomics of FMD to other Artiodactyla species. Comparative genomics confirmed the PSGs annotated in functional pathways related to steroid compounds metabolism, such as steroid hormone biosynthesis, were present in FMD. The obtained PSGs data provide comprehensive gene information at the genomic level, which can contribute to a better understanding of the genetic mechanisms of musk formation and secretion in FMD. Our identification of the PSGs related to the common susceptible diseases in FMD provide a framework for future studies of these diseases in this species. Furthermore, our work can help enhance the understanding of the pathogenesis and immune system function of FMD-susceptible diseases. The PSGs found in FMD in this study will provide a strong foundation for other functional and comparative genomic studies.

## Supplementary Material

Supplementary data are available at *Genome Biology and Evolution* online.

## Supplementary Material

Supplementary Data
