# Supplementary material for: Comparative Genomics Reveals the Genetic Mechanisms of Musk Secretion and Adaptive Immunity in Chinese Forest Musk Deer
Source: Genome Biol Evol. 2019 Mar 23;11(4):1019–32. doi: 10.1093/gbe/evz055 (PMC6450037; doi:10.1093/gbe/evz055)
Supplement: Supplementary Data [file evz055_supp.docx]

**Supplementary material**

**Comparative Genomics Reveals the Genetic Mechanisms of Musk Secretion and Adaptive Immunity in Chinese Forest Musk Deer**

Chuang Zhou^1^, Wenbo Zhang^1^, Qinchao Wen^2^, Ping Bu^1^, Jie Gao^2^, Guannan Wang^1^, Jiazheng Jin^3^, Yinjie Song^4^, Xiaohong Sun^1^, Yifan Zhang^2^, Xue Jiang^3^, Haoran Yu^1^, Changjun Peng^1^, Yongmei Shen^3^, Megan Price^1^, Jing Li^1^, Xiuyue Zhang^2^, Zhenxin Fan^1,*^, Bisong Yue^1,*^

^1^ Key Laboratory of Bioresources and Ecoenvironment (Ministry of Education), College of Life Sciences, Sichuan University, Chengdu, 610064, P.R. China

^2^ Sichuan Key Laboratory of Conservation Biology on Endangered Wildlife, College of Life Sciences, Sichuan University, Chengdu, 610064, P.R. China

^3^ Sichuan Engineering Research Center for Medicinal Animals, Xichang 615000, People’s Republic of China

^4^ Center of Infectious Diseases, West China Hospital, Sichuan University and Collaborative Innovation Center of Biotherapy, Chengdu, 610041, P.R. China

Corresponding author: Zhenxin Fan (zxfan@scu.edu.cn), Bisong Yue (bsyue@scu.edu.cn)

# Supplementary Tables

| **Supplementary Table S1. The Genome Information of Ten Artiodactyla Species Used in This Study.** | | | |
| --- | --- | --- | --- |
| **Common name** | **Scientific name** | **Family** | **Assembly ID** |
| American bison | *Bison bison* | Bovidae | Bison_UMD1.0 |
| Wild yak | *Bos mutus* |  | BosGru_v2.0 |
| Water buffalo | *Bubalus bubalis* |  | UMD_CASPUR_WB_2.0 |
| Goat | *Capra hircus* |  | ARS1 |
| Dwarf musk deer | *Moschus berezovskii* | Moschidae | this study |
| White-tailed deer | *Odocoileus virginianus* | Cervidae | Ovir.te_1.0 |
| Sheep | *Ovis aries* | Bovidae | Oar_v4.0 |
| Tibetan antelope | *Pantholops hodgsonii* |  | PHO1.0 |
| Wild boar | *Sus scrofa* | Suidae | Sscrofa11.1 |
| Wild Bactrian camel | *Camelus ferus* | Camelidae | CB1 |

**Supplementary Table S2. Protein Sequences of Mammals Used to Check FMD-specific Mutation in *MHC* Class II Antigen *DRA*.**

| **Accession ID** | **Species** | **Family** | **Order** |
| --- | --- | --- | --- |
| XP_014943173.1 | *Acinonyx jubatus* | Felidae | Carnivora |
| XP_002928296.1 | *Ailuropoda melanoleuca* | Ursidae | Carnivora |
| XP_12296104.1 | *Aotus nancymaae* | Aotidae | Primates |
| XP_7193950.1 | *Balaenoptera acutorostrata* | Balaenopteridae | Cetacea |
| XP_10843572.1 | *Bison bison bison* | Bovidae | Artiodactyla |
| XP_19841331.1 | *Bos indicus* | Bovidae | Artiodactyla |
| XP_5910934.1 | *Bos mutus* | Bovidae | Artiodactyla |
| NP_1012695.1 | *Bos taurus* | Bovidae | Artiodactyla |
| JAB18165.1 | *Callithrix jacchus* | Callitrichidae | Primates |
| NP_1301117.1 | *Capra hircus* | Bovidae | Artiodactyla |
| XP_8067963.1 | *Carlito syrichta* | Tarsiidae | Primates |
| XP_20007188.1 | *Castor canadensis* | Castoridae | Rodentia |
| XP_3473989.1 | *Cavia porcellus* | Caviidae | Rodentia |
| XP_17378236.1 | *Cebus capucinus imitator* | Cebidae | Primates |
| XP_14638349.1 | *Ceratotherium simum simum* | Rhinocerotidae | Perissodactyla |
| XP_6860398.1 | *Chrysochloris asiatica* | Chrysochloridae | Afrosoricida |
| XP_4695845.1 | *Condylura cristata* | Talpidae | Eulipotyphla |
| XP_12383353.1 | *Dasypus novemcinctus* | Dasypodidae | Cingulata |
| XP_22437859.1 | *Delphinapterus leucas* | Cetacea | Artiodactyla |
| JAA45987.1 | *Desmodus rotundus* | Phyllostomidae | Chiroptera |
| XP_12890473.1 | *Dipodomys ordii* | Heteromyidae | Rodentia |
| XP_22366303.1 | *Enhydra lutris kenyoni* | Mustelidae | Carnivora |
| XP_8155535.1 | *Eptesicus fuscus* | Vespertilionidae | Chiroptera |
| NP_1295980.1 | *Felis catus* | Felidae | Carnivora |
| XP_4043811.1 | *Gorilla gorilla gorilla* | Hominidae | Primates |
| EHB16100.1 | *Heterocephalus glaber* | Heterocephalidae | Rodentia |
| XP_19482164.1 | *Hipposideros armiger* | Hipposideridae | Chiroptera |
| NP_61984.2 | *Homo sapiens* | Hominidae | Primates |
| XP_12807522.1 | *Jaculus jaculus* | Dipodidae | Rodentia |
| XP_6736305.1 | *Leptonychotes weddellii* | Phocidae | Carnivora |
| XP_7459568.1 | *Lipotes vexillifer* | Lipotidae | Artiodactyla |
| AFE67067.1 | *Macaca mulatta* | Cercopithecidae | Primates |
| XP_17536876.1 | *Manis javanica* | Manidae | Pholidota |
| XP_16077385.1 | *Miniopterus natalensis* | Miniopteridae | Chiroptera |
| XP_12906200.1 | *Mustela putorius furo* | Mustelidae | Carnivora |
| XP_5872269.1 | *Myotis brandtii* | Vespertilionidae | Chiroptera |
| XP_6105212.1 | *Myotis lucifugus* | Vespertilionidae | Chiroptera |
| XP_8841045.1 | *Nannospalax galili* | [Spalacidae](http://www.uniprot.org/taxonomy/337664) | [Rodentia](https://en.wikipedia.org/wiki/Rodent) |
| XP_21552737.1 | *Neomonachus schauinslandi* | Phocidae | Carnivora |
| XP_3272196.1 | *Nomascus leucogenys* | Hylobatidae | Primates |
| XP_4410689.1 | *Odobenus rosmarus divergens* | Odobenidae | Carnivora |
| XP_20772115.1 | *Odocoileus virginianus* | Cervidae | Artiodactyla |
| XP_4285671.1 | *Orcinus orca* | Delphinidae | Artiodactyla |
| XP_7937577.1 | *Orycteropus afer afer* | [Orycteropodidae](https://en.wikipedia.org/wiki/Orycteropodidae) | [Tubulidentata](https://en.wikipedia.org/wiki/Orycteropodidae) |
| NP_1164589.1 | *Oryctolagus cuniculus* | Leporidae | Lagomorpha |
| XP_3789115.1 | *Otolemur garnettii* | Galagidae | Primates |
| XP_3829807.1 | *Pan paniscus* | Hominidae | Primates |
| NP_1127811.1 | *Pan troglodytes* | Hominidae | Primates |
| XP_19294643.1 | *Panthera pardus* | Felidae | Carnivora |
| XP_7093322.1 | *Panthera tigris altaica* | Felidae | Carnivora |
| XP_7118658.1 | *Physeter catodon* | Physeteridae | Artiodactyla |
| XP_2809188.2 | *Pongo abelii* | Hominidae | Primates |
| ELK16655.1_ | *Pteropus alecto* | Pteropodidae | Chiroptera |
| XP_11378080.1 | *Pteropus vampyrus* | Pteropodidae | Chiroptera |
| XP_19571189.1 | *Rhinolophus sinicus* | Rhinolophidae | Chiroptera |
| XP_15993109.1 | *Rousettus aegyptiacus* | Pteropodidae | Chiroptera |
| XP_3770700.1 | *Sarcophilus harrisii* | Dasyuridae | Dasyuromorphia |
| XP_12790231.1 | *Sorex araneus* | Soricidae | Eulipotyphla |
| XP_4391142.1 | *Trichechus manatus* | Trichechidae | Sirenia |
| XP_19805299.1 | *Tursiops truncatus* | Delphinidae | Artiodactyla |
| XP_8691695.1 | *Ursus maritimus* | Ursidae | Carnivora |

**Supplementary Table S3. Protein Sequences of Mammals Used to Check FMD-specific Mutation in *MHC* Class II Antigen *DRB*.**

| **Accession ID** | **Species** | **Family** | **Order** |
| --- | --- | --- | --- |
| XP_15342994.1 | *Marmota marmota* | Sciuridae | Rodentia |
| NP_1012698.2 | *Bos taurus* | Bovidae | Artiodactyla |
| NP_1266704.1 | *Pan troglodytes* | Hominidae | Primates |
| CAH61725.1 | *Canis lupus familiaris* | Canidae | Carnivora |
| AET36869.1 | *Meles meles* | Mustelidae | Carnivora |
| AFG31697.1 | *Saccopteryx bilineata* | Emballonuridae | Chiroptera |
| NP_1136283.1 | *Equus caballus* | Equidae | Perissodactyla |
| AAR84662.1 | *Aotus nancymaae* | Aotidae | Primates |
| AAK67170.2 | *Ovibos moschatus* | Bovidae | Artiodactyla |
| JAN98415.1 | *Heterocephalus glaber* | Heterocephalidae | Rodentia |
| XP_12890411.1 | *Dipodomys ordii* | Heteromyidae | Rodentia |
| ABK96888.1 | *Sus scrofa* | Suidae | Artiodactyla |
| AAB63306.1 | *Macaca mulatta* | Cercopithecidae | Primates |
| NP_1295506.1 | *Ovis aries* | Bovidae | Artiodactyla |
| XP_11925462.1 | *Cercocebus atys* | Cercopithecidae | Primates |
| XP_18884635.1 | *Gorilla gorilla gorilla* | Hominidae | Primates |
| XP_8155622.1 | *Eptesicus fuscus* | Vespertilionidae | Chiroptera |
| XP_17714647.1 | *Rhinopithecus bieti* | Cercopithecidae | Primates |
| XP_11830813.1 | *Mandrillus leucophaeus* | Cercopithecidae | Primates |
| NP_1296851.1 | *Microcebus murinus* | Cheirogaleidae | Primates |
| XP_11383648.1 | *Pteropus vampyrus* | Pteropodidae | Chiroptera |
| XP_6105637.1 | *Myotis lucifugus* | Vespertilionidae | Chiroptera |
| XP_3776854.2 | *Pongo abelii* | Hominidae | Primates |
| XP_8562610.1 | *Galeopterus variegatus* | Cynocephalidae | Dermoptera |

**Supplementary Table S4. Protein Sequences of Mammals Used to Check FMD-specific Mutation in *ADA*.**

| **Accession ID** | **Species** | **Family** | **Order** |
| --- | --- | --- | --- |
| XP_007193423.1 | *Balaenoptera acutorostrata* | Balaenopteridae | Cetacea |
| XP_010839283.1 | *Bison bison* | Bovidae | Artiodactyla |
| ELR50965.1 | *Bos mutus* | Bovidae | Artiodactyla |
| AAF91430.1 | *Bos taurus* | Bovidae | Artiodactyla |
| XP_010976026.1 | *Camelus dromedarius* | [Camelidae](https://en.wikipedia.org/wiki/Camelid) | [Artiodactyla](https://en.wikipedia.org/wiki/Even-toed_ungulate) |
| EPY74194.1 | *Camelus ferus* | [Camelidae](https://en.wikipedia.org/wiki/Camelid) | [Artiodactyla](https://en.wikipedia.org/wiki/Even-toed_ungulate) |
| AAD40577.1 | *Canis lupus familiaris* | [Canidae](https://en.wikipedia.org/wiki/Canidae) | [Carnivora](https://en.wikipedia.org/wiki/Carnivora) |
| XP_008065101.1 | *Carlito syrichta* | Tarsiidae | Primates |
| JAV42750.1 | *Castor canadensis* | Castoridae | Rodentia |
| AKZ42359.1 | *Castor fiber* | [Castoridae](https://en.wikipedia.org/wiki/Castoridae) | [Rodentia](https://en.wikipedia.org/wiki/Rodent) |
| XP_014642921.1 | *Ceratotherium simum* | Rhinocerotidae | Perissodactyla |
| XP_005392524.1 | *Chinchilla lanigera* | [Chinchillidae](https://en.wikipedia.org/wiki/Chinchillidae) | [Rodentia](https://en.wikipedia.org/wiki/Rodent) |
| XP_006839410.1 | *Chrysochloris asiatica* | Chrysochloridae | Afrosoricida |
| XP_012583573.1 | *Condylura cristata* | Talpidae | Eulipotyphla |
| EGV98286.1 | *Cricetulus griseus* | [Cricetidae](https://en.wikipedia.org/wiki/Cricetidae) | [Rodentia](https://en.wikipedia.org/wiki/Rodent) |
| XP_023447632.1 | *Dasypus novemcinctus* | Dasypodidae | Cingulata |
| XP_024415238.1 | *Desmodus rotundus* | Phyllostomidae | Chiroptera |
| XP_004698336.1 | *Echinops telfairi* | [Tenrecidae](https://en.wikipedia.org/wiki/Tenrecidae) | [Afrosoricida](https://en.wikipedia.org/wiki/Afrosoricida) |
| XP_006881786.1 | *Elephantulus edwardii* | [Macroscelididae](https://en.wikipedia.org/wiki/Macroscelididae) | [Macroscelididae](https://en.wikipedia.org/wiki/Macroscelididae) |
| XP_022349612.1 | *Enhydra lutris kenyoni* | Mustelidae | Carnivora |
| XP_008159208.1 | *Eptesicus fuscus* | Vespertilionidae | Chiroptera |
| XP_016047086.1 | *Erinaceus europaeus* | [Erinaceidae](https://en.wikipedia.org/wiki/Erinaceidae) | [Eulipotyphla](https://en.wikipedia.org/wiki/Eulipotyphla) |
| KFO30240.1 | *Fukomys damarensis* | [Bathyergidae](https://en.wikipedia.org/wiki/Bathyergidae) | [Rodentia](https://en.wikipedia.org/wiki/Rodent) |
| XP_008589470.1 | *Galeopterus variegatus* | [Cynocephalidae](https://en.wikipedia.org/wiki/Cynocephalidae) | [Dermoptera](https://en.wikipedia.org/wiki/Dermoptera) |
| EHB06499.1 | *Heterocephalus glaber* | Heterocephalidae | Rodentia |
| AAN10291.1 | *Homo sapiens* | Hominidae | Primates |
| XP_021583277.1 | *Ictidomys tridecemlineatus* | [Sciuridae](https://en.wikipedia.org/wiki/Sciuridae) | [Rodentia](https://en.wikipedia.org/wiki/Rodent) |
| XP_004669333.1 | *Jaculus jaculus* | Dipodidae | Rodentia |
| XP_006735318.1 | *Leptonychotes weddellii* | Phocidae | Carnivora |
| XP_007464838.1 | *Lipotes vexillifer* | Lipotidae | Artiodactyla |
| EHH65319.1 | *Macaca fascicularis* | [Cercopithecidae](https://en.wikipedia.org/wiki/Old_World_monkey) | [Primates](https://en.wikipedia.org/wiki/Primate) |
| AFH27578.1 | *Macaca mulatta* | Cercopithecidae | Primates |
| XP_011853020.1 | *Mandrillus leucophaeus* | Cercopithecidae | Primates |
| XP_015336614.1 | *Marmota marmota* | [Sciuridae](https://en.wikipedia.org/wiki/Squirrel) | [Rodentia](https://en.wikipedia.org/wiki/Rodent) |
| XP_021491987.1 | *Meriones unguiculatus* | [Muridae](https://en.wikipedia.org/wiki/Muridae) | [Rodentia](https://en.wikipedia.org/wiki/Rodent) |
| XP_021091562.1 | *Mesocricetus auratus* | [Cricetidae](https://en.wikipedia.org/wiki/Cricetidae) | [Rodentia](https://en.wikipedia.org/wiki/Rodent) |
| XP_016074864.1 | *Miniopterus natalensis* | Miniopteridae | Chiroptera |
| XP_007476984.1 | *Monodelphis domestica* | [Didelphidae](https://en.wikipedia.org/wiki/Didelphidae) | [Didelphimorphia](https://en.wikipedia.org/wiki/Didelphimorphia) |
| XP_021011168.1 | *Mus caroli* | [Muridae](https://en.wikipedia.org/wiki/Muridae) | [Rodentia](https://en.wikipedia.org/wiki/Rodent) |
| AAL01302.1 | *Mus musculus* | [Muridae](https://en.wikipedia.org/wiki/Muridae) | [Rodentia](https://en.wikipedia.org/wiki/Rodent) |
| XP_021049951.1 | *Mus pahari* | [Muridae](https://en.wikipedia.org/wiki/Muridae) | [Rodentia](https://en.wikipedia.org/wiki/Rodent) |
| AER93702.1 | *Mustela putorius furo* | Mustelidae | Carnivora |
| EPQ10767.1 | *Myotis brandtii* | Vespertilionidae | Chiroptera |
| ELK30239.1 | *Myotis davidii* | [Vespertilionidae](https://en.wikipedia.org/wiki/Vespertilionidae) | [Chiroptera](https://en.wikipedia.org/wiki/Chiroptera) |
| XP_008849240.1 | *Nannospalax galili* | [Spalacidae](http://www.uniprot.org/taxonomy/337664) | [Rodentia](https://en.wikipedia.org/wiki/Rodent) |
| XP_021544790.1 | *Neomonachus schauinslandi* | Phocidae | Carnivora |
| CCP86853.1 | *Neovison vison* | [Mustelidae](https://en.wikipedia.org/wiki/Mustelidae) | [Carnivora](https://en.wikipedia.org/wiki/Carnivora) |
| XP_004586118.1 | *Ochotona princeps* | [Ochotonidae](https://en.wikipedia.org/wiki/Ochotonidae) | [Lagomorpha](https://en.wikipedia.org/wiki/Lagomorpha) |
| XP_023570609.1 | *Octodon degus* | [Octodontidae](https://en.wikipedia.org/wiki/Octodontidae) | [Rodentia](https://en.wikipedia.org/wiki/Rodent) |
| XP_004414158.1 | *Odobenus rosmarus divergens* | Odobenidae | Carnivora |
| XP_020767122.1 | *Odocoileus virginianus* | Cervidae | Artiodactyla |
| XP_007663965.1 | *Ornithorhynchus anatinus* | [Ornithorhynchidae](https://en.wikipedia.org/wiki/Ornithorhynchidae) | [Monotremata](https://en.wikipedia.org/wiki/Monotremata) |
| XP_007936649.1 | *Orycteropus afer afer* | [Orycteropodidae](https://en.wikipedia.org/wiki/Orycteropodidae) | [Tubulidentata](https://en.wikipedia.org/wiki/Orycteropodidae) |
| XP_002721107.2 | *Oryctolagus cuniculus* | Leporidae | Lagomorpha |
| XP_012659258.1 | *Otolemur garnettii* | Galagidae | Primates |
| JAA27954.1 | *Pan troglodytes* | Hominidae | Primates |
| ACN22605.1 | *Panthera leo* | [Felidae](https://en.wikipedia.org/wiki/Felidae) | [Carnivora](https://en.wikipedia.org/wiki/Carnivora) |
| XP_015391029.1 | *Panthera tigris altaica* | Felidae | Carnivora |
| XP_005954767.1 | *Pantholops hodgsonii* | [Bovidae](https://en.wikipedia.org/wiki/Bovidae) | [Artiodactyla](https://en.wikipedia.org/wiki/Even-toed_ungulate) |
| XP_023083619.1 | *Piliocolobus tephrosceles* | [Cercopithecidae](https://en.wikipedia.org/wiki/Cercopithecidae) | [Primates](https://en.wikipedia.org/wiki/Primate) |
| ELK04219.1 | *Pteropus alecto* | Pteropodidae | Chiroptera |
| XP_023391820.1 | *Pteropus vampyrus* | Pteropodidae | Chiroptera |
| AAH88116.1 | *Rattus norvegicus* | [Muridae](https://en.wikipedia.org/wiki/Muridae) | [Rodentia](https://en.wikipedia.org/wiki/Rodent) |
| XP_010382572.1 | *Rhinopithecus roxellana* | [Cercopithecidae](https://en.wikipedia.org/wiki/Old_World_monkey) | [Primates](https://en.wikipedia.org/wiki/Primate) |
| XP_012395231.1 | *Sarcophilus harrisii* | Dasyuridae | Dasyuromorphia |
| XP_012789084.1 | *Sorex araneus* | Soricidae | Eulipotyphla |
| BAD93379.1 | *Suncus murinus* | [Soricidae](https://en.wikipedia.org/wiki/Soricidae) | [Eulipotyphla](https://en.wikipedia.org/wiki/Eulipotyphla) |
| JAG69746.1 | *Sus scrofa domesticus* | [Suidae](https://en.wikipedia.org/wiki/Suidae) | [Artiodactyla](https://en.wikipedia.org/wiki/Even-toed_ungulate) |
| XP_023598036.1 | *Trichechus manatus* | Trichechidae | Sirenia |
| ELW66767.1 | *Tupaia chinensis* | [Tupaiidae](https://en.wikipedia.org/wiki/Tupaiidae) | [Scandentia](https://en.wikipedia.org/wiki/Scandentia) |

**Supplementary Table S5. Primers Used to Amplify the Mutation Sites in *MHC* Class II Antigen *DRA*,** ***MHC* Class II Antigen *DRB*, and *ADA*.**

| **Primer name** | **Primer sequence (5'-3')** | | **Annealing temperature** | **Product length** | **Reference** |
| --- | --- | --- | --- | --- | --- |
|  | **Forward** | **Reverse** |  |  |  |
| MD-DRA | AGGGCTAAGCAGGGAAAG | GCGGAAGAGGTGGTCATT | 55℃ | 228 bp | This study |
| MD-DRB | GGTAGTTAGGGAGTCAGTGGAG | CTCTCACCAACCCCGTAGT | 55℃ | 406 bp | This study |
| MD-ADA | TCCCAGAGTTCCTGACCAA | AGGAGTTTCCACCCATCAC | 55℃ | 249 bp | This study |

**Supplementary Table S6. GO Enrichment Analysis of Significantly Expanded Gene Families in the FMD.**

| **GO ID** | **GO Term** | **GO Class** | **p-value** | **Adjusted p-value** | **No. of genes** |
| --- | --- | --- | --- | --- | --- |
| GO:0015074 | DNA integration | BP | 9.03E-64 | 4.87E-60 | 51 |
| GO:0004984 | olfactory receptor activity | MF | 7.21E-51 | 1.95E-47 | 152 |
| GO:0046718 | entry of virus into host cell | BP | 8.38E-42 | 1.13E-38 | 44 |
| GO:0019059 | initiation of viral infection | BP | 8.38E-42 | 1.13E-38 | 44 |
| GO:0007606 | sensory perception of chemical stimulus | BP | 1.78E-40 | 1.92E-37 | 119 |
| GO:0044409 | entry into host | BP | 3.77E-39 | 1.85E-36 | 44 |
| GO:0030260 | entry into host cell | BP | 3.77E-39 | 1.85E-36 | 44 |
| GO:0051806 | entry into cell of other organism involved in symbiotic interaction | BP | 3.77E-39 | 1.85E-36 | 44 |
| GO:0051828 | entry into other organism involved in symbiotic interaction | BP | 3.77E-39 | 1.85E-36 | 44 |
| GO:0052126 | movement in host environment | BP | 3.77E-39 | 1.85E-36 | 44 |
| GO:0052192 | movement in environment of other organism involved in symbiotic interaction | BP | 3.77E-39 | 1.85E-36 | 44 |
| GO:0007608 | sensory perception of smell | BP | 4.46E-36 | 2.00E-33 | 86 |
| GO:0004930 | G-protein coupled receptor activity | MF | 2.30E-33 | 9.56E-31 | 178 |
| GO:0000786 | nucleosome | CC | 8.68E-33 | 3.35E-30 | 45 |
| GO:0004888 | transmembrane signaling receptor activity | MF | 3.70E-32 | 1.33E-29 | 210 |
| GO:0016891 | endoribonuclease activity, producing 5'-phosphomonoesters | MF | 4.50E-30 | 1.52E-27 | 32 |
| GO:0007600 | sensory perception | BP | 2.85E-29 | 9.04E-27 | 149 |
| GO:0038023 | signaling receptor activity | MF | 1.90E-27 | 5.71E-25 | 210 |
| GO:0051701 | interaction with host | BP | 2.60E-26 | 7.40E-24 | 45 |
| GO:0050907 | detection of chemical stimulus involved in sensory perception | BP | 3.29E-26 | 8.88E-24 | 57 |
| GO:0050906 | detection of stimulus involved in sensory perception | BP | 6.79E-25 | 1.75E-22 | 63 |
| GO:0032993 | protein-DNA complex | CC | 7.36E-24 | 1.81E-21 | 50 |
| GO:0019058 | viral infectious cycle | BP | 2.65E-23 | 6.22E-21 | 68 |
| GO:0004523 | ribonuclease H activity | MF | 9.96E-23 | 2.24E-20 | 32 |
| GO:0044403 | symbiosis, encompassing mutualism through parasitism | BP | 1.98E-22 | 4.11E-20 | 49 |
| GO:0044419 | interspecies interaction between organisms | BP | 1.98E-22 | 4.11E-20 | 49 |
| GO:0009593 | detection of chemical stimulus | BP | 2.47E-22 | 4.93E-20 | 57 |
| GO:0004871 | signal transducer activity | MF | 7.55E-22 | 1.41E-19 | 224 |
| GO:0060089 | molecular transducer activity | MF | 7.55E-22 | 1.41E-19 | 224 |
| GO:0004521 | endoribonuclease activity | MF | 1.37E-21 | 2.47E-19 | 32 |
| GO:0004872 | receptor activity | MF | 4.95E-21 | 8.51E-19 | 214 |
| GO:0022415 | viral reproductive process | BP | 5.04E-21 | 8.51E-19 | 69 |
| GO:0050877 | neurological system process | BP | 4.20E-19 | 6.87E-17 | 211 |
| GO:0006334 | nucleosome assembly | BP | 4.56E-18 | 7.24E-16 | 40 |
| GO:0016893 | endonuclease activity, active with either ribo- or deoxyribonucleic acids and producing 5'-phosphomonoesters | MF | 2.18E-17 | 3.37E-15 | 42 |
| GO:0051606 | detection of stimulus | BP | 2.78E-17 | 4.16E-15 | 66 |
| GO:0031497 | chromatin assembly | BP | 1.21E-16 | 1.76E-14 | 41 |
| GO:0042393 | histone binding | MF | 1.46E-16 | 2.07E-14 | 46 |
| GO:0065004 | protein-DNA complex assembly | BP | 2.05E-15 | 2.84E-13 | 47 |
| GO:0004540 | ribonuclease activity | MF | 6.36E-15 | 8.58E-13 | 34 |
| GO:0034728 | nucleosome organization | BP | 2.33E-14 | 3.07E-12 | 40 |
| GO:0071824 | protein-DNA complex subunit organization | BP | 1.08E-13 | 1.39E-11 | 47 |
| GO:0003008 | system process | BP | 1.22E-13 | 1.51E-11 | 238 |
| GO:0006333 | chromatin assembly or disassembly | BP | 1.23E-13 | 1.51E-11 | 41 |
| GO:0016032 | viral reproduction | BP | 1.42E-13 | 1.70E-11 | 75 |
| GO:0051704 | multi-organism process | BP | 2.17E-13 | 2.55E-11 | 114 |
| GO:0007186 | G-protein coupled receptor signaling pathway | BP | 5.33E-13 | 6.12E-11 | 133 |
| GO:0007156 | homophilic cell adhesion | BP | 9.69E-13 | 1.09E-10 | 32 |
| GO:0005132 | interferon-alpha/beta receptor binding | MF | 1.30E-12 | 1.43E-10 | 14 |
| GO:0050911 | detection of chemical stimulus involved in sensory perception of smell | BP | 2.70E-12 | 2.92E-10 | 20 |
| GO:0000788 | nuclear nucleosome | CC | 3.14E-12 | 3.32E-10 | 19 |
| GO:0006323 | DNA packaging | BP | 3.53E-12 | 3.66E-10 | 42 |
| GO:0033141 | positive regulation of peptidyl-serine phosphorylation of STAT protein | BP | 1.12E-11 | 1.15E-09 | 14 |
| GO:0046982 | protein heterodimerization activity | MF | 1.44E-11 | 1.44E-09 | 66 |
| GO:0033139 | regulation of peptidyl-serine phosphorylation of STAT protein | BP | 2.88E-11 | 2.83E-09 | 14 |
| GO:0002227 | innate immune response in mucosa | BP | 3.98E-11 | 3.84E-09 | 12 |
| GO:0003823 | antigen binding | MF | 1.30E-10 | 1.24E-08 | 19 |
| GO:0031492 | nucleosomal DNA binding | MF | 2.07E-10 | 1.92E-08 | 19 |
| GO:0042501 | serine phosphorylation of STAT protein | BP | 3.24E-10 | 2.97E-08 | 14 |
| GO:0006826 | iron ion transport | BP | 3.77E-10 | 3.40E-08 | 20 |
| GO:0002323 | natural killer cell activation involved in immune response | BP | 6.54E-10 | 5.79E-08 | 14 |
| GO:0071103 | DNA conformation change | BP | 8.80E-10 | 7.66E-08 | 44 |
| GO:0022414 | reproductive process | BP | 1.08E-09 | 9.26E-08 | 148 |
| GO:0006959 | humoral immune response | BP | 1.10E-09 | 9.26E-08 | 29 |
| GO:0000003 | reproduction | BP | 1.32E-09 | 1.10E-07 | 148 |
| GO:0071844 | cellular component assembly at cellular level | BP | 2.53E-09 | 2.07E-07 | 179 |
| GO:0043331 | response to dsRNA | BP | 6.09E-09 | 4.76E-07 | 18 |
| GO:0034622 | cellular macromolecular complex assembly | BP | 8.48E-09 | 6.54E-07 | 111 |
| GO:0002385 | mucosal immune response | BP | 8.89E-09 | 6.76E-07 | 13 |
| GO:0019013 | viral nucleocapsid | CC | 9.26E-09 | 6.76E-07 | 12 |
| GO:0006335 | DNA replication-dependent nucleosome assembly | BP | 9.26E-09 | 6.76E-07 | 12 |
| GO:0034723 | DNA replication-dependent nucleosome organization | BP | 9.26E-09 | 6.76E-07 | 12 |
| GO:0017154 | semaphorin receptor activity | MF | 1.27E-08 | 9.01E-07 | 9 |
| GO:0004035 | alkaline phosphatase activity | MF | 1.28E-08 | 9.01E-07 | 7 |
| GO:0005200 | structural constituent of cytoskeleton | MF | 1.54E-08 | 1.04E-06 | 21 |
| GO:0002251 | organ or tissue specific immune response | BP | 1.60E-08 | 1.06E-06 | 13 |
| GO:0000785 | chromatin | CC | 2.00E-08 | 1.30E-06 | 67 |
| GO:0019012 | virion | CC | 2.78E-08 | 1.78E-06 | 13 |
| GO:0034621 | cellular macromolecular complex subunit organization | BP | 3.59E-08 | 2.28E-06 | 120 |
| GO:0003887 | DNA-directed DNA polymerase activity | MF | 5.90E-08 | 3.66E-06 | 17 |
| GO:0020002 | host cell plasma membrane | CC | 6.03E-08 | 3.66E-06 | 12 |
| GO:0019028 | viral capsid | CC | 6.03E-08 | 3.66E-06 | 12 |
| GO:0044423 | virion part | CC | 6.03E-08 | 3.66E-06 | 12 |
| GO:0031491 | nucleosome binding | MF | 6.71E-08 | 4.02E-06 | 19 |
| GO:0071944 | cell periphery | CC | 7.70E-08 | 4.52E-06 | 459 |
| GO:0022607 | cellular component assembly | BP | 9.06E-08 | 5.21E-06 | 214 |
| GO:0033644 | host cell membrane | CC | 1.04E-07 | 5.93E-06 | 12 |
| GO:0005886 | plasma membrane | CC | 1.64E-07 | 9.21E-06 | 448 |
| GO:0044218 | other organism cell membrane | CC | 1.75E-07 | 9.64E-06 | 12 |
| GO:0044279 | other organism membrane | CC | 1.75E-07 | 9.64E-06 | 12 |
| GO:0043330 | response to exogenous dsRNA | BP | 1.89E-07 | 1.03E-05 | 14 |
| GO:0019067 | viral assembly, maturation, egress, and release | BP | 4.05E-07 | 2.18E-05 | 14 |
| GO:0016779 | nucleotidyltransferase activity | MF | 5.21E-07 | 2.76E-05 | 85 |
| GO:0005549 | odorant binding | MF | 5.54E-07 | 2.90E-05 | 27 |
| GO:0019076 | release of virus from host | BP | 6.53E-07 | 3.39E-05 | 11 |
| GO:0044085 | cellular component biogenesis | BP | 8.74E-07 | 4.49E-05 | 232 |
| GO:0065003 | macromolecular complex assembly | BP | 8.81E-07 | 4.49E-05 | 153 |
| GO:0051966 | regulation of synaptic transmission, glutamatergic | BP | 8.99E-07 | 4.54E-05 | 15 |
| GO:0019731 | antibacterial humoral response | BP | 1.07E-06 | 5.31E-05 | 12 |
| GO:0033643 | host cell part | CC | 1.40E-06 | 6.80E-05 | 13 |
| GO:0033138 | positive regulation of peptidyl-serine phosphorylation | BP | 1.46E-06 | 6.98E-05 | 17 |
| GO:0007216 | G-protein coupled glutamate receptor signaling pathway | BP | 1.48E-06 | 6.98E-05 | 10 |
| GO:0046961 | proton-transporting ATPase activity, rotational mechanism | MF | 1.48E-06 | 6.98E-05 | 10 |
| GO:0051287 | NAD binding | MF | 1.49E-06 | 6.98E-05 | 16 |
| GO:0043933 | macromolecular complex subunit organization | BP | 2.33E-06 | 0.000105866 | 162 |
| GO:0030529 | ribonucleoprotein complex | CC | 2.89E-06 | 0.000127731 | 109 |
| GO:0035249 | synaptic transmission, glutamatergic | BP | 3.03E-06 | 0.000132893 | 17 |
| GO:0031867 | EP4 subtype prostaglandin E2 receptor binding | MF | 3.24E-06 | 0.000141039 | 7 |
| GO:0035098 | ESC/E(Z) complex | CC | 3.29E-06 | 0.000142167 | 9 |
| GO:0018995 | host | CC | 3.82E-06 | 0.00016235 | 13 |
| GO:0043657 | host cell | CC | 3.82E-06 | 0.00016235 | 13 |
| GO:0033135 | regulation of peptidyl-serine phosphorylation | BP | 5.34E-06 | 0.000225191 | 18 |
| GO:0002286 | T cell activation involved in immune response | BP | 7.03E-06 | 0.000283388 | 16 |
| GO:0043245 | extraorganismal space | CC | 7.03E-06 | 0.000283388 | 13 |
| GO:0044215 | other organism | CC | 7.03E-06 | 0.000283388 | 13 |
| GO:0044216 | other organism cell | CC | 7.03E-06 | 0.000283388 | 13 |
| GO:0044217 | other organism part | CC | 7.03E-06 | 0.000283388 | 13 |
| GO:0031862 | prostanoid receptor binding | MF | 7.27E-06 | 0.000288625 | 7 |
| GO:0004190 | aspartic-type endopeptidase activity | MF | 1.06E-05 | 0.000416596 | 24 |
| GO:0016337 | cell-cell adhesion | BP | 1.11E-05 | 0.000433425 | 55 |
| GO:0044454 | nuclear chromosome part | CC | 1.13E-05 | 0.000437374 | 56 |
| GO:0070001 | aspartic-type peptidase activity | MF | 1.27E-05 | 0.000488708 | 24 |
| GO:0052593 | tryptamine:oxygen oxidoreductase (deaminating) activity | MF | 1.30E-05 | 0.000488708 | 5 |
| GO:0052596 | phenethylamine:oxygen oxidoreductase (deaminating) activity | MF | 1.30E-05 | 0.000488708 | 5 |
| GO:0052595 | aliphatic-amine oxidase activity | MF | 1.30E-05 | 0.000488708 | 5 |
| GO:0052594 | aminoacetone:oxygen oxidoreductase(deaminating) activity | MF | 1.30E-05 | 0.000488708 | 5 |
| GO:0003676 | nucleic acid binding | MF | 1.57E-05 | 0.00057985 | 356 |
| GO:0006412 | translation | BP | 2.01E-05 | 0.000722187 | 84 |
| GO:0048841 | regulation of axon extension involved in axon guidance | BP | 2.12E-05 | 0.000756586 | 8 |
| GO:0016071 | mRNA metabolic process | BP | 2.17E-05 | 0.000771863 | 78 |
| GO:0000790 | nuclear chromatin | CC | 2.35E-05 | 0.000829725 | 40 |
| GO:0007196 | inhibition of adenylate cyclase activity by G-protein coupled glutamate receptor signaling pathway | BP | 2.76E-05 | 0.000945612 | 7 |
| GO:2001241 | positive regulation of extrinsic apoptotic signaling pathway in absence of ligand | BP | 2.76E-05 | 0.000945612 | 7 |
| GO:0000041 | transition metal ion transport | BP | 2.77E-05 | 0.000945612 | 22 |
| GO:0008131 | primary amine oxidase activity | MF | 2.78E-05 | 0.000945612 | 6 |
| GO:0014047 | glutamate secretion | BP | 2.82E-05 | 0.000952297 | 10 |
| GO:0008066 | glutamate receptor activity | MF | 2.98E-05 | 0.000991565 | 12 |
| GO:0046425 | regulation of JAK-STAT cascade | BP | 3.04E-05 | 0.001007143 | 18 |
| GO:0097114 | N-methyl-D-aspartate receptor clustering | BP | 3.11E-05 | 0.001016789 | 4 |
| GO:0051232 | meiotic spindle elongation | BP | 3.11E-05 | 0.001016789 | 4 |
| GO:0044427 | chromosomal part | CC | 3.56E-05 | 0.001158324 | 90 |
| GO:0022625 | cytosolic large ribosomal subunit | CC | 3.60E-05 | 0.001163216 | 28 |
| GO:0019730 | antimicrobial humoral response | BP | 3.88E-05 | 0.001248198 | 12 |
| GO:0000398 | nuclear mRNA splicing, via spliceosome | BP | 4.18E-05 | 0.001328153 | 39 |
| GO:0000377 | RNA splicing, via transesterification reactions with bulged adenosine as nucleophile | BP | 4.18E-05 | 0.001328153 | 39 |
| GO:0000220 | vacuolar proton-transporting V-type ATPase, V0 domain | CC | 4.28E-05 | 0.001351156 | 5 |
| GO:0000375 | RNA splicing, via transesterification reactions | BP | 4.65E-05 | 0.001458402 | 39 |
| GO:0016458 | gene silencing | BP | 5.63E-05 | 0.001756312 | 29 |
| GO:0046983 | protein dimerization activity | MF | 5.80E-05 | 0.00179865 | 111 |
| GO:0048167 | regulation of synaptic plasticity | BP | 5.87E-05 | 0.001812274 | 18 |
| GO:0031490 | chromatin DNA binding | MF | 6.04E-05 | 0.001851741 | 19 |
| GO:0042734 | presynaptic membrane | CC | 6.33E-05 | 0.001931541 | 16 |
| GO:0008199 | ferric iron binding | MF | 6.97E-05 | 0.002102423 | 13 |
| GO:0006325 | chromatin organization | BP | 7.01E-05 | 0.002103812 | 73 |
| GO:0071526 | semaphorin-plexin signaling pathway | BP | 8.04E-05 | 0.002371722 | 7 |
| GO:0009152 | purine ribonucleotide biosynthetic process | BP | 9.09E-05 | 0.002645084 | 15 |
| GO:0034061 | DNA polymerase activity | MF | 9.11E-05 | 0.002645084 | 69 |
| GO:0007157 | heterophilic cell-cell adhesion | BP | 9.27E-05 | 0.002660801 | 11 |
| GO:0007215 | glutamate receptor signaling pathway | BP | 9.27E-05 | 0.002660801 | 11 |
| GO:0051290 | protein heterotetramerization | BP | 9.65E-05 | 0.002757889 | 9 |
| GO:0015078 | hydrogen ion transmembrane transporter activity | MF | 0.000101 | 0.002841659 | 16 |
| GO:0051705 | behavioral interaction between organisms | BP | 0.000101 | 0.002841659 | 14 |
| GO:0000228 | nuclear chromosome | CC | 0.000105 | 0.002841659 | 56 |
| GO:0030101 | natural killer cell activation | BP | 0.000105 | 0.002841659 | 14 |
| GO:0021785 | branchiomotor neuron axon guidance | BP | 0.000107 | 0.002841659 | 5 |
| GO:0008559 | xenobiotic-transporting ATPase activity | MF | 0.000107 | 0.002841659 | 5 |
| GO:0072494 | host multivesicular body | CC | 0.000107 | 0.002841659 | 5 |
| GO:0044185 | host cell late endosome membrane | CC | 0.000107 | 0.002841659 | 5 |
| GO:0042910 | xenobiotic transporter activity | MF | 0.000107 | 0.002841659 | 5 |
| GO:0044174 | host cell endosome | CC | 0.000107 | 0.002841659 | 5 |
| GO:0044184 | host cell late endosome | CC | 0.000107 | 0.002841659 | 5 |
| GO:0044175 | host cell endosome membrane | CC | 0.000107 | 0.002841659 | 5 |
| GO:0043931 | ossification involved in bone maturation | BP | 0.000107 | 0.002841659 | 6 |
| GO:0007506 | gonadal mesoderm development | BP | 0.000107 | 0.002841659 | 6 |
| GO:0035459 | cargo loading into vesicle | BP | 0.000107 | 0.002841659 | 6 |
| GO:0007416 | synapse assembly | BP | 0.000125 | 0.003261637 | 20 |
| GO:0042287 | MHC protein binding | MF | 0.000128 | 0.003317213 | 7 |
| GO:0003723 | RNA binding | MF | 0.000128 | 0.003317213 | 165 |
| GO:0015934 | large ribosomal subunit | CC | 0.000145 | 0.003537355 | 32 |
| GO:0072137 | condensed mesenchymal cell proliferation | BP | 0.000146 | 0.003537355 | 4 |
| GO:0033300 | dehydroascorbic acid transporter activity | MF | 0.000146 | 0.003537355 | 4 |
| GO:0097119 | postsynaptic density protein 95 clustering | BP | 0.000146 | 0.003537355 | 4 |
| GO:0044305 | calyx of Held | CC | 0.000146 | 0.003537355 | 4 |
| GO:0030160 | GKAP/Homer scaffold activity | MF | 0.000146 | 0.003537355 | 4 |
| GO:2000969 | positive regulation of alpha-amino-3-hydroxy-5-methyl-4-isoxazole propionate selective glutamate receptor activity | BP | 0.000146 | 0.003537355 | 4 |
| GO:0005587 | collagen type IV | CC | 0.000146 | 0.003537355 | 4 |
| GO:0004698 | calcium-dependent protein kinase C activity | MF | 0.000146 | 0.003537355 | 4 |
| GO:0030935 | sheet-forming collagen | CC | 0.000146 | 0.003537355 | 4 |
| GO:0043484 | regulation of RNA splicing | BP | 0.000153 | 0.003679859 | 22 |
| GO:0005198 | structural molecule activity | MF | 0.000154 | 0.003696351 | 86 |
| GO:0035064 | methylated histone residue binding | MF | 0.000166 | 0.003974956 | 14 |
| GO:0055072 | iron ion homeostasis | BP | 0.000185 | 0.004373038 | 19 |
| GO:0070977 | bone maturation | BP | 0.000187 | 0.004389416 | 6 |
| GO:0035176 | social behavior | BP | 0.000192 | 0.004492528 | 11 |
| GO:0043492 | ATPase activity, coupled to movement of substances | MF | 0.000193 | 0.004501497 | 21 |
| GO:2000311 | regulation of alpha-amino-3-hydroxy-5-methyl-4-isoxazole propionate selective glutamate receptor activity | BP | 0.000196 | 0.004535952 | 7 |
| GO:0042100 | B cell proliferation | BP | 0.000219 | 0.005022859 | 14 |
| GO:0003964 | RNA-directed DNA polymerase activity | MF | 0.00022 | 0.005022859 | 65 |
| GO:0070072 | vacuolar proton-transporting V-type ATPase complex assembly | BP | 0.000226 | 0.005063392 | 5 |
| GO:0035605 | peptidyl-cysteine S-nitrosylase activity | MF | 0.000226 | 0.005063392 | 5 |
| GO:0035606 | peptidyl-cysteine S-trans-nitrosylation | BP | 0.000226 | 0.005063392 | 5 |
| GO:0070070 | proton-transporting V-type ATPase complex assembly | BP | 0.000226 | 0.005063392 | 5 |
| GO:0072578 | neurotransmitter-gated ion channel clustering | BP | 0.000226 | 0.005063392 | 5 |
| GO:0004322 | ferroxidase activity | MF | 0.000237 | 0.00524667 | 8 |
| GO:0022829 | wide pore channel activity | MF | 0.000237 | 0.00524667 | 8 |
| GO:0016724 | oxidoreductase activity, oxidizing metal ions, oxygen as acceptor | MF | 0.000237 | 0.00524667 | 8 |
| GO:0005246 | calcium channel regulator activity | MF | 0.00024 | 0.005293197 | 9 |
| GO:0005694 | chromosome | CC | 0.000251 | 0.005458748 | 98 |
| GO:0006163 | purine nucleotide metabolic process | BP | 0.000251 | 0.005458748 | 63 |
| GO:0006836 | neurotransmitter transport | BP | 0.000257 | 0.005556564 | 24 |
| GO:0043487 | regulation of RNA stability | BP | 0.000288 | 0.006171087 | 16 |
| GO:0055029 | nuclear DNA-directed RNA polymerase complex | CC | 0.0003 | 0.00639961 | 11 |
| GO:0003735 | structural constituent of ribosome | MF | 0.000317 | 0.0067357 | 45 |
| GO:0048846 | axon extension involved in axon guidance | BP | 0.000326 | 0.006902161 | 8 |
| GO:0019829 | cation-transporting ATPase activity | MF | 0.000358 | 0.007502039 | 10 |
| GO:0000428 | DNA-directed RNA polymerase complex | CC | 0.00037 | 0.007662225 | 11 |
| GO:0030880 | RNA polymerase complex | CC | 0.00037 | 0.007662225 | 11 |
| GO:0007269 | neurotransmitter secretion | BP | 0.000391 | 0.007909773 | 20 |
| GO:0050830 | defense response to Gram-positive bacterium | BP | 0.000403 | 0.007909773 | 13 |
| GO:0002116 | semaphorin receptor complex | CC | 0.000412 | 0.007909773 | 4 |
| GO:0008381 | mechanically-gated ion channel activity | MF | 0.000412 | 0.007909773 | 4 |
| GO:0055056 | D-glucose transmembrane transporter activity | MF | 0.000412 | 0.007909773 | 4 |
| GO:0005030 | neurotrophin receptor activity | MF | 0.000412 | 0.007909773 | 4 |
| GO:0006933 | negative regulation of cell adhesion involved in substrate-bound cell migration | BP | 0.000412 | 0.007909773 | 4 |
| GO:0022833 | mechanically gated channel activity | MF | 0.000412 | 0.007909773 | 4 |
| GO:0051231 | spindle elongation | BP | 0.000412 | 0.007909773 | 4 |
| GO:0015086 | cadmium ion transmembrane transporter activity | MF | 0.000417 | 0.007909773 | 3 |
| GO:0015087 | cobalt ion transmembrane transporter activity | MF | 0.000417 | 0.007909773 | 3 |
| GO:0015639 | ferrous iron uptake transmembrane transporter activity | MF | 0.000417 | 0.007909773 | 3 |
| GO:0015099 | nickel cation transmembrane transporter activity | MF | 0.000417 | 0.007909773 | 3 |
| GO:0015094 | lead ion transmembrane transporter activity | MF | 0.000417 | 0.007909773 | 3 |
| GO:0015684 | ferrous iron transport | BP | 0.000417 | 0.007909773 | 3 |
| GO:0004450 | isocitrate dehydrogenase (NADP+) activity | MF | 0.000417 | 0.007909773 | 3 |
| GO:0006097 | glyoxylate cycle | BP | 0.000417 | 0.007909773 | 3 |
| GO:0018444 | translation release factor complex | CC | 0.000417 | 0.007909773 | 3 |
| GO:0002184 | cytoplasmic translational termination | BP | 0.000417 | 0.007909773 | 3 |
| GO:0047442 | 17-alpha-hydroxyprogesterone aldolase activity | MF | 0.000417 | 0.007909773 | 3 |
| GO:0004508 | steroid 17-alpha-monooxygenase activity | MF | 0.000417 | 0.007909773 | 3 |
| GO:0045332 | phospholipid translocation | BP | 0.000418 | 0.007909773 | 7 |
| GO:0051968 | positive regulation of synaptic transmission, glutamatergic | BP | 0.000424 | 0.007952092 | 5 |
| GO:0005384 | manganese ion transmembrane transporter activity | MF | 0.000424 | 0.007952092 | 5 |
| GO:0021559 | trigeminal nerve development | BP | 0.000424 | 0.007952092 | 5 |
| GO:0007270 | neuron-neuron synaptic transmission | BP | 0.000426 | 0.007958821 | 19 |
| GO:0001941 | postsynaptic membrane organization | BP | 0.00044 | 0.008112415 | 8 |
| GO:0042953 | lipoprotein transport | BP | 0.000479 | 0.008673104 | 6 |
| GO:0033647 | host intracellular organelle | CC | 0.000479 | 0.008673104 | 6 |
| GO:0033648 | host intracellular membrane-bounded organelle | CC | 0.000479 | 0.008673104 | 6 |
| GO:0043488 | regulation of mRNA stability | BP | 0.000488 | 0.00878324 | 13 |
| GO:0006396 | RNA processing | BP | 0.000508 | 0.009076975 | 97 |
| GO:0007194 | negative regulation of adenylate cyclase activity | BP | 0.000513 | 0.009118854 | 14 |
| GO:0032991 | macromolecular complex | CC | 0.000546 | 0.009638451 | 408 |
| GO:0022626 | cytosolic ribosome | CC | 0.000554 | 0.009739496 | 36 |
| GO:0042162 | telomeric DNA binding | MF | 0.00056 | 0.00982475 | 10 |
| GO:0009150 | purine ribonucleotide metabolic process | BP | 0.000563 | 0.009840205 | 38 |
| GO:0034204 | lipid translocation | BP | 0.000586 | 0.010175551 | 7 |
| GO:0045814 | negative regulation of gene expression, epigenetic | BP | 0.000628 | 0.010764069 | 15 |
| GO:0031280 | negative regulation of cyclase activity | BP | 0.000631 | 0.010772674 | 14 |
| GO:0030490 | maturation of SSU-rRNA | BP | 0.000662 | 0.011271363 | 13 |
| GO:0033119 | negative regulation of RNA splicing | BP | 0.000671 | 0.011391817 | 9 |
| GO:0030430 | host cell cytoplasm | CC | 0.000718 | 0.012071405 | 6 |
| GO:0031223 | auditory behavior | BP | 0.00073 | 0.012199838 | 5 |
| GO:0033179 | proton-transporting V-type ATPase, V0 domain | CC | 0.00073 | 0.012199838 | 5 |
| GO:0030516 | regulation of axon extension | BP | 0.000757 | 0.012529768 | 13 |
| GO:0015991 | ATP hydrolysis coupled proton transport | BP | 0.000766 | 0.012567193 | 8 |
| GO:0015988 | energy coupled proton transport, against electrochemical gradient | BP | 0.000766 | 0.012567193 | 8 |
| GO:0051350 | negative regulation of lyase activity | BP | 0.00077 | 0.01259112 | 14 |
| GO:0046034 | ATP metabolic process | BP | 0.000775 | 0.012634122 | 13 |
| GO:0007166 | cell surface receptor signaling pathway | BP | 0.000787 | 0.012719411 | 243 |
| GO:0008380 | RNA splicing | BP | 0.000794 | 0.012778382 | 49 |
| GO:0006405 | RNA export from nucleus | BP | 0.000803 | 0.012871908 | 17 |
| GO:0071695 | anatomical structure maturation | BP | 0.000844 | 0.013398517 | 9 |
| GO:0042623 | ATPase activity, coupled | MF | 0.000846 | 0.013398517 | 36 |
| GO:0050051 | leukotriene-B4 20-monooxygenase activity | MF | 0.000905 | 0.013872946 | 4 |
| GO:0003095 | pressure natriuresis | BP | 0.000905 | 0.013872946 | 4 |
| GO:0018685 | alkane 1-monooxygenase activity | MF | 0.000905 | 0.013872946 | 4 |
| GO:0051754 | meiotic sister chromatid cohesion, centromeric | BP | 0.000905 | 0.013872946 | 4 |
| GO:0034227 | tRNA thio-modification | BP | 0.000905 | 0.013872946 | 4 |
| GO:0015266 | protein channel activity | MF | 0.000905 | 0.013872946 | 4 |
| GO:0001640 | adenylate cyclase inhibiting G-protein coupled glutamate receptor activity | MF | 0.000905 | 0.013872946 | 4 |
| GO:0016427 | tRNA (cytosine) methyltransferase activity | MF | 0.000905 | 0.013872946 | 4 |
| GO:0044391 | ribosomal subunit | CC | 0.000939 | 0.014316015 | 42 |
| GO:0003674 | molecular_function | MF | 0.001028 | 0.015333301 | 1201 |
| GO:0000049 | tRNA binding | MF | 0.001029 | 0.015333301 | 12 |
| GO:0004402 | histone acetyltransferase activity | MF | 0.001029 | 0.015333301 | 12 |
| GO:0048786 | presynaptic active zone | CC | 0.001033 | 0.015333301 | 10 |
| GO:0005681 | spliceosomal complex | CC | 0.001036 | 0.015333301 | 28 |
| GO:0048038 | quinone binding | MF | 0.001039 | 0.015333301 | 6 |
| GO:0048172 | regulation of short-term neuronal synaptic plasticity | BP | 0.001039 | 0.015333301 | 6 |
| GO:0046040 | IMP metabolic process | BP | 0.001039 | 0.015333301 | 6 |
| GO:0070971 | endoplasmic reticulum exit site | CC | 0.001039 | 0.015333301 | 6 |
| GO:0045494 | photoreceptor cell maintenance | BP | 0.001052 | 0.01547102 | 9 |
| GO:0016021 | integral to membrane | CC | 0.001079 | 0.015784985 | 443 |
| GO:0050808 | synapse organization | BP | 0.001095 | 0.015975428 | 27 |
| GO:0030818 | negative regulation of cAMP biosynthetic process | BP | 0.001127 | 0.016356267 | 14 |
| GO:0009142 | nucleoside triphosphate biosynthetic process | BP | 0.001143 | 0.016542547 | 13 |
| GO:0007035 | vacuolar acidification | BP | 0.001174 | 0.016863583 | 5 |
| GO:0004365 | glyceraldehyde-3-phosphate dehydrogenase (NAD+) (phosphorylating) activity | MF | 0.001174 | 0.016863583 | 5 |
| GO:0070071 | proton-transporting two-sector ATPase complex assembly | BP | 0.001174 | 0.016863583 | 5 |
| GO:0006397 | mRNA processing | BP | 0.00122 | 0.017473028 | 57 |
| GO:0009260 | ribonucleotide biosynthetic process | BP | 0.001284 | 0.018293816 | 15 |
| GO:0030803 | negative regulation of cyclic nucleotide biosynthetic process | BP | 0.001353 | 0.019064613 | 14 |
| GO:0030815 | negative regulation of cAMP metabolic process | BP | 0.001353 | 0.019064613 | 14 |
| GO:0000380 | alternative nuclear mRNA splicing, via spliceosome | BP | 0.001361 | 0.019081719 | 11 |
| GO:0000027 | ribosomal large subunit assembly | BP | 0.00139 | 0.01939797 | 13 |
| GO:0006342 | chromatin silencing | BP | 0.00139 | 0.01939797 | 13 |
| GO:0015986 | ATP synthesis coupled proton transport | BP | 0.001431 | 0.019757186 | 7 |
| GO:0004993 | serotonin receptor activity | MF | 0.001431 | 0.019757186 | 7 |
| GO:0015985 | energy coupled proton transport, down electrochemical gradient | BP | 0.001431 | 0.019757186 | 7 |
| GO:0033646 | host intracellular part | CC | 0.001461 | 0.020075175 | 6 |
| GO:0043656 | intracellular region of host | CC | 0.001461 | 0.020075175 | 6 |
| GO:0048168 | regulation of neuronal synaptic plasticity | BP | 0.001499 | 0.020369089 | 10 |
| GO:0050684 | regulation of mRNA processing | BP | 0.001536 | 0.020369089 | 18 |
| GO:0007206 | activation of phospholipase C activity by G-protein coupled glutamate receptor signaling pathway | BP | 0.001573 | 0.020369089 | 3 |
| GO:0047484 | regulation of response to osmotic stress | BP | 0.001573 | 0.020369089 | 3 |
| GO:0051835 | positive regulation of synapse structural plasticity | BP | 0.001573 | 0.020369089 | 3 |
| GO:0006102 | isocitrate metabolic process | BP | 0.001573 | 0.020369089 | 3 |
| GO:0004367 | glycerol-3-phosphate dehydrogenase [NAD+] activity | MF | 0.001573 | 0.020369089 | 3 |
| GO:0046168 | glycerol-3-phosphate catabolic process | BP | 0.001573 | 0.020369089 | 3 |
| GO:0004119 | cGMP-inhibited cyclic-nucleotide phosphodiesterase activity | MF | 0.001573 | 0.020369089 | 3 |
| GO:0016309 | 1-phosphatidylinositol-5-phosphate 4-kinase activity | MF | 0.001573 | 0.020369089 | 3 |
| GO:0004019 | adenylosuccinate synthase activity | MF | 0.001573 | 0.020369089 | 3 |
| GO:0004422 | hypoxanthine phosphoribosyltransferase activity | MF | 0.001573 | 0.020369089 | 3 |
| GO:0052657 | guanine phosphoribosyltransferase activity | MF | 0.001573 | 0.020369089 | 3 |
| GO:0006178 | guanine salvage | BP | 0.001573 | 0.020369089 | 3 |
| GO:0000818 | nuclear MIS12/MIND type complex | CC | 0.001573 | 0.020369089 | 3 |
| GO:0070326 | very-low-density lipoprotein particle receptor binding | MF | 0.001573 | 0.020369089 | 3 |
| GO:0070973 | protein localization to endoplasmic reticulum exit site | BP | 0.001573 | 0.020369089 | 3 |
| GO:0035403 | histone kinase activity (H3-T6 specific) | MF | 0.001573 | 0.020369089 | 3 |
| GO:0035408 | histone H3-T6 phosphorylation | BP | 0.001573 | 0.020369089 | 3 |
| GO:0000746 | conjugation | BP | 0.001573 | 0.020369089 | 3 |
| GO:0046099 | guanine biosynthetic process | BP | 0.001573 | 0.020369089 | 3 |
| GO:0032391 | photoreceptor connecting cilium | CC | 0.001591 | 0.020543897 | 9 |
| GO:0043954 | cellular component maintenance | BP | 0.001603 | 0.02065626 | 11 |
| GO:0030800 | negative regulation of cyclic nucleotide metabolic process | BP | 0.001615 | 0.020712387 | 14 |
| GO:0008080 | N-acetyltransferase activity | MF | 0.001615 | 0.020712387 | 14 |
| GO:0045296 | cadherin binding | MF | 0.001622 | 0.020754573 | 32 |
| GO:0007155 | cell adhesion | BP | 0.001681 | 0.021378248 | 97 |
| GO:0019992 | diacylglycerol binding | MF | 0.001703 | 0.021378248 | 4 |
| GO:0070262 | peptidyl-serine dephosphorylation | BP | 0.001703 | 0.021378248 | 4 |
| GO:0033186 | CAF-1 complex | CC | 0.001703 | 0.021378248 | 4 |
| GO:0055077 | gap junction hemi-channel activity | MF | 0.001703 | 0.021378248 | 4 |
| GO:0021604 | cranial nerve structural organization | BP | 0.001703 | 0.021378248 | 4 |
| GO:2000194 | regulation of female gonad development | BP | 0.001703 | 0.021378248 | 4 |
| GO:0022610 | biological adhesion | BP | 0.001745 | 0.021760749 | 97 |
| GO:0006164 | purine nucleotide biosynthetic process | BP | 0.001755 | 0.02183202 | 31 |
| GO:0060045 | positive regulation of cardiac muscle cell proliferation | BP | 0.001792 | 0.022185024 | 5 |
| GO:0007638 | mechanosensory behavior | BP | 0.001792 | 0.022185024 | 5 |
| GO:0050839 | cell adhesion molecule binding | MF | 0.001831 | 0.022574063 | 37 |
| GO:0030809 | negative regulation of nucleotide biosynthetic process | BP | 0.001919 | 0.023545381 | 14 |
| GO:0000381 | regulation of alternative nuclear mRNA splicing, via spliceosome | BP | 0.001933 | 0.023614539 | 9 |
| GO:0009259 | ribonucleotide metabolic process | BP | 0.001933 | 0.023614539 | 38 |
| GO:0060043 | regulation of cardiac muscle cell proliferation | BP | 0.002002 | 0.024233602 | 6 |
| GO:0007193 | inhibition of adenylate cyclase activity by G-protein signaling pathway | BP | 0.002179 | 0.026256683 | 12 |
| GO:0006353 | transcription termination, DNA-dependent | BP | 0.002194 | 0.026378184 | 11 |
| GO:0048024 | regulation of nuclear mRNA splicing, via spliceosome | BP | 0.002269 | 0.027220799 | 14 |
| GO:0006970 | response to osmotic stress | BP | 0.002331 | 0.027727815 | 9 |
| GO:0031519 | PcG protein complex | CC | 0.002331 | 0.027727815 | 9 |
| GO:0006259 | DNA metabolic process | BP | 0.002371 | 0.027977786 | 129 |
| GO:0032232 | negative regulation of actin filament bundle assembly | BP | 0.002384 | 0.027977786 | 7 |
| GO:2001238 | positive regulation of extrinsic apoptotic signaling pathway | BP | 0.002384 | 0.027977786 | 7 |
| GO:0016638 | oxidoreductase activity, acting on the CH-NH2 group of donors | MF | 0.002384 | 0.027977786 | 7 |
| GO:0042274 | ribosomal small subunit biogenesis | BP | 0.002444 | 0.028511134 | 15 |
| GO:0003899 | DNA-directed RNA polymerase activity | MF | 0.002505 | 0.029023147 | 10 |
| GO:0016469 | proton-transporting two-sector ATPase complex | CC | 0.002505 | 0.029023147 | 10 |
| GO:0034062 | RNA polymerase activity | MF | 0.002505 | 0.029023147 | 10 |
| GO:0015992 | proton transport | BP | 0.002513 | 0.029049114 | 12 |
| GO:0016410 | N-acyltransferase activity | MF | 0.002553 | 0.029451143 | 16 |
| GO:0042626 | ATPase activity, coupled to transmembrane movement of substances | MF | 0.002606 | 0.029866498 | 17 |
| GO:0071625 | vocalization behavior | BP | 0.002617 | 0.029866498 | 5 |
| GO:0033655 | host cell cytoplasm part | CC | 0.002617 | 0.029866498 | 5 |
| GO:0045980 | negative regulation of nucleotide metabolic process | BP | 0.00267 | 0.030414025 | 14 |
| GO:0016641 | oxidoreductase activity, acting on the CH-NH2 group of donors, oxygen as acceptor | MF | 0.002681 | 0.030471883 | 6 |
| GO:0006754 | ATP biosynthetic process | BP | 0.002792 | 0.031499429 | 9 |
| GO:0042625 | ATPase activity, coupled to transmembrane movement of ions | MF | 0.002865 | 0.03210421 | 12 |
| GO:0004716 | receptor signaling protein tyrosine kinase activity | MF | 0.002884 | 0.03210421 | 4 |
| GO:0000389 | nuclear mRNA 3'-splice site recognition | BP | 0.002884 | 0.03210421 | 4 |
| GO:0016713 | oxidoreductase activity, acting on paired donors, with incorporation or reduction of molecular oxygen, reduced iron-sulfur protein as one donor, and incorporation of one atom of oxygen | MF | 0.002884 | 0.03210421 | 4 |
| GO:0005678 | chromatin assembly complex | CC | 0.002884 | 0.03210421 | 4 |
| GO:0004519 | endonuclease activity | MF | 0.002933 | 0.032586826 | 65 |
| GO:0007162 | negative regulation of cell adhesion | BP | 0.002966 | 0.032876931 | 18 |
| GO:0016722 | oxidoreductase activity, oxidizing metal ions | MF | 0.002996 | 0.033140925 | 8 |
| GO:0051276 | chromosome organization | BP | 0.003276 | 0.036094611 | 91 |
| GO:0048675 | axon extension | BP | 0.00329 | 0.036180711 | 15 |
| GO:0016820 | hydrolase activity, acting on acid anhydrides, catalyzing transmembrane movement of substances | MF | 0.00339 | 0.037198335 | 17 |
| GO:0019373 | epoxygenase P450 pathway | BP | 0.003519 | 0.038302346 | 6 |
| GO:0008392 | arachidonic acid epoxygenase activity | MF | 0.003519 | 0.038302346 | 6 |
| GO:0040011 | locomotion | BP | 0.003564 | 0.038635699 | 127 |
| GO:0006818 | hydrogen transport | BP | 0.003622 | 0.038635699 | 12 |
| GO:0005840 | ribosome | CC | 0.003664 | 0.038635699 | 47 |
| GO:0016471 | vacuolar proton-transporting V-type ATPase complex | CC | 0.003685 | 0.038635699 | 5 |
| GO:0090129 | positive regulation of synapse maturation | BP | 0.003685 | 0.038635699 | 5 |
| GO:0015238 | drug transmembrane transporter activity | MF | 0.003685 | 0.038635699 | 5 |
| GO:0021637 | trigeminal nerve structural organization | BP | 0.003714 | 0.038635699 | 3 |
| GO:0021636 | trigeminal nerve morphogenesis | BP | 0.003714 | 0.038635699 | 3 |
| GO:0001639 | PLC activating G-protein coupled glutamate receptor activity | MF | 0.003714 | 0.038635699 | 3 |
| GO:0031914 | negative regulation of synaptic plasticity | BP | 0.003714 | 0.038635699 | 3 |
| GO:0097113 | alpha-amino-3-hydroxy-5-methyl-4-isoxazole propionate receptor clustering | BP | 0.003714 | 0.038635699 | 3 |
| GO:0046870 | cadmium ion binding | MF | 0.003714 | 0.038635699 | 3 |
| GO:0015093 | ferrous iron transmembrane transporter activity | MF | 0.003714 | 0.038635699 | 3 |
| GO:0009331 | glycerol-3-phosphate dehydrogenase complex | CC | 0.003714 | 0.038635699 | 3 |
| GO:0046038 | GMP catabolic process | BP | 0.003714 | 0.038635699 | 3 |
| GO:0043103 | hypoxanthine salvage | BP | 0.003714 | 0.038635699 | 3 |
| GO:0045964 | positive regulation of dopamine metabolic process | BP | 0.003714 | 0.038635699 | 3 |
| GO:0008502 | melatonin receptor activity | MF | 0.003714 | 0.038635699 | 3 |
| GO:0046487 | glyoxylate metabolic process | BP | 0.003714 | 0.038635699 | 3 |
| GO:0045915 | positive regulation of catecholamine metabolic process | BP | 0.003714 | 0.038635699 | 3 |
| GO:0034260 | negative regulation of GTPase activity | BP | 0.003762 | 0.038840418 | 7 |
| GO:0008187 | poly-pyrimidine tract binding | MF | 0.003762 | 0.038840418 | 7 |
| GO:0097035 | regulation of membrane lipid distribution | BP | 0.003762 | 0.038840418 | 7 |
| GO:0045132 | meiotic chromosome segregation | BP | 0.003928 | 0.04016529 | 9 |
| GO:0031224 | intrinsic to membrane | CC | 0.004295 | 0.043420578 | 444 |
| GO:0050772 | positive regulation of axonogenesis | BP | 0.004311 | 0.043501068 | 12 |
| GO:0016887 | ATPase activity | MF | 0.004334 | 0.043570993 | 43 |
| GO:0009205 | purine ribonucleoside triphosphate metabolic process | BP | 0.004467 | 0.044452161 | 32 |
| GO:0042448 | progesterone metabolic process | BP | 0.004524 | 0.044452161 | 4 |
| GO:0015288 | porin activity | MF | 0.004524 | 0.044452161 | 4 |
| GO:0043121 | neurotrophin binding | MF | 0.004524 | 0.044452161 | 4 |
| GO:0046548 | retinal rod cell development | BP | 0.004524 | 0.044452161 | 4 |
| GO:0051306 | mitotic sister chromatid separation | BP | 0.004524 | 0.044452161 | 4 |
| GO:0005862 | muscle thin filament tropomyosin | CC | 0.004524 | 0.044452161 | 4 |
| GO:0051177 | meiotic sister chromatid cohesion | BP | 0.004524 | 0.044452161 | 4 |
| GO:0005665 | DNA-directed RNA polymerase II, core complex | CC | 0.004537 | 0.044452161 | 6 |
| GO:0008391 | arachidonic acid monooxygenase activity | MF | 0.004537 | 0.044452161 | 6 |
| GO:0072521 | purine-containing compound metabolic process | BP | 0.004627 | 0.04517432 | 64 |
| GO:0048025 | negative regulation of nuclear mRNA splicing, via spliceosome | BP | 0.004645 | 0.045267161 | 7 |
| GO:0033559 | unsaturated fatty acid metabolic process | BP | 0.004906 | 0.047723945 | 14 |
| GO:0042255 | ribosome assembly | BP | 0.004924 | 0.047723976 | 17 |
| GO:0008143 | poly(A) RNA binding | MF | 0.005034 | 0.04814134 | 5 |
| GO:2000463 | positive regulation of excitatory postsynaptic membrane potential | BP | 0.005034 | 0.04814134 | 5 |
| GO:0060632 | regulation of microtubule-based movement | BP | 0.005034 | 0.04814134 | 5 |
| GO:0000212 | meiotic spindle organization | BP | 0.005034 | 0.04814134 | 5 |
| GO:0018119 | peptidyl-cysteine S-nitrosylation | BP | 0.005034 | 0.04814134 | 5 |
| GO:0000287 | magnesium ion binding | MF | 0.005126 | 0.04814134 | 27 |
| GO:0051607 | defense response to virus | BP | 0.005436 | 0.04814134 | 23 |
| GO:0015399 | primary active transmembrane transporter activity | MF | 0.005547 | 0.04814134 | 17 |
| GO:0015405 | P-P-bond-hydrolysis-driven transmembrane transporter activity | MF | 0.005547 | 0.04814134 | 17 |
| GO:0021793 | chemorepulsion of branchiomotor axon | BP | 0.005582 | 0.04814134 | 2 |
| GO:0001641 | group II metabotropic glutamate receptor activity | MF | 0.005582 | 0.04814134 | 2 |
| GO:0032912 | negative regulation of transforming growth factor beta2 production | BP | 0.005582 | 0.04814134 | 2 |
| GO:2000366 | positive regulation of STAT protein import into nucleus | BP | 0.005582 | 0.04814134 | 2 |
| GO:0021551 | central nervous system morphogenesis | BP | 0.005582 | 0.04814134 | 2 |
| GO:0061026 | cardiac muscle tissue regeneration | BP | 0.005582 | 0.04814134 | 2 |
| GO:0097107 | postsynaptic density assembly | BP | 0.005582 | 0.04814134 | 2 |
| GO:0097117 | guanylate kinase-associated protein clustering | BP | 0.005582 | 0.04814134 | 2 |
| GO:0003968 | RNA-directed RNA polymerase activity | MF | 0.005582 | 0.04814134 | 2 |
| GO:0060175 | brain-derived neurotrophic factor-activated receptor activity | MF | 0.005582 | 0.04814134 | 2 |
| GO:0070826 | paraferritin complex | CC | 0.005582 | 0.04814134 | 2 |
| GO:0070627 | ferrous iron import | BP | 0.005582 | 0.04814134 | 2 |
| GO:0015676 | vanadium ion transport | BP | 0.005582 | 0.04814134 | 2 |
| GO:0015692 | lead ion transport | BP | 0.005582 | 0.04814134 | 2 |
| GO:0015100 | vanadium ion transmembrane transporter activity | MF | 0.005582 | 0.04814134 | 2 |
| GO:0015675 | nickel cation transport | BP | 0.005582 | 0.04814134 | 2 |
| GO:0060304 | regulation of phosphatidylinositol dephosphorylation | BP | 0.005582 | 0.04814134 | 2 |
| GO:0033216 | ferric iron import | BP | 0.005582 | 0.04814134 | 2 |
| GO:0002481 | antigen processing and presentation of exogenous protein antigen via MHC class Ib, TAP-dependent | BP | 0.005582 | 0.04814134 | 2 |
| GO:0071283 | cellular response to iron(III) ion | BP | 0.005582 | 0.04814134 | 2 |
| GO:0017178 | diphthine-ammonia ligase activity | MF | 0.005582 | 0.04814134 | 2 |
| GO:0035189 | Rb-E2F complex | CC | 0.005582 | 0.04814134 | 2 |
| GO:0071045 | nuclear histone mRNA catabolic process | BP | 0.005582 | 0.04814134 | 2 |
| GO:0018117 | protein adenylylation | BP | 0.005582 | 0.04814134 | 2 |
| GO:0070733 | protein adenylyltransferase activity | MF | 0.005582 | 0.04814134 | 2 |
| GO:0000121 | glycerol-1-phosphatase activity | MF | 0.005582 | 0.04814134 | 2 |
| GO:0008967 | phosphoglycolate phosphatase activity | MF | 0.005582 | 0.04814134 | 2 |
| GO:0043136 | glycerol-3-phosphatase activity | MF | 0.005582 | 0.04814134 | 2 |
| GO:0009992 | cellular water homeostasis | BP | 0.005582 | 0.04814134 | 2 |
| GO:0042292 | URM1 activating enzyme activity | MF | 0.005582 | 0.04814134 | 2 |
| GO:0032447 | protein urmylation | BP | 0.005582 | 0.04814134 | 2 |
| GO:0018192 | enzyme active site formation via L-cysteine persulfide | BP | 0.005582 | 0.04814134 | 2 |
| GO:2000208 | positive regulation of ribosomal small subunit export from nucleus | BP | 0.005582 | 0.04814134 | 2 |
| GO:0004060 | arylamine N-acetyltransferase activity | MF | 0.005582 | 0.04814134 | 2 |
| GO:0033320 | UDP-D-xylose biosynthetic process | BP | 0.005582 | 0.04814134 | 2 |
| GO:0048040 | UDP-glucuronate decarboxylase activity | MF | 0.005582 | 0.04814134 | 2 |
| GO:0030124 | AP-4 adaptor complex | CC | 0.005582 | 0.04814134 | 2 |
| GO:0009020 | tRNA (guanosine-2'-O-)-methyltransferase activity | MF | 0.005582 | 0.04814134 | 2 |
| GO:0052666 | tRNA (cytosine-2'-O-)-methyltransferase activity | MF | 0.005582 | 0.04814134 | 2 |
| GO:0018872 | arsonoacetate metabolic process | BP | 0.005582 | 0.04814134 | 2 |
| GO:0030791 | arsenite methyltransferase activity | MF | 0.005582 | 0.04814134 | 2 |
| GO:0030792 | methylarsonite methyltransferase activity | MF | 0.005582 | 0.04814134 | 2 |
| GO:0030943 | mitochondrion targeting sequence binding | MF | 0.005582 | 0.04814134 | 2 |
| GO:0031731 | CCR6 chemokine receptor binding | MF | 0.005582 | 0.04814134 | 2 |
| GO:2000364 | regulation of STAT protein import into nucleus | BP | 0.005582 | 0.04814134 | 2 |
| GO:0002428 | antigen processing and presentation of peptide antigen via MHC class Ib | BP | 0.005582 | 0.04814134 | 2 |
| GO:0002477 | antigen processing and presentation of exogenous peptide antigen via MHC class Ib | BP | 0.005582 | 0.04814134 | 2 |
| GO:0010041 | response to iron(III) ion | BP | 0.005582 | 0.04814134 | 2 |
| GO:0018175 | protein nucleotidylation | BP | 0.005582 | 0.04814134 | 2 |
| GO:0018307 | enzyme active site formation | BP | 0.005582 | 0.04814134 | 2 |
| GO:2000197 | regulation of ribonucleoprotein complex localization | BP | 0.005582 | 0.04814134 | 2 |
| GO:2000199 | positive regulation of ribonucleoprotein complex localization | BP | 0.005582 | 0.04814134 | 2 |
| GO:2000200 | regulation of ribosomal subunit export from nucleus | BP | 0.005582 | 0.04814134 | 2 |
| GO:2000202 | positive regulation of ribosomal subunit export from nucleus | BP | 0.005582 | 0.04814134 | 2 |
| GO:2000206 | regulation of ribosomal small subunit export from nucleus | BP | 0.005582 | 0.04814134 | 2 |
| GO:0033319 | UDP-D-xylose metabolic process | BP | 0.005582 | 0.04814134 | 2 |
| GO:0042842 | D-xylose biosynthetic process | BP | 0.005582 | 0.04814134 | 2 |
| GO:0009141 | nucleoside triphosphate metabolic process | BP | 0.005616 | 0.048354423 | 34 |
| GO:0018198 | peptidyl-cysteine modification | BP | 0.005675 | 0.048786579 | 7 |
| GO:0060074 | synapse maturation | BP | 0.005756 | 0.04901377 | 6 |
| GO:0070325 | lipoprotein particle receptor binding | MF | 0.005756 | 0.04901377 | 6 |
| GO:0006929 | substrate-dependent cell migration | BP | 0.005756 | 0.04901377 | 6 |

**Supplementary Table S7. GO Enrichment Analysis of Significantly Contracted Gene Families in the FMD.**

| **GO ID** | **GO Term** | **GO Class** | **p-value** | **Adjusted p-value** | **No. of genes** |
| --- | --- | --- | --- | --- | --- |
| GO:0045095 | keratin filament | CC | 5.90E-30 | 9.17E-27 | 56 |
| GO:0005882 | intermediate filament | CC | 2.81E-28 | 2.97E-25 | 84 |
| GO:0045111 | intermediate filament cytoskeleton | CC | 2.30E-21 | 8.26E-19 | 87 |
| GO:0008509 | anion transmembrane transporter activity | MF | 1.39E-19 | 4.46E-17 | 90 |
| GO:0031424 | keratinization | BP | 1.90E-18 | 5.37E-16 | 58 |
| GO:0031224 | intrinsic to membrane | CC | 2.10E-16 | 5.16E-14 | 1566 |
| GO:0016712 | oxidoreductase activity, acting on paired donors, with incorporation or reduction of molecular oxygen, reduced flavin or flavoprotein as one donor, and incorporation of one atom of oxygen | MF | 5.99E-15 | 1.21E-12 | 27 |
| GO:0070330 | aromatase activity | MF | 7.71E-15 | 1.53E-12 | 26 |
| GO:0016021 | integral to membrane | CC | 8.07E-15 | 1.57E-12 | 1529 |
| GO:0030216 | keratinocyte differentiation | BP | 1.06E-13 | 1.87E-11 | 71 |
| GO:0009913 | epidermal cell differentiation | BP | 1.27E-11 | 2.00E-09 | 71 |
| GO:0007156 | homophilic cell adhesion | BP | 4.35E-11 | 6.44E-09 | 59 |
| GO:0005253 | anion channel activity | MF | 4.49E-11 | 6.55E-09 | 43 |
| GO:0008392 | arachidonic acid epoxygenase activity | MF | 4.89E-11 | 6.92E-09 | 19 |
| GO:0008391 | arachidonic acid monooxygenase activity | MF | 4.89E-11 | 6.92E-09 | 19 |
| GO:0005452 | inorganic anion exchanger activity | MF | 9.13E-11 | 1.25E-08 | 19 |
| GO:0004888 | transmembrane signaling receptor activity | MF | 1.66E-10 | 2.15E-08 | 609 |
| GO:0019373 | epoxygenase P450 pathway | BP | 2.28E-10 | 2.83E-08 | 19 |
| GO:0007338 | single fertilization | BP | 7.88E-10 | 8.95E-08 | 56 |
| GO:0009566 | fertilization | BP | 8.42E-10 | 9.13E-08 | 65 |
| GO:0038023 | signaling receptor activity | MF | 8.51E-10 | 9.13E-08 | 622 |
| GO:0044425 | membrane part | CC | 9.13E-10 | 9.68E-08 | 1740 |
| GO:0005254 | chloride channel activity | MF | 1.35E-09 | 1.40E-07 | 37 |
| GO:0015301 | anion:anion antiporter activity | MF | 1.96E-09 | 1.97E-07 | 23 |
| GO:0004872 | receptor activity | MF | 2.63E-09 | 2.58E-07 | 667 |
| GO:0015181 | arginine transmembrane transporter activity | MF | 3.28E-09 | 3.09E-07 | 15 |
| GO:0005342 | organic acid transmembrane transporter activity | MF | 3.75E-09 | 3.46E-07 | 62 |
| GO:0004930 | G-protein coupled receptor activity | MF | 6.25E-09 | 5.50E-07 | 530 |
| GO:0015189 | L-lysine transmembrane transporter activity | MF | 1.44E-08 | 1.15E-06 | 14 |
| GO:0008544 | epidermis development | BP | 2.14E-08 | 1.69E-06 | 93 |
| GO:0006690 | icosanoid metabolic process | BP | 2.25E-08 | 1.75E-06 | 37 |
| GO:0022804 | active transmembrane transporter activity | MF | 3.13E-08 | 2.33E-06 | 129 |
| GO:0004871 | signal transducer activity | MF | 4.54E-08 | 3.23E-06 | 665 |
| GO:0060089 | molecular transducer activity | MF | 4.54E-08 | 3.23E-06 | 665 |
| GO:0004866 | endopeptidase inhibitor activity | MF | 5.23E-08 | 3.69E-06 | 58 |
| GO:0030414 | peptidase inhibitor activity | MF | 6.27E-08 | 4.40E-06 | 61 |
| GO:0015179 | L-amino acid transmembrane transporter activity | MF | 7.26E-08 | 5.02E-06 | 30 |
| GO:0045503 | dynein light chain binding | MF | 1.18E-07 | 7.97E-06 | 17 |
| GO:0004984 | olfactory receptor activity | MF | 1.65E-07 | 1.08E-05 | 422 |
| GO:0015171 | amino acid transmembrane transporter activity | MF | 1.89E-07 | 1.19E-05 | 40 |
| GO:0020002 | host cell plasma membrane | CC | 2.43E-07 | 1.46E-05 | 14 |
| GO:0019028 | viral capsid | CC | 2.43E-07 | 1.46E-05 | 14 |
| GO:0005179 | hormone activity | MF | 2.95E-07 | 1.72E-05 | 46 |
| GO:0061135 | endopeptidase regulator activity | MF | 3.42E-07 | 1.97E-05 | 58 |
| GO:0004867 | serine-type endopeptidase inhibitor activity | MF | 4.33E-07 | 2.40E-05 | 40 |
| GO:0033559 | unsaturated fatty acid metabolic process | BP | 4.86E-07 | 2.67E-05 | 39 |
| GO:0015174 | basic amino acid transmembrane transporter activity | MF | 5.70E-07 | 3.11E-05 | 15 |
| GO:0033644 | host cell membrane | CC | 7.51E-07 | 3.96E-05 | 14 |
| GO:0015296 | anion:cation symporter activity | MF | 8.21E-07 | 4.28E-05 | 17 |
| GO:0019013 | viral nucleocapsid | CC | 9.43E-07 | 4.81E-05 | 13 |
| GO:0005549 | odorant binding | MF | 1.49E-06 | 7.35E-05 | 95 |
| GO:0008514 | organic anion transmembrane transporter activity | MF | 1.91E-06 | 9.23E-05 | 21 |
| GO:0051959 | dynein light intermediate chain binding | MF | 1.98E-06 | 9.51E-05 | 17 |
| GO:0044218 | other organism cell membrane | CC | 2.03E-06 | 9.59E-05 | 14 |
| GO:0044279 | other organism membrane | CC | 2.03E-06 | 9.59E-05 | 14 |
| GO:0044423 | virion part | CC | 2.03E-06 | 9.59E-05 | 14 |
| GO:0008569 | minus-end-directed microtubule motor activity | MF | 2.76E-06 | 0.000124004 | 13 |
| GO:0043252 | sodium-independent organic anion transport | BP | 3.46E-06 | 0.000152997 | 16 |
| GO:0046943 | carboxylic acid transmembrane transporter activity | MF | 3.85E-06 | 0.000167813 | 52 |
| GO:0070268 | cornification | BP | 3.94E-06 | 0.000170915 | 24 |
| GO:0001518 | voltage-gated sodium channel complex | CC | 4.41E-06 | 0.000188026 | 11 |
| GO:0061134 | peptidase regulator activity | MF | 4.57E-06 | 0.000193027 | 63 |
| GO:0004497 | monooxygenase activity | MF | 5.89E-06 | 0.000240886 | 42 |
| GO:0045505 | dynein intermediate chain binding | MF | 6.05E-06 | 0.000244221 | 15 |
| GO:0015347 | sodium-independent organic anion transmembrane transporter activity | MF | 6.05E-06 | 0.000244221 | 15 |
| GO:0004857 | enzyme inhibitor activity | MF | 6.23E-06 | 0.000250602 | 100 |
| GO:0019369 | arachidonic acid metabolic process | BP | 6.57E-06 | 0.000262127 | 20 |
| GO:0034707 | chloride channel complex | CC | 6.96E-06 | 0.000273848 | 23 |
| GO:0006952 | defense response | BP | 7.82E-06 | 0.000301483 | 292 |
| GO:0008510 | sodium:bicarbonate symporter activity | MF | 1.36E-05 | 0.000483094 | 7 |
| GO:0004499 | N,N-dimethylaniline monooxygenase activity | MF | 1.36E-05 | 0.000483094 | 7 |
| GO:0035174 | histone serine kinase activity | MF | 1.36E-05 | 0.000483094 | 7 |
| GO:0022891 | substrate-specific transmembrane transporter activity | MF | 1.64E-05 | 0.000565012 | 233 |
| GO:0015747 | urate transport | BP | 1.73E-05 | 0.000594017 | 10 |
| GO:0005858 | axonemal dynein complex | CC | 2.02E-05 | 0.000675393 | 8 |
| GO:0022857 | transmembrane transporter activity | MF | 2.09E-05 | 0.000694687 | 256 |
| GO:0019012 | virion | CC | 2.31E-05 | 0.000753609 | 14 |
| GO:0005275 | amine transmembrane transporter activity | MF | 2.37E-05 | 0.000771639 | 41 |
| GO:0005887 | integral to plasma membrane | CC | 2.92E-05 | 0.000924829 | 321 |
| GO:0042742 | defense response to bacterium | BP | 3.40E-05 | 0.001048249 | 64 |
| GO:0005615 | extracellular space | CC | 3.54E-05 | 0.001080008 | 301 |
| GO:0015081 | sodium ion transmembrane transporter activity | MF | 3.88E-05 | 0.00116251 | 38 |
| GO:0006863 | purine base transport | BP | 4.96E-05 | 0.0014455 | 10 |
| GO:0015297 | antiporter activity | MF | 5.39E-05 | 0.001533158 | 34 |
| GO:0004890 | GABA-A receptor activity | MF | 5.41E-05 | 0.001534329 | 12 |
| GO:0015294 | solute:cation symporter activity | MF | 6.30E-05 | 0.001744174 | 35 |
| GO:0015143 | urate transmembrane transporter activity | MF | 6.66E-05 | 0.001821901 | 9 |
| GO:0006691 | leukotriene metabolic process | BP | 7.07E-05 | 0.001916816 | 13 |
| GO:0043449 | cellular alkene metabolic process | BP | 7.07E-05 | 0.001916816 | 13 |
| GO:0001594 | trace-amine receptor activity | MF | 8.30E-05 | 0.002217488 | 8 |
| GO:0015075 | ion transmembrane transporter activity | MF | 8.64E-05 | 0.002302517 | 193 |
| GO:0015103 | inorganic anion transmembrane transporter activity | MF | 9.01E-05 | 0.002387597 | 22 |
| GO:0000038 | very long-chain fatty acid metabolic process | BP | 9.52E-05 | 0.002474913 | 28 |
| GO:0015300 | solute:solute antiporter activity | MF | 9.52E-05 | 0.002474913 | 28 |
| GO:0031226 | intrinsic to plasma membrane | CC | 0.000100078 | 0.002561159 | 330 |
| GO:0005328 | neurotransmitter:sodium symporter activity | MF | 0.000110391 | 0.002775853 | 12 |
| GO:0022412 | cellular process involved in reproduction in multicellular organism | BP | 0.00011174 | 0.002794694 | 14 |
| GO:0015851 | nucleobase transport | BP | 0.000121725 | 0.003028197 | 10 |
| GO:0015291 | secondary active transmembrane transporter activity | MF | 0.000136023 | 0.003313204 | 71 |
| GO:0045502 | dynein binding | MF | 0.000155456 | 0.00369962 | 21 |
| GO:0034706 | sodium channel complex | CC | 0.000171761 | 0.004036165 | 11 |
| GO:0005229 | intracellular calcium activated chloride channel activity | MF | 0.000171761 | 0.004036165 | 11 |
| GO:0007129 | synapsis | BP | 0.000186531 | 0.00435038 | 19 |
| GO:0019076 | release of virus from host | BP | 0.000201179 | 0.00458874 | 13 |
| GO:0008009 | chemokine activity | MF | 0.000212268 | 0.004794795 | 17 |
| GO:0019825 | oxygen binding | MF | 0.000216482 | 0.004843225 | 14 |
| GO:0015020 | glucuronosyltransferase activity | MF | 0.000216482 | 0.004843225 | 14 |
| GO:0005576 | extracellular region | CC | 0.000216489 | 0.004843225 | 848 |
| GO:0016307 | phosphatidylinositol phosphate kinase activity | MF | 0.000265751 | 0.005819703 | 10 |
| GO:0071944 | cell periphery | CC | 0.000295295 | 0.006376875 | 1321 |
| GO:0020037 | heme binding | MF | 0.000317348 | 0.006759217 | 45 |
| GO:0005886 | plasma membrane | CC | 0.00033202 | 0.007000618 | 1299 |
| GO:0050501 | hyaluronan synthase activity | MF | 0.000333935 | 0.007000618 | 5 |
| GO:0004459 | L-lactate dehydrogenase activity | MF | 0.000333935 | 0.007000618 | 5 |
| GO:0008404 | arachidonic acid 14,15-epoxygenase activity | MF | 0.000333935 | 0.007000618 | 5 |
| GO:0008405 | arachidonic acid 11,12-epoxygenase activity | MF | 0.000333935 | 0.007000618 | 5 |
| GO:0071614 | linoleic acid epoxygenase activity | MF | 0.000333935 | 0.007000618 | 5 |
| GO:0005248 | voltage-gated sodium channel activity | MF | 0.000377786 | 0.007814558 | 12 |
| GO:0016917 | GABA receptor activity | MF | 0.000377786 | 0.007814558 | 12 |
| GO:0035005 | 1-phosphatidylinositol-4-phosphate 3-kinase activity | MF | 0.000389814 | 0.008009345 | 6 |
| GO:0033643 | host cell part | CC | 0.000398184 | 0.008089503 | 14 |
| GO:0030246 | carbohydrate binding | MF | 0.000432522 | 0.008714897 | 112 |
| GO:0042379 | chemokine receptor binding | MF | 0.000460533 | 0.009219557 | 19 |
| GO:0019953 | sexual reproduction | BP | 0.00049474 | 0.009820066 | 161 |
| GO:0030286 | dynein complex | CC | 0.000567425 | 0.010959648 | 17 |
| GO:0007186 | G-protein coupled receptor signaling pathway | BP | 0.000578668 | 0.011130711 | 418 |
| GO:0032982 | myosin filament | CC | 0.000606256 | 0.011542376 | 11 |
| GO:0007342 | fusion of sperm to egg plasma membrane | BP | 0.000616812 | 0.01171848 | 8 |
| GO:0007340 | acrosome reaction | BP | 0.000664596 | 0.012400027 | 15 |
| GO:0007606 | sensory perception of chemical stimulus | BP | 0.000817231 | 0.014803785 | 282 |
| GO:1900004 | negative regulation of serine-type endopeptidase activity | BP | 0.000911815 | 0.01626447 | 7 |
| GO:1900003 | regulation of serine-type endopeptidase activity | BP | 0.000911815 | 0.01626447 | 7 |
| GO:0015106 | bicarbonate transmembrane transporter activity | MF | 0.001041836 | 0.018133006 | 11 |
| GO:0007214 | gamma-aminobutyric acid signaling pathway | BP | 0.001041836 | 0.018133006 | 11 |
| GO:0046906 | tetrapyrrole binding | MF | 0.001086974 | 0.018709195 | 46 |
| GO:0050707 | regulation of cytokine secretion | BP | 0.001161244 | 0.019732692 | 31 |
| GO:0008395 | steroid hydroxylase activity | MF | 0.001247718 | 0.0208578 | 13 |
| GO:0006955 | immune response | BP | 0.001281502 | 0.021310388 | 310 |
| GO:0005326 | neurotransmitter transporter activity | MF | 0.001286939 | 0.021354961 | 12 |
| GO:0008970 | phosphatidylcholine 1-acylhydrolase activity | MF | 0.001291049 | 0.021354961 | 6 |
| GO:0004022 | alcohol dehydrogenase (NAD) activity | MF | 0.001291049 | 0.021354961 | 6 |
| GO:0051707 | response to other organism | BP | 0.001456589 | 0.023632204 | 156 |
| GO:0005230 | extracellular ligand-gated ion channel activity | MF | 0.001504747 | 0.024174656 | 19 |
| GO:0005605 | basal lamina | CC | 0.001570442 | 0.025086729 | 9 |
| GO:0007416 | synapse assembly | BP | 0.001584551 | 0.025268853 | 37 |
| GO:0005332 | gamma-aminobutyric acid:sodium symporter activity | MF | 0.001656184 | 0.025833309 | 4 |
| GO:0052871 | alpha-tocopherol omega-hydroxylase activity | MF | 0.001656184 | 0.025833309 | 4 |
| GO:0045226 | extracellular polysaccharide biosynthetic process | BP | 0.001656184 | 0.025833309 | 4 |
| GO:0044245 | polysaccharide digestion | BP | 0.001656184 | 0.025833309 | 4 |
| GO:0060005 | vestibular reflex | BP | 0.001656184 | 0.025833309 | 4 |
| GO:0004528 | phosphodiesterase I activity | MF | 0.001656184 | 0.025833309 | 4 |
| GO:0052870 | tocopherol omega-hydroxylase activity | MF | 0.001656184 | 0.025833309 | 4 |
| GO:0046379 | extracellular polysaccharide metabolic process | BP | 0.001656184 | 0.025833309 | 4 |
| GO:0060012 | synaptic transmission, glycinergic | BP | 0.001667022 | 0.025833309 | 5 |
| GO:0005225 | volume-sensitive anion channel activity | MF | 0.001667022 | 0.025833309 | 5 |
| GO:0004565 | beta-galactosidase activity | MF | 0.001667022 | 0.025833309 | 5 |
| GO:0045575 | basophil activation | BP | 0.001667022 | 0.025833309 | 5 |
| GO:0005343 | organic acid:sodium symporter activity | MF | 0.001687186 | 0.026059206 | 10 |
| GO:0044216 | other organism cell | CC | 0.001717721 | 0.026313009 | 15 |
| GO:0043245 | extraorganismal space | CC | 0.001717721 | 0.026313009 | 15 |
| GO:0044215 | other organism | CC | 0.001717721 | 0.026313009 | 15 |
| GO:0044217 | other organism part | CC | 0.001717721 | 0.026313009 | 15 |
| GO:0009607 | response to biotic stimulus | BP | 0.001836419 | 0.02785683 | 163 |
| GO:0018995 | host | CC | 0.001905418 | 0.028670396 | 14 |
| GO:0043657 | host cell | CC | 0.001905418 | 0.028670396 | 14 |
| GO:0070820 | tertiary granule | CC | 0.002011023 | 0.029873934 | 17 |
| GO:0005283 | sodium:amino acid symporter activity | MF | 0.002069988 | 0.030506986 | 7 |
| GO:0005391 | sodium:potassium-exchanging ATPase activity | MF | 0.002069988 | 0.030506986 | 7 |
| GO:0008556 | potassium-transporting ATPase activity | MF | 0.002069988 | 0.030506986 | 7 |
| GO:0005201 | extracellular matrix structural constituent | MF | 0.002088532 | 0.030683331 | 23 |
| GO:0007608 | sensory perception of smell | BP | 0.002100931 | 0.030768577 | 193 |
| GO:0022892 | substrate-specific transporter activity | MF | 0.002152495 | 0.031375973 | 256 |
| GO:0070821 | tertiary granule membrane | CC | 0.002308801 | 0.033187679 | 16 |
| GO:0006805 | xenobiotic metabolic process | BP | 0.00250705 | 0.035383157 | 18 |
| GO:0006865 | amino acid transport | BP | 0.002590981 | 0.036402505 | 33 |
| GO:0045087 | innate immune response | BP | 0.00265911 | 0.037135983 | 152 |
| GO:0042611 | MHC protein complex | CC | 0.002678059 | 0.037344706 | 11 |
| GO:0007130 | synaptonemal complex assembly | BP | 0.002768116 | 0.038435113 | 10 |
| GO:0007339 | binding of sperm to zona pellucida | BP | 0.002981216 | 0.041080896 | 14 |
| GO:0030213 | hyaluronan biosynthetic process | BP | 0.00320992 | 0.043110399 | 6 |
| GO:0004415 | hyalurononglucosaminidase activity | MF | 0.00320992 | 0.043110399 | 6 |
| GO:0001676 | long-chain fatty acid metabolic process | BP | 0.003211676 | 0.043110399 | 28 |
| GO:0043225 | anion transmembrane-transporting ATPase activity | MF | 0.003685915 | 0.048227064 | 12 |

**Supplementary Table S8. GO Enrichment Analysis of Positively Selected Genes in the FMD.**

| Term | Number of genes | p-value | Corrected p-value |
| --- | --- | --- | --- |
| intracellular | 319 | 8.33E-30 | 1.92E-26 |
| organelle | 295 | 9.08E-30 | 1.92E-26 |
| membrane-bounded organelle | 279 | 5.50E-29 | 7.75E-26 |
| intracellular part | 305 | 1.45E-28 | 1.53E-25 |
| intracellular organelle | 273 | 6.27E-28 | 5.30E-25 |
| cell | 359 | 1.39E-27 | 9.35E-25 |
| cell part | 358 | 1.55E-27 | 9.35E-25 |
| intracellular membrane-bounded organelle | 255 | 5.13E-27 | 2.71E-24 |
| single-organism process | 311 | 5.93E-26 | 2.78E-23 |
| cellular process | 351 | 1.38E-25 | 5.82E-23 |
| metabolic process | 259 | 1.01E-21 | 3.88E-19 |
| biological regulation | 260 | 1.23E-20 | 4.34E-18 |
| cytoplasm | 224 | 2.65E-20 | 8.63E-18 |
| single-organism cellular process | 259 | 2.73E-19 | 8.24E-17 |
| organic substance metabolic process | 238 | 3.83E-19 | 1.08E-16 |
| primary metabolic process | 228 | 1.50E-18 | 3.96E-16 |
| nucleus | 160 | 4.90E-18 | 1.22E-15 |
| cellular metabolic process | 224 | 1.89E-17 | 4.44E-15 |
| regulation of biological process | 237 | 2.90E-17 | 6.45E-15 |
| binding | 255 | 3.90E-17 | 8.24E-15 |
| macromolecule metabolic process | 203 | 4.15E-17 | 8.35E-15 |
| cellular macromolecule metabolic process | 189 | 2.86E-16 | 5.49E-14 |
| cytoplasmic part | 160 | 7.55E-16 | 1.39E-13 |
| organelle part | 163 | 4.09E-15 | 7.20E-13 |
| intracellular organelle part | 158 | 3.43E-14 | 5.79E-12 |
| regulation of cellular process | 214 | 2.69E-13 | 4.37E-11 |
| single-multicellular organism process | 122 | 3.11E-13 | 4.87E-11 |
| protein binding | 135 | 3.25E-13 | 4.91E-11 |
| heterocycle metabolic process | 125 | 1.56E-12 | 2.27E-10 |
| cellular aromatic compound metabolic process | 126 | 1.81E-12 | 2.56E-10 |
| positive regulation of biological process | 115 | 2.11E-12 | 2.81E-10 |
| nucleobase-containing compound metabolic process | 123 | 2.13E-12 | 2.81E-10 |
| nuclear part | 93 | 3.89E-12 | 4.98E-10 |
| single-organism developmental process | 116 | 4.76E-12 | 5.91E-10 |
| developmental process | 117 | 5.36E-12 | 6.47E-10 |
| anatomical structure development | 111 | 8.39E-12 | 9.85E-10 |
| organic cyclic compound metabolic process | 126 | 1.01E-11 | 1.15E-09 |
| nuclear lumen | 83 | 1.64E-11 | 1.80E-09 |
| cellular component organization or biogenesis | 123 | 1.71E-11 | 1.80E-09 |
| organelle lumen | 90 | 1.79E-11 | 1.80E-09 |
| intracellular organelle lumen | 90 | 1.79E-11 | 1.80E-09 |
| membrane-enclosed lumen | 90 | 1.79E-11 | 1.80E-09 |
| cellular nitrogen compound metabolic process | 134 | 2.05E-11 | 2.01E-09 |
| macromolecule modification | 97 | 2.17E-11 | 2.09E-09 |
| nucleic acid metabolic process | 108 | 4.06E-11 | 3.81E-09 |
| response to stimulus | 170 | 5.96E-11 | 5.38E-09 |
| cellular component organization | 117 | 5.99E-11 | 5.38E-09 |
| multicellular organismal process | 143 | 6.82E-11 | 6.00E-09 |
| nitrogen compound metabolic process | 137 | 1.74E-10 | 1.50E-08 |
| membrane | 193 | 1.80E-10 | 1.52E-08 |
| extracellular region | 104 | 1.92E-10 | 1.59E-08 |
| positive regulation of cellular process | 101 | 3.10E-10 | 2.52E-08 |
| catalytic activity | 136 | 4.70E-10 | 3.75E-08 |
| extracellular region part | 90 | 9.28E-10 | 7.26E-08 |
| cellular developmental process | 83 | 1.05E-09 | 8.06E-08 |
| system development | 87 | 1.19E-09 | 8.99E-08 |
| organelle organization | 79 | 1.69E-09 | 1.25E-07 |
| RNA metabolic process | 94 | 2.83E-09 | 2.06E-07 |
| macromolecular complex | 111 | 3.01E-09 | 2.16E-07 |
| intracellular non-membrane-bounded organelle | 83 | 4.09E-09 | 2.83E-07 |
| non-membrane-bounded organelle | 83 | 4.09E-09 | 2.83E-07 |
| multicellular organism development | 92 | 4.16E-09 | 2.84E-07 |
| regulation of metabolic process | 117 | 4.37E-09 | 2.93E-07 |
| regulation of macromolecule metabolic process | 112 | 4.54E-09 | 3.00E-07 |
| negative regulation of biological process | 93 | 5.17E-09 | 3.36E-07 |
| protein modification process | 86 | 7.53E-09 | 4.75E-07 |
| cellular protein modification process | 86 | 7.53E-09 | 4.75E-07 |
| protein metabolic process | 117 | 7.86E-09 | 4.88E-07 |
| localization | 112 | 8.31E-09 | 5.09E-07 |
| regulation of cellular metabolic process | 112 | 9.80E-09 | 5.91E-07 |
| nucleic acid binding | 90 | 1.05E-08 | 6.27E-07 |
| regulation of primary metabolic process | 111 | 1.09E-08 | 6.41E-07 |
| cell differentiation | 75 | 1.44E-08 | 8.32E-07 |
| gene expression | 105 | 1.88E-08 | 1.08E-06 |
| positive regulation of cellular metabolic process | 66 | 2.94E-08 | 1.65E-06 |
| cellular component biogenesis | 61 | 3.01E-08 | 1.68E-06 |
| protein complex | 91 | 3.16E-08 | 1.73E-06 |
| negative regulation of cellular process | 86 | 3.58E-08 | 1.94E-06 |
| positive regulation of metabolic process | 68 | 3.67E-08 | 1.96E-06 |
| single-organism metabolic process | 90 | 4.04E-08 | 2.13E-06 |
| signal transduction | 123 | 4.11E-08 | 2.14E-06 |
| anatomical structure morphogenesis | 57 | 4.55E-08 | 2.34E-06 |
| aromatic compound biosynthetic process | 83 | 4.61E-08 | 2.34E-06 |
| single organism signaling | 127 | 4.76E-08 | 2.39E-06 |
| cellular protein metabolic process | 105 | 4.88E-08 | 2.43E-06 |
| signaling | 127 | 5.18E-08 | 2.54E-06 |
| regulation of response to stimulus | 72 | 5.84E-08 | 2.83E-06 |
| cellular response to stimulus | 139 | 7.61E-08 | 3.66E-06 |
| nucleobase-containing compound biosynthetic process | 81 | 7.86E-08 | 3.71E-06 |
| membrane part | 152 | 7.98E-08 | 3.71E-06 |
| heterocycle biosynthetic process | 82 | 7.99E-08 | 3.71E-06 |
| endomembrane system | 71 | 9.64E-08 | 4.43E-06 |
| positive regulation of macromolecule metabolic process | 64 | 1.01E-07 | 4.60E-06 |
| organic cyclic compound biosynthetic process | 83 | 1.30E-07 | 5.86E-06 |
| cell communication | 126 | 1.44E-07 | 6.40E-06 |
| nucleoplasm | 55 | 2.26E-07 | 9.94E-06 |
| biosynthetic process | 114 | 2.31E-07 | 1.01E-05 |
| response to stress | 70 | 3.13E-07 | 1.35E-05 |
| cellular component assembly | 53 | 3.25E-07 | 1.39E-05 |
| organic substance biosynthetic process | 111 | 3.83E-07 | 1.62E-05 |
| heterocyclic compound binding | 116 | 4.00E-07 | 1.67E-05 |
| regulation of biological quality | 67 | 4.11E-07 | 1.70E-05 |
| cell death | 45 | 5.03E-07 | 2.06E-05 |
| cellular biosynthetic process | 109 | 5.07E-07 | 2.06E-05 |
| regulation of molecular function | 59 | 5.11E-07 | 2.06E-05 |
| intracellular signal transduction | 57 | 7.44E-07 | 2.96E-05 |
| organic cyclic compound binding | 116 | 9.85E-07 | 3.89E-05 |
| regulation of protein modification process | 43 | 1.09E-06 | 4.26E-05 |
| positive regulation of cellular protein metabolic process | 38 | 1.24E-06 | 4.80E-05 |
| positive regulation of response to stimulus | 45 | 1.29E-06 | 4.97E-05 |
| apoptotic process | 42 | 1.40E-06 | 5.33E-05 |
| cellular component assembly involved in morphogenesis | 14 | 1.42E-06 | 5.34E-05 |
| regulation of multicellular organismal process | 54 | 1.45E-06 | 5.43E-05 |
| receptor binding | 43 | 1.58E-06 | 5.84E-05 |
| programmed cell death | 42 | 1.73E-06 | 6.34E-05 |
| regulation of catalytic activity | 49 | 1.75E-06 | 6.34E-05 |
| cellular nitrogen compound biosynthetic process | 90 | 1.76E-06 | 6.34E-05 |
| integral component of membrane | 131 | 1.77E-06 | 6.34E-05 |
| positive regulation of protein metabolic process | 39 | 1.92E-06 | 6.82E-05 |
| cellular component morphogenesis | 32 | 1.95E-06 | 6.88E-05 |
| animal organ development | 61 | 1.98E-06 | 6.91E-05 |
| cell surface receptor signaling pathway | 56 | 2.15E-06 | 7.42E-05 |
| cell adhesion | 34 | 2.16E-06 | 7.42E-05 |
| chromosome | 28 | 2.25E-06 | 7.67E-05 |
| intrinsic component of membrane | 131 | 2.50E-06 | 8.44E-05 |
| cellular macromolecule biosynthetic process | 90 | 2.58E-06 | 8.66E-05 |
| biological adhesion | 34 | 2.76E-06 | 9.20E-05 |
| anatomical structure formation involved in morphogenesis | 31 | 3.44E-06 | 0.000113573 |
| methylation | 16 | 3.55E-06 | 0.000116185 |
| regulation of signal transduction | 55 | 3.67E-06 | 0.00011934 |
| regulation of nucleobase-containing compound metabolic process | 72 | 3.84E-06 | 0.000123863 |
| protein localization | 46 | 3.92E-06 | 0.000125316 |
| transcription, DNA-templated | 67 | 4.00E-06 | 0.000127106 |
| transferase activity | 58 | 4.32E-06 | 0.000136236 |
| macromolecule localization | 51 | 4.37E-06 | 0.000136707 |
| macromolecule biosynthetic process | 91 | 4.57E-06 | 0.000141618 |
| apoptotic signaling pathway | 21 | 4.59E-06 | 0.000141618 |
| vacuole | 30 | 4.66E-06 | 0.00014209 |
| cellular protein localization | 37 | 4.67E-06 | 0.00014209 |
| positive regulation of signaling | 38 | 4.76E-06 | 0.000143362 |
| macromolecular complex subunit organization | 47 | 4.80E-06 | 0.000143362 |
| positive regulation of signal transduction | 36 | 4.82E-06 | 0.000143362 |
| cellular macromolecule localization | 37 | 5.21E-06 | 0.000154002 |
| positive regulation of protein modification process | 31 | 5.39E-06 | 0.000158159 |
| chromosomal part | 26 | 5.44E-06 | 0.000158513 |
| RNA binding | 43 | 5.49E-06 | 0.000158877 |
| tissue development | 40 | 5.63E-06 | 0.000161891 |
| DNA metabolic process | 26 | 5.86E-06 | 0.000167168 |
| cytokine production | 20 | 5.90E-06 | 0.000167244 |
| poly(A) RNA binding | 34 | 5.98E-06 | 0.000168449 |
| phosphate-containing compound metabolic process | 64 | 6.02E-06 | 0.000168449 |
| nucleic acid-templated transcription | 67 | 6.25E-06 | 0.000173641 |
| regulation of transcription, DNA-templated | 64 | 6.46E-06 | 0.000178267 |
| regulation of gene expression | 74 | 6.52E-06 | 0.000178909 |
| phosphorus metabolic process | 64 | 6.68E-06 | 0.000182195 |
| RNA biosynthetic process | 67 | 7.64E-06 | 0.000206898 |
| regulation of nitrogen compound metabolic process | 75 | 7.75E-06 | 0.000208427 |
| RNA processing | 26 | 7.83E-06 | 0.000209139 |
| macromolecule methylation | 13 | 7.87E-06 | 0.000209139 |
| protein ubiquitination | 23 | 8.85E-06 | 0.0002336 |
| positive regulation of cell communication | 37 | 1.03E-05 | 0.000271361 |
| chromosome organization | 28 | 1.04E-05 | 0.000271361 |
| regulation of nucleic acid-templated transcription | 64 | 1.08E-05 | 0.000279524 |
| regulation of RNA biosynthetic process | 64 | 1.15E-05 | 0.000296103 |
| protein modification by small protein conjugation or removal | 27 | 1.16E-05 | 0.000296103 |
| cellular localization | 46 | 1.19E-05 | 0.000302954 |
| immune system process | 50 | 1.28E-05 | 0.000322906 |
| positive regulation of catalytic activity | 32 | 1.31E-05 | 0.00033009 |
| regulation of cellular protein metabolic process | 53 | 1.32E-05 | 0.00033053 |
| negative regulation of nucleic acid-templated transcription | 30 | 1.35E-05 | 0.0003364 |
| single-organism organelle organization | 39 | 1.52E-05 | 0.000374665 |
| vesicle | 64 | 1.55E-05 | 0.000381513 |
| regulation of protein metabolic process | 55 | 1.64E-05 | 0.000400977 |
| positive regulation of intracellular signal transduction | 26 | 1.67E-05 | 0.000406433 |
| cell morphogenesis | 28 | 1.73E-05 | 0.000418277 |
| regulation of signaling | 56 | 1.78E-05 | 0.000426351 |
| regulation of cellular macromolecule biosynthetic process | 68 | 1.80E-05 | 0.000429119 |
| regulation of RNA metabolic process | 65 | 1.87E-05 | 0.000441344 |
| negative regulation of RNA biosynthetic process | 30 | 1.93E-05 | 0.000452695 |
| regulation of cellular biosynthetic process | 72 | 2.08E-05 | 0.000484541 |
| ncRNA metabolic process | 19 | 2.09E-05 | 0.000484541 |
| regulation of phosphorylation | 35 | 2.11E-05 | 0.000486874 |
| establishment of localization | 83 | 2.13E-05 | 0.000489873 |
| positive regulation of multicellular organismal process | 33 | 2.20E-05 | 0.000502193 |
| protein phosphorylation | 43 | 2.23E-05 | 0.000506228 |
| catabolic process | 40 | 2.36E-05 | 0.000532611 |
| negative regulation of transcription, DNA-templated | 29 | 2.47E-05 | 0.000556145 |
| organic substance catabolic process | 39 | 2.53E-05 | 0.000566409 |
| regulation of cell communication | 55 | 2.61E-05 | 0.000580973 |
| positive regulation of molecular function | 36 | 2.68E-05 | 0.000591145 |
| endoplasmic reticulum | 32 | 2.69E-05 | 0.000591145 |
| regulation of protein phosphorylation | 33 | 2.86E-05 | 0.00062541 |
| regulation of biosynthetic process | 72 | 3.03E-05 | 0.000658665 |
| negative regulation of RNA metabolic process | 30 | 3.04E-05 | 0.000658665 |
| regulation of macromolecule biosynthetic process | 69 | 3.06E-05 | 0.000658665 |
| regulation of phosphate metabolic process | 38 | 3.58E-05 | 0.000763163 |
| regulation of phosphorus metabolic process | 38 | 3.58E-05 | 0.000763163 |
| protein modification by small protein conjugation | 23 | 3.62E-05 | 0.000769004 |
| response to organic substance | 50 | 3.72E-05 | 0.000785213 |
| mitochondrion | 46 | 3.87E-05 | 0.000814443 |
| endosome | 20 | 3.93E-05 | 0.000822205 |
| transport | 80 | 4.30E-05 | 0.000894801 |
| negative regulation of cellular macromolecule biosynthetic process | 31 | 4.75E-05 | 0.000980915 |
| negative regulation of macromolecule biosynthetic process | 32 | 4.76E-05 | 0.000980915 |
| regulation of intracellular signal transduction | 36 | 4.95E-05 | 0.001015886 |
| regulation of transcription from RNA polymerase II promoter | 42 | 5.34E-05 | 0.001086289 |
| organelle assembly | 20 | 5.35E-05 | 0.001086289 |
| regulation of cytokine production | 17 | 5.54E-05 | 0.001115758 |
| negative regulation of nucleobase-containing compound metabolic process | 31 | 5.55E-05 | 0.001115758 |
| response to oxygen-containing compound | 27 | 5.78E-05 | 0.001156776 |
| DNA binding | 45 | 6.48E-05 | 0.001291794 |
| cellular response to chemical stimulus | 49 | 6.55E-05 | 0.001299436 |
| nuclear chromosome | 18 | 6.98E-05 | 0.001373242 |
| negative regulation of macromolecule metabolic process | 45 | 6.99E-05 | 0.001373242 |
| membrane-bounded vesicle | 60 | 7.17E-05 | 0.001403311 |
| identical protein binding | 27 | 7.25E-05 | 0.001410814 |
| transcription from RNA polymerase II promoter | 43 | 7.53E-05 | 0.001459921 |
| cell projection morphogenesis | 19 | 7.74E-05 | 0.001493537 |
| negative regulation of cellular biosynthetic process | 32 | 9.91E-05 | 0.001902661 |
| cell proliferation | 39 | 0.0001 | 0.001915507 |
| phosphorylation | 47 | 0.000102 | 0.001950489 |
| regulation of cell proliferation | 33 | 0.000106 | 0.002013147 |
| cilium assembly | 9 | 0.00011 | 0.00207527 |
| cell part morphogenesis | 19 | 0.000112 | 0.002110403 |
| apoptotic mitochondrial changes | 8 | 0.000117 | 0.002193805 |
| macromolecular complex assembly | 33 | 0.000122 | 0.00226616 |
| negative regulation of biosynthetic process | 32 | 0.000131 | 0.002426739 |
| lysosome | 16 | 0.000133 | 0.002451934 |
| lytic vacuole | 16 | 0.000133 | 0.002451934 |
| chromatin organization | 20 | 0.000136 | 0.002486063 |
| ncRNA processing | 15 | 0.000148 | 0.002689238 |
| CD4-positive, alpha-beta T cell activation | 6 | 0.00016 | 0.002881222 |
| CD4-positive, alpha-beta T cell differentiation | 6 | 0.00016 | 0.002881222 |
| alpha-beta T cell activation | 7 | 0.000166 | 0.002974011 |
| intracellular vesicle | 23 | 0.000177 | 0.003169022 |
| negative regulation of gene expression | 31 | 0.000185 | 0.003291401 |
| proteinaceous extracellular matrix | 11 | 0.000186 | 0.003291401 |
| positive regulation of cell adhesion | 12 | 0.000192 | 0.003381037 |
| negative regulation of metabolic process | 46 | 0.000196 | 0.003451408 |
| nervous system development | 37 | 0.000201 | 0.003522534 |
| histone H3-K4 methylation | 5 | 0.000203 | 0.003535265 |
| RNA modification | 9 | 0.000206 | 0.00357601 |
| positive regulation of phosphate metabolic process | 26 | 0.000209 | 0.003603602 |
| positive regulation of phosphorus metabolic process | 26 | 0.000209 | 0.003603602 |
| negative regulation of transcription from RNA polymerase II promoter | 21 | 0.000217 | 0.00371995 |
| negative regulation of cellular metabolic process | 44 | 0.000218 | 0.003724606 |
| cell projection organization | 24 | 0.000229 | 0.003886249 |
| cellular macromolecular complex assembly | 25 | 0.000231 | 0.003906479 |
| negative regulation of nitrogen compound metabolic process | 31 | 0.000232 | 0.003906479 |
| cell projection assembly | 12 | 0.000237 | 0.003981872 |
| cilium organization | 9 | 0.000239 | 0.003982282 |
| catalytic complex | 30 | 0.000239 | 0.003982282 |
| whole membrane | 26 | 0.000244 | 0.004052277 |
| positive regulation of immune system process | 20 | 0.000245 | 0.004057809 |
| histone modification | 14 | 0.000253 | 0.004172304 |
| nuclear chromosome part | 16 | 0.000255 | 0.004177802 |
| regulation of histone H3-K4 methylation | 4 | 0.000257 | 0.004207251 |
| regulation of cell death | 32 | 0.000301 | 0.004890599 |
| covalent chromatin modification | 14 | 0.000302 | 0.004890599 |
| cell-cell adhesion | 21 | 0.000312 | 0.005032943 |
| enzyme binding | 33 | 0.000313 | 0.005036446 |
| muscle structure development | 17 | 0.000317 | 0.005063545 |
| cilium morphogenesis | 9 | 0.000317 | 0.005063545 |
| chromatin | 16 | 0.00032 | 0.005082862 |
| establishment of protein localization | 33 | 0.000326 | 0.005149814 |
| defense response | 32 | 0.000328 | 0.005149814 |
| positive regulation of protein phosphorylation | 23 | 0.000329 | 0.005149814 |
| regulation of immune system process | 26 | 0.000329 | 0.005149814 |
| regulation of transferase activity | 21 | 0.00035 | 0.005458992 |
| extracellular vesicle | 49 | 0.00037 | 0.005744849 |
| nuclear outer membrane-endoplasmic reticulum membrane network | 19 | 0.00038 | 0.00583411 |
| cardiovascular system development | 22 | 0.000381 | 0.00583411 |
| circulatory system development | 22 | 0.000381 | 0.00583411 |
| extracellular organelle | 49 | 0.000381 | 0.00583411 |
| enzyme regulator activity | 25 | 0.000386 | 0.005869766 |
| extracellular space | 35 | 0.000386 | 0.005869766 |
| alpha-beta T cell differentiation involved in immune response | 5 | 0.000391 | 0.005886214 |
| alpha-beta T cell activation involved in immune response | 5 | 0.000391 | 0.005886214 |
| CD4-positive, alpha-beta T cell differentiation involved in immune response | 5 | 0.000391 | 0.005886214 |
| transferase activity, transferring acyl groups other than amino-acyl groups | 10 | 0.000402 | 0.006011025 |
| cytoplasmic vesicle | 22 | 0.000403 | 0.006011025 |
| chromosomal region | 11 | 0.000408 | 0.006064433 |
| immune response | 30 | 0.000422 | 0.006262799 |
| regulation of apoptotic signaling pathway | 13 | 0.000434 | 0.006405041 |
| multi-organism process | 39 | 0.000448 | 0.006580799 |
| macromolecule catabolic process | 26 | 0.00046 | 0.006742707 |
| positive regulation of cell-cell adhesion | 9 | 0.000473 | 0.006904788 |
| locomotion | 28 | 0.000476 | 0.006908013 |
| positive regulation of phosphorylation | 23 | 0.000477 | 0.006908013 |
| epithelium development | 24 | 0.000499 | 0.007208944 |
| nucleolus | 23 | 0.000502 | 0.007223819 |
| nuclear chromatin | 12 | 0.000521 | 0.007450512 |
| T cell differentiation involved in immune response | 5 | 0.000525 | 0.007450512 |
| positive regulation of T cell activation | 8 | 0.000526 | 0.007450512 |
| proteolysis | 36 | 0.000527 | 0.007450512 |
| cell activation | 22 | 0.000527 | 0.007450512 |
| cell recognition | 7 | 0.000528 | 0.007450512 |
| alpha-beta T cell differentiation | 6 | 0.000536 | 0.00753546 |
| regulation of protein kinase activity | 18 | 0.000552 | 0.007719752 |
| protein dimerization activity | 26 | 0.000554 | 0.007719752 |
| extracellular exosome | 48 | 0.000555 | 0.007719752 |
| positive regulation of cell proliferation | 20 | 0.000568 | 0.007886556 |
| single organismal cell-cell adhesion | 19 | 0.000582 | 0.008043456 |
| cytosol | 33 | 0.000588 | 0.008099257 |
| positive regulation of leukocyte cell-cell adhesion | 8 | 0.000607 | 0.008342373 |
| cellular response to stress | 33 | 0.00061 | 0.008357548 |
| regulation of system process | 14 | 0.000628 | 0.008519736 |
| tRNA metabolic process | 10 | 0.000631 | 0.008519736 |
| protein-DNA complex subunit organization | 10 | 0.000631 | 0.008519736 |
| positive regulation of cell activation | 10 | 0.000631 | 0.008519736 |
| palate development | 6 | 0.00066 | 0.008874513 |
| regulation of cell adhesion | 15 | 0.000673 | 0.009023782 |
| extracellular matrix | 12 | 0.000683 | 0.009137667 |
| regulation of alpha-beta T cell activation | 5 | 0.000689 | 0.009152575 |
| regulation of histone methylation | 5 | 0.000689 | 0.009152575 |
| regulation of leukocyte cell-cell adhesion | 10 | 0.000703 | 0.009309962 |
| protein complex biogenesis | 25 | 0.000717 | 0.00943817 |
| protein complex assembly | 25 | 0.000717 | 0.00943817 |
| protein complex subunit organization | 27 | 0.000732 | 0.009603083 |
| cellular response to organic substance | 39 | 0.000742 | 0.009676524 |
| regulation of developmental process | 39 | 0.000742 | 0.009676524 |
| ion binding | 67 | 0.000756 | 0.009821955 |
| T cell differentiation | 9 | 0.000774 | 0.010005035 |
| positive regulation of lymphocyte activation | 9 | 0.000774 | 0.010005035 |
| cellular response to DNA damage stimulus | 19 | 0.000777 | 0.010012864 |
| cell projection | 24 | 0.000808 | 0.010371633 |
| MAPK cascade | 19 | 0.000823 | 0.010532038 |
| cell periphery | 81 | 0.00084 | 0.010711274 |
| positive regulation of transferase activity | 14 | 0.000849 | 0.01079366 |
| movement of cell or subcellular component | 29 | 0.000856 | 0.010843098 |
| positive regulation of cell morphogenesis involved in differentiation | 7 | 0.000863 | 0.010900549 |
| signal transduction by protein phosphorylation | 20 | 0.000883 | 0.011125385 |
| endoplasmic reticulum membrane | 18 | 0.000895 | 0.011239231 |
| positive regulation of gene expression | 33 | 0.000916 | 0.011316249 |
| regulation of CD4-positive, alpha-beta T cell differentiation | 4 | 0.00092 | 0.011316249 |
| regulation of CD4-positive, alpha-beta T cell activation | 4 | 0.00092 | 0.011316249 |
| O-acyltransferase activity | 4 | 0.00092 | 0.011316249 |
| single organism cell adhesion | 19 | 0.00092 | 0.011316249 |
| positive regulation of cell differentiation | 19 | 0.00092 | 0.011316249 |
| endoplasmic reticulum part | 19 | 0.00092 | 0.011316249 |
| transcription factor activity, transcription factor binding | 15 | 0.000954 | 0.01169434 |
| regulation of apoptotic process | 29 | 0.000964 | 0.011789415 |
| molecular function regulator | 28 | 0.000976 | 0.011869367 |
| carbohydrate derivative metabolic process | 28 | 0.000976 | 0.011869367 |
| cell development | 36 | 0.000985 | 0.011938606 |
| blood circulation | 13 | 0.000989 | 0.011959517 |
| positive regulation of leukocyte proliferation | 7 | 0.001006 | 0.012054162 |
| positive regulation of lymphocyte proliferation | 7 | 0.001006 | 0.012054162 |
| positive regulation of mononuclear cell proliferation | 7 | 0.001006 | 0.012054162 |
| sequence-specific DNA binding | 26 | 0.001029 | 0.012298211 |
| transcription corepressor activity | 8 | 0.001039 | 0.01236135 |
| regulation of programmed cell death | 29 | 0.001043 | 0.012374201 |
| cellular catabolic process | 30 | 0.001063 | 0.012557956 |
| regulation of kinase activity | 18 | 0.001064 | 0.012557956 |
| regulation of cellular component organization | 39 | 0.001089 | 0.012765418 |
| contractile fiber | 9 | 0.00109 | 0.012765418 |
| transcription factor activity, protein binding | 15 | 0.001091 | 0.012765418 |
| intracellular protein transport | 22 | 0.001122 | 0.013091576 |
| intracellular transport | 28 | 0.001145 | 0.013331319 |
| circulatory system process | 13 | 0.001152 | 0.013372826 |
| positive regulation of T cell proliferation | 6 | 0.001165 | 0.01348995 |
| regulation of cell-cell adhesion | 11 | 0.001178 | 0.013596277 |
| establishment of localization in cell | 32 | 0.001212 | 0.013914248 |
| positive regulation of nitrogen compound metabolic process | 34 | 0.001214 | 0.013914248 |
| cytokine activity | 14 | 0.001215 | 0.013914248 |
| carbohydrate derivative biosynthetic process | 21 | 0.001258 | 0.014364834 |
| organic substance transport | 41 | 0.001263 | 0.014379995 |
| phosphatidylinositol-3,5-bisphosphate binding | 4 | 0.001287 | 0.014539761 |
| positive T cell selection | 4 | 0.001287 | 0.014539761 |
| regulation of alpha-beta T cell differentiation | 4 | 0.001287 | 0.014539761 |
| protein transport | 29 | 0.001313 | 0.014790236 |
| contractile fiber part | 8 | 0.001331 | 0.014919959 |
| late endosome | 8 | 0.001331 | 0.014919959 |
| cellular response to oxidative stress | 7 | 0.001346 | 0.0150479 |
| tRNA modification | 6 | 0.001387 | 0.015462313 |
| RNA methyltransferase activity | 5 | 0.001414 | 0.015722537 |
| transcription cofactor activity | 14 | 0.001492 | 0.016546947 |
| serine-type peptidase activity | 9 | 0.001503 | 0.016592456 |
| regulation of organelle organization | 23 | 0.001504 | 0.016592456 |
| positive regulation of MAPK cascade | 13 | 0.001545 | 0.017000802 |
| organelle membrane | 38 | 0.001554 | 0.017049202 |
| protein localization to organelle | 19 | 0.001651 | 0.017970132 |
| cation binding | 63 | 0.00166 | 0.017970132 |
| positive regulation of leukocyte activation | 9 | 0.001665 | 0.017970132 |
| protein-DNA complex assembly | 9 | 0.001665 | 0.017970132 |
| regulation of T cell activation | 9 | 0.001665 | 0.017970132 |
| serine hydrolase activity | 9 | 0.001665 | 0.017970132 |
| positive regulation of kinase activity | 12 | 0.001686 | 0.018145983 |
| positive regulation of nucleobase-containing compound metabolic process | 32 | 0.001715 | 0.018346818 |
| oxidoreductase activity | 24 | 0.001715 | 0.018346818 |
| positive regulation of alpha-beta T cell activation | 4 | 0.001746 | 0.018564529 |
| T cell lineage commitment | 4 | 0.001746 | 0.018564529 |
| collagen binding | 5 | 0.001749 | 0.018564529 |
| regulation of T cell proliferation | 7 | 0.001771 | 0.018751703 |
| positive regulation of developmental process | 24 | 0.001789 | 0.018845956 |
| positive regulation of transcription from RNA polymerase II promoter | 24 | 0.001789 | 0.018845956 |
| transferase activity, transferring acyl groups | 10 | 0.001864 | 0.019588008 |
| regulation of mononuclear cell proliferation | 8 | 0.001889 | 0.019758269 |
| regulation of lymphocyte proliferation | 8 | 0.001889 | 0.019758269 |
| acetyltransferase activity | 6 | 0.001925 | 0.020055394 |
| leukocyte cell-cell adhesion | 15 | 0.001932 | 0.020055394 |
| protein heterodimerization activity | 14 | 0.001941 | 0.020055394 |
| positive regulation of programmed cell death | 14 | 0.001941 | 0.020055394 |
| positive regulation of apoptotic process | 14 | 0.001941 | 0.020055394 |
| peroxisome | 7 | 0.002019 | 0.020701816 |
| microbody | 7 | 0.002019 | 0.020701816 |
| DNA-dependent DNA replication | 7 | 0.002019 | 0.020701816 |
| positive regulation of hydrolase activity | 17 | 0.002034 | 0.02080691 |
| transcription factor binding | 13 | 0.002043 | 0.020852814 |
| hydrolase activity | 48 | 0.002089 | 0.021264584 |
| regulation of leukocyte proliferation | 8 | 0.002111 | 0.021421024 |
| protein import | 11 | 0.002114 | 0.021421024 |
| histone lysine methylation | 5 | 0.002138 | 0.021610728 |
| transferase activity, transferring one-carbon groups | 9 | 0.002238 | 0.022571419 |
| regulation of anatomical structure morphogenesis | 20 | 0.002279 | 0.022921854 |
| chromosome, telomeric region | 7 | 0.002293 | 0.022953056 |
| regulation of chromatin organization | 7 | 0.002293 | 0.022953056 |
| T-helper cell differentiation | 4 | 0.002307 | 0.023022581 |
| inflammatory response | 15 | 0.00231 | 0.023022581 |
| heart development | 13 | 0.002338 | 0.023242915 |
| DNA recombination | 8 | 0.002354 | 0.023315491 |
| plasma membrane | 77 | 0.00239 | 0.02361606 |
| positive regulation of cell death | 14 | 0.002497 | 0.024624952 |
| RNA methylation | 5 | 0.002587 | 0.025272897 |
| N-acetyltransferase activity | 5 | 0.002587 | 0.025272897 |
| tRNA processing | 7 | 0.002594 | 0.025272897 |
| establishment of protein localization to organelle | 15 | 0.002596 | 0.025272897 |
| metal ion binding | 61 | 0.002598 | 0.025272897 |
| actomyosin structure organization | 6 | 0.002608 | 0.025272897 |
| cell motility | 23 | 0.002614 | 0.025272897 |
| localization of cell | 23 | 0.002614 | 0.025272897 |
| myofibril | 8 | 0.002617 | 0.025272897 |
| positive regulation of proteolysis | 10 | 0.002631 | 0.025347194 |
| organophosphate metabolic process | 21 | 0.002753 | 0.02646104 |
| peptidase activity, acting on L-amino acid peptides | 17 | 0.002789 | 0.026749865 |
| positive regulation of cellular biosynthetic process | 32 | 0.002805 | 0.026843513 |
| regulation of protein complex assembly | 11 | 0.002876 | 0.027458722 |
| response to external stimulus | 35 | 0.002915 | 0.027766467 |
| cyclic purine nucleotide metabolic process | 7 | 0.002925 | 0.027805865 |
| positive regulation of CD4-positive, alpha-beta T cell differentiation | 3 | 0.002941 | 0.02782641 |
| positive regulation of CD4-positive, alpha-beta T cell activation | 3 | 0.002941 | 0.02782641 |
| positive regulation of T cell differentiation | 4 | 0.002981 | 0.028018693 |
| cGMP biosynthetic process | 4 | 0.002981 | 0.028018693 |
| T cell selection | 4 | 0.002981 | 0.028018693 |
| nuclear transport | 12 | 0.003001 | 0.028078019 |
| nucleocytoplasmic transport | 12 | 0.003001 | 0.028078019 |
| oxidation-reduction process | 26 | 0.003089 | 0.028516331 |
| positive regulation of protein kinase activity | 11 | 0.003097 | 0.028516331 |
| regulation of lymphocyte activation | 10 | 0.003099 | 0.028516331 |
| regulation of T cell differentiation | 5 | 0.0031 | 0.028516331 |
| heterochromatin | 5 | 0.0031 | 0.028516331 |
| peptidyl-lysine methylation | 5 | 0.0031 | 0.028516331 |
| regulation of cell differentiation | 27 | 0.003101 | 0.028516331 |
| cytoplasmic, membrane-bounded vesicle | 18 | 0.003185 | 0.0292251 |
| single-organism localization | 47 | 0.003207 | 0.029283154 |
| regulation of innate immune response | 8 | 0.003212 | 0.029283154 |
| regulation of protein modification by small protein conjugation or removal | 8 | 0.003212 | 0.029283154 |
| DNA repair | 13 | 0.00323 | 0.029377974 |
| positive regulation of innate immune response | 7 | 0.003288 | 0.029714596 |
| cyclic nucleotide biosynthetic process | 7 | 0.003288 | 0.029714596 |
| sarcomere | 7 | 0.003288 | 0.029714596 |
| regulation of localization | 38 | 0.003297 | 0.029731828 |
| positive regulation of biosynthetic process | 32 | 0.003384 | 0.030448734 |
| homeostatic process | 28 | 0.003433 | 0.030770826 |
| regulation of cell morphogenesis | 12 | 0.003434 | 0.030770826 |
| lymphocyte activation | 16 | 0.003448 | 0.030785213 |
| epithelial to mesenchymal transition | 6 | 0.003457 | 0.030785213 |
| positive regulation of protein modification by small protein conjugation or removal | 6 | 0.003457 | 0.030785213 |
| extrinsic apoptotic signaling pathway | 9 | 0.003532 | 0.031385635 |
| integral component of organelle membrane | 8 | 0.003547 | 0.031429196 |
| protein catabolic process | 19 | 0.003552 | 0.031429196 |
| peptidase activity | 17 | 0.003584 | 0.03164613 |
| cellular response to oxygen-containing compound | 16 | 0.003631 | 0.031931985 |
| extrinsic apoptotic signaling pathway via death domain receptors | 5 | 0.003683 | 0.032315589 |
| cytoskeleton organization | 20 | 0.003724 | 0.032605192 |
| cGMP metabolic process | 4 | 0.003778 | 0.033010698 |
| bounding membrane of organelle | 25 | 0.003817 | 0.033281259 |
| activation of protein kinase activity | 8 | 0.003908 | 0.033905106 |
| intrinsic component of organelle membrane | 8 | 0.003908 | 0.033905106 |
| purine-containing compound biosynthetic process | 10 | 0.003924 | 0.033970277 |
| regulation of histone modification | 6 | 0.003952 | 0.034146279 |
| single-organism biosynthetic process | 26 | 0.004103 | 0.035376928 |
| ciliary part | 7 | 0.004115 | 0.035405707 |
| regulation of hormone levels | 11 | 0.004123 | 0.035405707 |
| double-stranded DNA binding | 17 | 0.004145 | 0.035522669 |
| response to chemical | 70 | 0.00423 | 0.036139048 |
| positive regulation of cytokine production | 10 | 0.004234 | 0.036139048 |
| tropomyosin binding | 3 | 0.004282 | 0.036331227 |
| positive regulation of alpha-beta T cell differentiation | 3 | 0.004282 | 0.036331227 |
| T-helper 17 cell lineage commitment | 3 | 0.004282 | 0.036331227 |
| single-stranded DNA binding | 5 | 0.00434 | 0.03674704 |
| protein import into nucleus | 9 | 0.004552 | 0.038201201 |
| single-organism nuclear import | 9 | 0.004552 | 0.038201201 |
| response to oxidative stress | 9 | 0.004552 | 0.038201201 |
| protein targeting to nucleus | 9 | 0.004552 | 0.038201201 |
| oxidoreductase activity, acting on CH-OH group of donors | 7 | 0.004583 | 0.03838022 |
| transferase complex | 19 | 0.004625 | 0.038655021 |
| negative regulation of protein modification process | 13 | 0.004646 | 0.038754451 |
| single-organism catabolic process | 16 | 0.004669 | 0.038793321 |
| sequence-specific double-stranded DNA binding | 16 | 0.004669 | 0.038793321 |
| vesicle-mediated transport | 23 | 0.004687 | 0.038857214 |
| regulation of chemokine production | 4 | 0.004707 | 0.038857214 |
| histone ubiquitination | 4 | 0.004707 | 0.038857214 |
| adaptive immune response based on somatic recombination of immune receptors built from immunoglobulin superfamily domains | 8 | 0.004715 | 0.038857214 |
| regulation of cell activation | 11 | 0.004729 | 0.038857214 |
| small molecule metabolic process | 35 | 0.004736 | 0.038857214 |
| regulation of GTPase activity | 12 | 0.004741 | 0.038857214 |
| leukocyte activation | 17 | 0.004776 | 0.0390693 |
| sexual reproduction | 16 | 0.004903 | 0.040025506 |
| regulation of response to stress | 21 | 0.004912 | 0.040025506 |
| negative regulation of transferase activity | 9 | 0.004939 | 0.040169134 |
| organ morphogenesis | 20 | 0.00499 | 0.040506405 |
| enzyme linked receptor protein signaling pathway | 19 | 0.005037 | 0.040807138 |
| positive regulation of chromatin organization | 5 | 0.005077 | 0.040969643 |
| regulation of protein ubiquitination | 7 | 0.00509 | 0.040969643 |
| negative regulation of protein complex assembly | 6 | 0.005096 | 0.040969643 |
| positive regulation of I-kappaB kinase/NF-kappaB signaling | 8 | 0.005163 | 0.041194523 |
| regulation of chromosome organization | 8 | 0.005163 | 0.041194523 |
| secretory granule | 8 | 0.005163 | 0.041194523 |
| methyltransferase activity | 8 | 0.005163 | 0.041194523 |
| T cell activation | 13 | 0.005216 | 0.041228999 |
| organophosphate biosynthetic process | 13 | 0.005216 | 0.041228999 |
| T cell aggregation | 13 | 0.005216 | 0.041228999 |
| neuron projection | 13 | 0.005216 | 0.041228999 |
| lymphocyte aggregation | 13 | 0.005216 | 0.041228999 |
| transcription regulatory region DNA binding | 18 | 0.005271 | 0.041587803 |
| nucleotide biosynthetic process | 10 | 0.005283 | 0.041600634 |
| nuclear import | 9 | 0.005351 | 0.04205957 |
| enzyme activator activity | 11 | 0.005403 | 0.042388469 |
| cytoskeleton | 28 | 0.005593 | 0.043544307 |
| peptidyl-amino acid modification | 24 | 0.00563 | 0.043544307 |
| T cell proliferation | 7 | 0.005638 | 0.043544307 |
| positive regulation of apoptotic signaling pathway | 7 | 0.005638 | 0.043544307 |
| cytoplasmic vesicle part | 8 | 0.005643 | 0.043544307 |
| negative regulation of protein kinase activity | 8 | 0.005643 | 0.043544307 |
| rRNA processing | 8 | 0.005643 | 0.043544307 |
| rRNA metabolic process | 8 | 0.005643 | 0.043544307 |
| positive regulation of RNA metabolic process | 27 | 0.005679 | 0.04374568 |
| regulatory region DNA binding | 18 | 0.00575 | 0.044127656 |
| regulatory region nucleic acid binding | 18 | 0.00575 | 0.044127656 |
| DNA modification | 4 | 0.005779 | 0.044229925 |
| N-acyltransferase activity | 5 | 0.005898 | 0.044771597 |
| CD4-positive or CD8-positive, alpha-beta T cell lineage commitment | 3 | 0.00594 | 0.044771597 |
| T-helper 17 type immune response | 3 | 0.00594 | 0.044771597 |
| H4 histone acetyltransferase activity | 3 | 0.00594 | 0.044771597 |
| T-helper cell lineage commitment | 3 | 0.00594 | 0.044771597 |
| T-helper 17 cell differentiation | 3 | 0.00594 | 0.044771597 |
| DNA ligation | 3 | 0.00594 | 0.044771597 |
| CD4-positive, alpha-beta T cell lineage commitment | 3 | 0.00594 | 0.044771597 |
| single organism reproductive process | 22 | 0.006021 | 0.04530636 |
| nucleoside phosphate biosynthetic process | 10 | 0.006087 | 0.045666615 |
| cell morphogenesis involved in differentiation | 15 | 0.006091 | 0.045666615 |
| positive regulation of transcription, DNA-templated | 26 | 0.006137 | 0.045783669 |
| positive regulation of nucleic acid-templated transcription | 26 | 0.006137 | 0.045783669 |
| regulation of multicellular organismal development | 28 | 0.006139 | 0.045783669 |
| positive regulation of GTPase activity | 11 | 0.00615 | 0.045788405 |
| purine ribonucleotide metabolic process | 13 | 0.006174 | 0.045886015 |
| positive regulation of macromolecule biosynthetic process | 29 | 0.006202 | 0.046008775 |
| positive regulation of RNA biosynthetic process | 26 | 0.006339 | 0.046946226 |
| regulation of cellular component movement | 15 | 0.006397 | 0.047289617 |
| calcium ion import | 6 | 0.006465 | 0.047710262 |
| ribonucleotide metabolic process | 13 | 0.006523 | 0.047972615 |
| leukocyte aggregation | 13 | 0.006523 | 0.047972615 |
| negative regulation of phosphorylation | 11 | 0.006553 | 0.048110908 |
| response to inorganic substance | 8 | 0.006704 | 0.049130483 |
| purine ribonucleotide biosynthetic process | 9 | 0.006746 | 0.049354853 |
| chromatin binding | 12 | 0.006803 | 0.049462905 |
| histone methylation | 5 | 0.006808 | 0.049462905 |
| positive regulation of JAK-STAT cascade | 5 | 0.006808 | 0.049462905 |
| positive regulation of STAT cascade | 5 | 0.006808 | 0.049462905 |
| RNA polymerase II transcription factor activity, sequence-specific DNA binding | 16 | 0.006824 | 0.049495639 |
| purine-containing compound metabolic process | 14 | 0.006847 | 0.049573628 |
| cyclic nucleotide metabolic process | 7 | 0.006865 | 0.049573628 |
| ribose phosphate metabolic process | 13 | 0.006888 | 0.049573628 |
| regulation of immune response | 13 | 0.006888 | 0.049573628 |

**Supplementary Table S9. Activity of *ADA* (U/mgprot) Expressed in *E. coli*.**

|  | 1 | 2 | 3 |
| --- | --- | --- | --- |
| wild *ADA* | 45109.61 | 45654.16 | 46462.2 |
| mutant *ADA* | 16306.33 | 15135.94 | 15229.95 |

# Supplementary Figures:

**
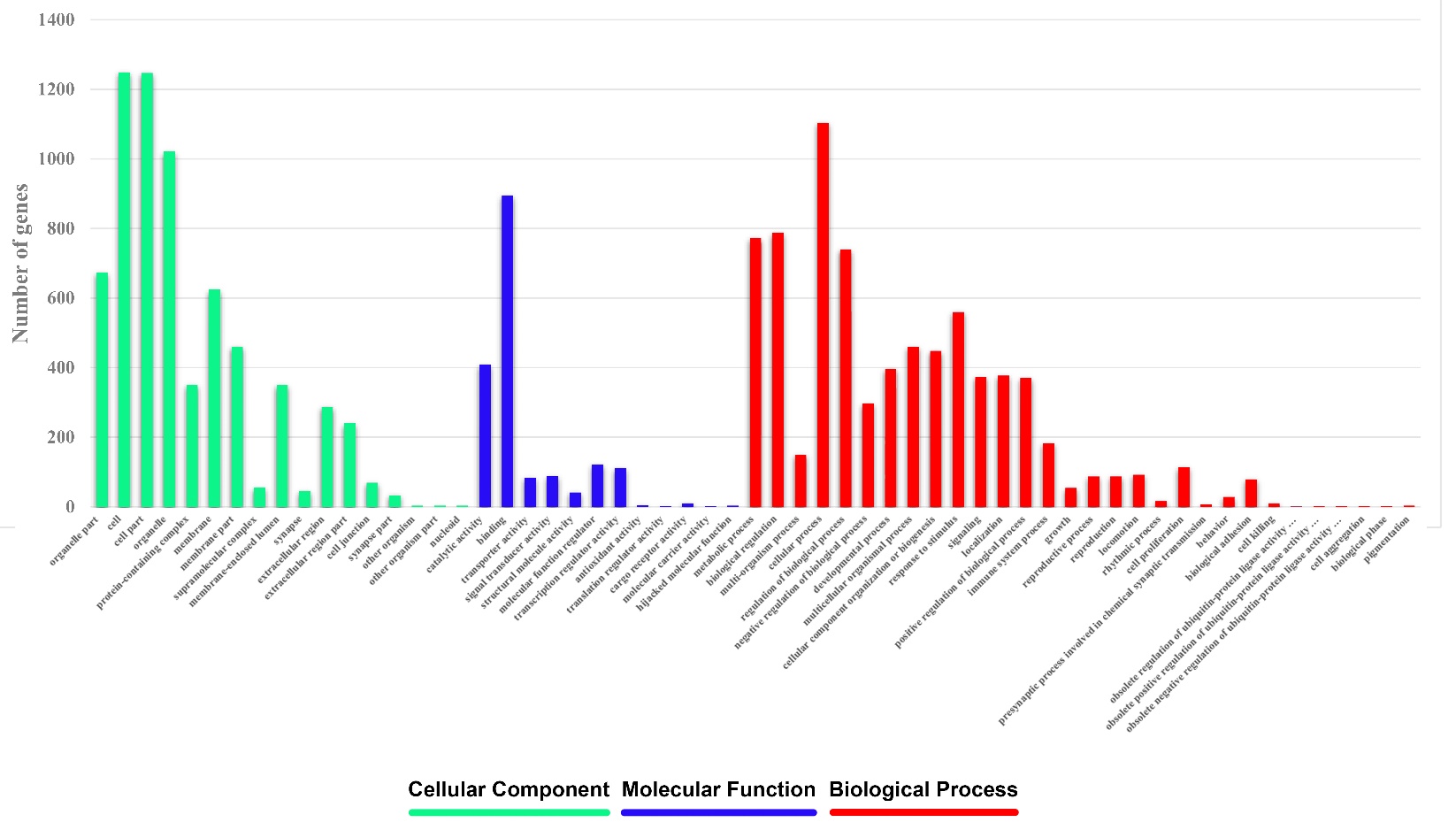
**

**F_IG_. S1.** Functional distribution of PSGs in FMD according to the Gene Ontology (GO) database. The y-axis shows the number of genes in each category, while the GO functional categories are plotted on the x-axis.


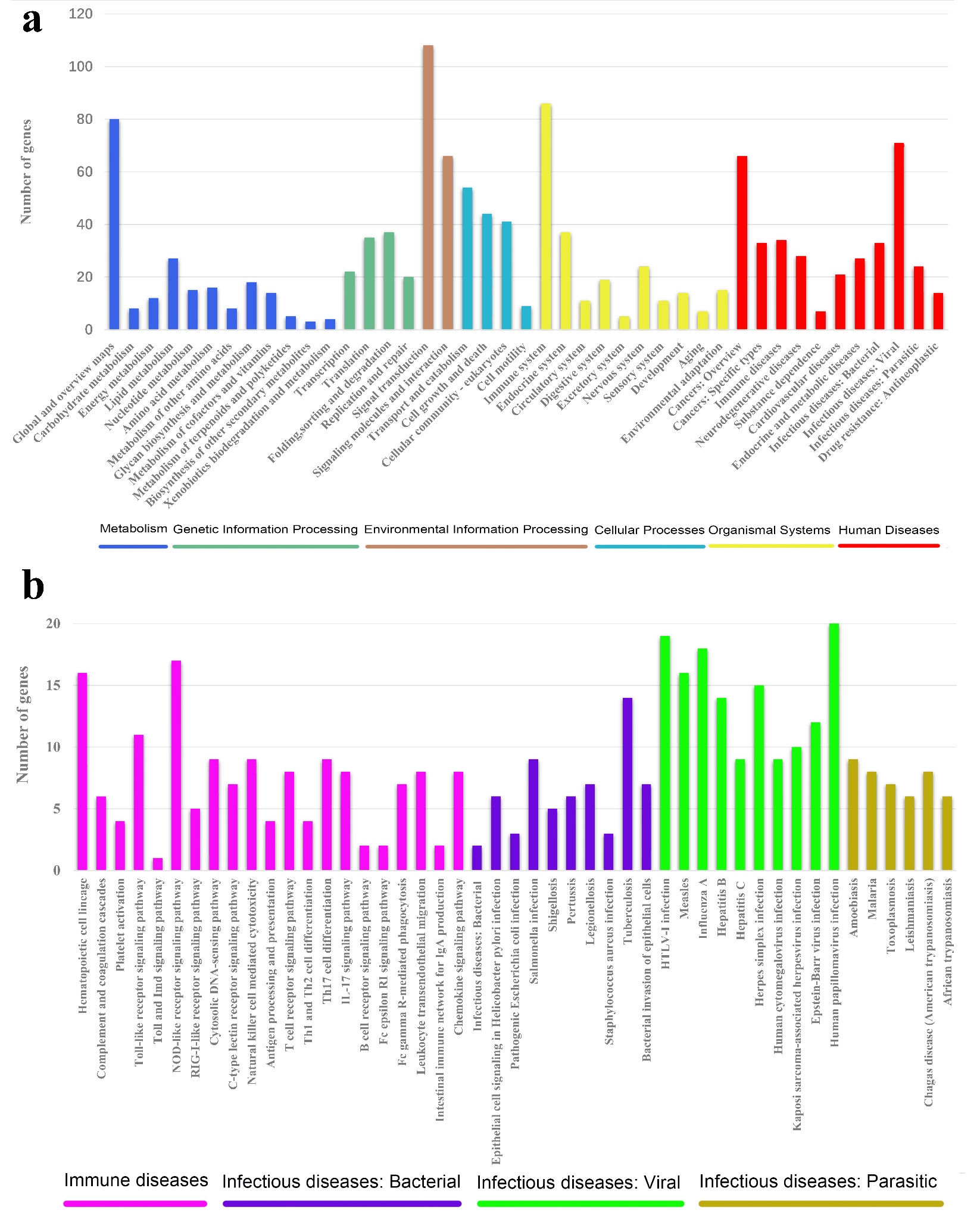


**F_IG_. S2.** Functional distribution of PSGs in FMD according to the KEGG pathway database. The y-axis shows the number of genes in each category, while the KEGG functional categories are plotted on the x-axis. (a) Functional distribution of PSGs in metabolism, genetic information processing, environmental information processing, cellular processes, organismal systems, and human diseases. (b) Functional distribution of PSGs in immune diseases and infectious diseases.


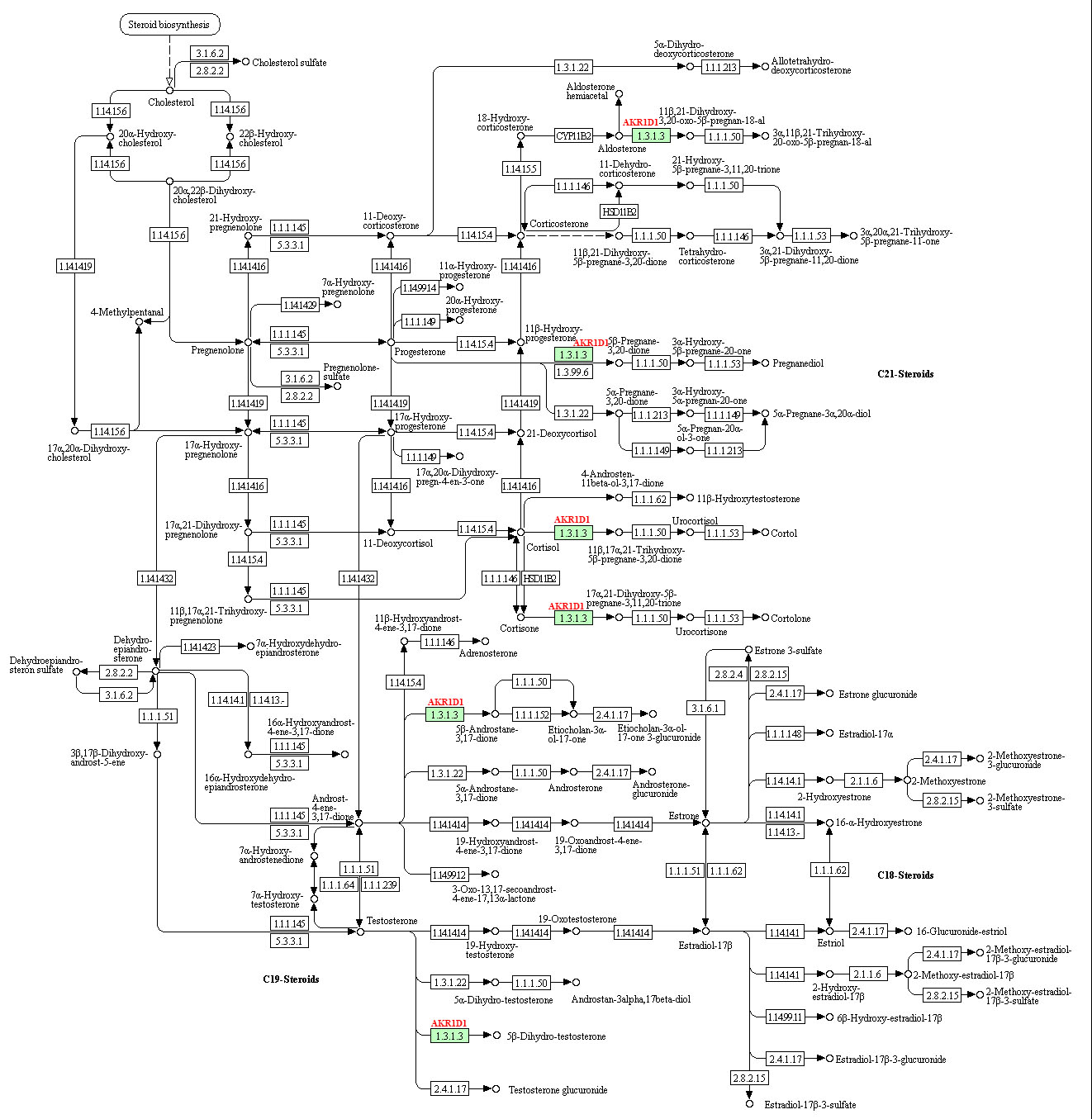


**F_IG_. S3.** Steroid hormone biosynthesis pathway (KEGG map00140).


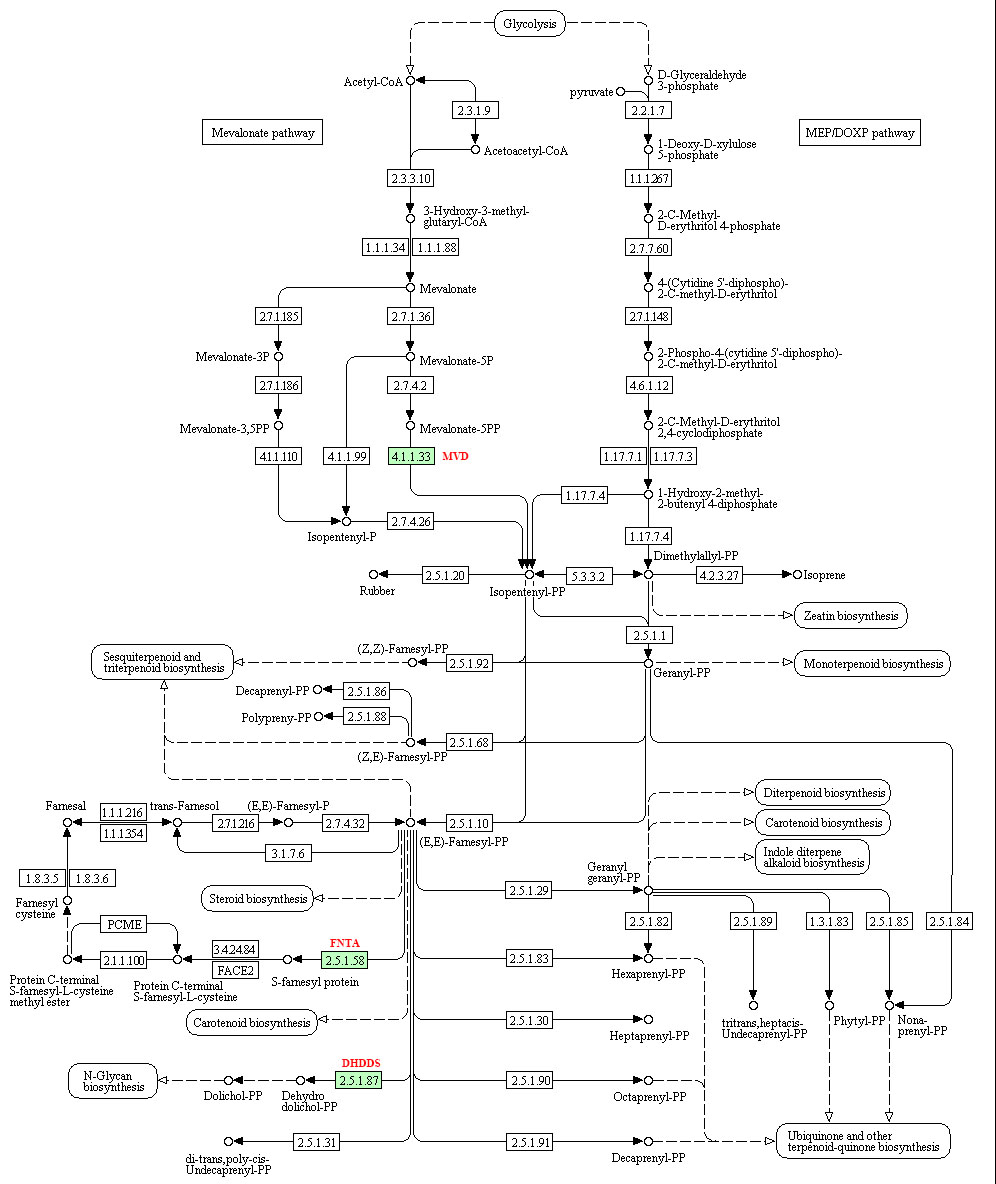


**F_IG_. S4.** Terpenoid backbone biosynthesis pathway (KEGG map00900).


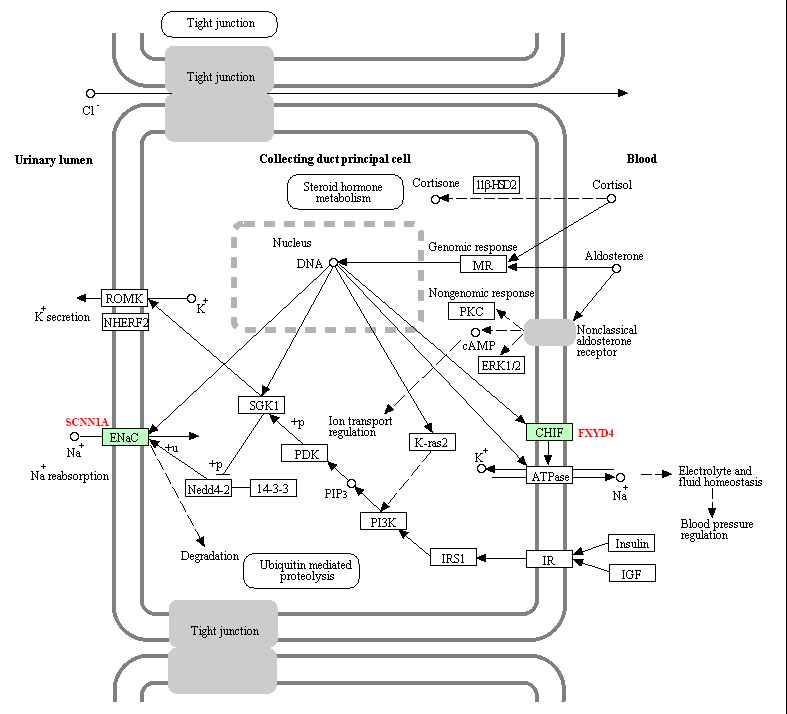


**F_IG_. S5.** Aldosterone-regulated sodium reabsorption (KEGG map04960).
